# Metagenomic analysis of planktonic riverine microbial consortia using nanopore sequencing reveals insight into river microbe taxonomy and function

**DOI:** 10.1093/gigascience/giaa053

**Published:** 2020-06-10

**Authors:** Kate Reddington, David Eccles, Justin O'Grady, Devin M Drown, Lars Hestbjerg Hansen, Tue Kjærgaard Nielsen, Anne-Lise Ducluzeau, Richard M Leggett, Darren Heavens, Ned Peel, Terrance P Snutch, Anthony Bayega, Spyridon Oikonomopoulos, Jiannis Ragoussis, Thomas Barry, Eric van der Helm, Dino Jolic, Hollian Richardson, Hans Jansen, John R Tyson, Miten Jain, Bonnie L Brown

**Affiliations:** Microbial Diagnostics Research Laboratory, Microbiology, School of Natural Sciences, National University of Ireland, University Road, Galway, Ireland H91 TK33, Ireland; Malaghan Institute of Medical Research, Gate 7, Victoria University Kelburn Parade, Wellington 6140, Wellington 6242, New Zealand; Quadram Institute Bioscience, Norwich Research Park, Norwich NR4 7UQ, UK; Norwich Medical School, University of East Anglia, James Watson Rd, Norwich NR4 7TJ, UK; Department of Biology and Wildlife, Institute of Arctic Biology, University of Alaska Fairbanks, 2140 Koyukuk Drive, Fairbanks, AK 9975-7000, USA; Department of Environmental Science, Aarhus University, PO Box 358, Frederiksborgvej 399, DK-4000 Roskilde, Denmark; Department of Plant and Environmental Sciences, University of Copenhagen, Thorvaldsensvej 40, 1871 Frederiksberg C, Denmark; Department of Environmental Science, Aarhus University, PO Box 358, Frederiksborgvej 399, DK-4000 Roskilde, Denmark; Department of Plant and Environmental Sciences, University of Copenhagen, Thorvaldsensvej 40, 1871 Frederiksberg C, Denmark; Institute of Arctic Biology, University of Alaska Fairbanks, 311 Irving 1 Building P.O. Box 757000 2140 Koyukuk Drive Fairbanks, AK 99775-7000, USA; Earlham Institute, Norwich Research Park, Norwich NR4 7UQ, UK; Earlham Institute, Norwich Research Park, Norwich NR4 7UQ, UK; Earlham Institute, Norwich Research Park, Norwich NR4 7UQ, UK; Michael Smith Laboratories and Department of Zoology, University of British Columbia, #301-2185 East Mall Vancouver, BC V6T 1Z4, Canada; McGill University and Genome Quebec Innovation Centre, Department of Human Genetics, McGill University, 3640 rue University, Montreal, Quebec H3A 0C7, Canada; McGill University and Genome Quebec Innovation Centre, Department of Human Genetics, McGill University, 3640 rue University, Montreal, Quebec H3A 0C7, Canada; McGill University and Genome Quebec Innovation Centre, Department of Human Genetics, McGill University, 3640 rue University, Montreal, Quebec H3A 0C7, Canada; Nucleic Acid Diagnostics Research Laboratory, Microbiology, School of Natural Sciences, National University of Ireland, University Road, Galway, Ireland H91 TK33, Ireland; Novo Nordisk Foundation Center for Biosustainability, Technical University of Denmark, Building 220, Kemitorvet, 2800 Kgs. Lyngby, Denmark; Department for Evolutionary Biology, Max Planck Institute for Developmental Biology, Max-Planck-Ring 5 72076 Tübingen, Germany; Norwich Medical School, University of East Anglia, James Watson Rd, Norwich NR4 7TJ, UK; Future Genomics Technologies B.V., Nucleus building, Sylviusweg 74, 2333 BE Leiden, The Netherlands; Michael Smith Laboratories and Department of Zoology, University of British Columbia, #301-2185 East Mall Vancouver, BC V6T 1Z4, Canada; UC Santa Cruz Genomics Institute, 1156 High Street, Santa Cruz, CA 95064, USA; Department of Biological Sciences, University of New Hampshire, 38 Academic Way, Durham, NH 03824, USA

**Keywords:** temperate river metagenomes, MinION, long-read, nanopore sequencing

## Abstract

**Background:**

Riverine ecosystems are biogeochemical powerhouses driven largely by microbial communities that inhabit water columns and sediments. Because rivers are used extensively for anthropogenic purposes (drinking water, recreation, agriculture, and industry), it is essential to understand how these activities affect the composition of river microbial consortia. Recent studies have shown that river metagenomes vary considerably, suggesting that microbial community data should be included in broad-scale river ecosystem models. But such ecogenomic studies have not been applied on a broad “aquascape” scale, and few if any have applied the newest nanopore technology.

**Results:**

We investigated the metagenomes of 11 rivers across 3 continents using MinION nanopore sequencing, a portable platform that could be useful for future global river monitoring. Up to 10 Gb of data per run were generated with average read lengths of 3.4 kb. Diversity and diagnosis of river function potential was accomplished with 0.5–1.0 ⋅ 10^6^ long reads. Our observations for 7 of the 11 rivers conformed to other river-omic findings, and we exposed previously unrecognized microbial biodiversity in the other 4 rivers.

**Conclusions:**

Deeper understanding that emerged is that river microbial consortia and the ecological functions they fulfil did not align with geographic location but instead implicated ecological responses of microbes to urban and other anthropogenic effects, and that changes in taxa manifested over a very short geographic space.

## Background

River ecosystems are Earth's biogeochemical powerhouses, and riverine processes largely are driven by the microbial communities that inhabit their water columns and sediments [1]. From an applied anthropogenic perspective, rivers are the lifeblood of human communities; recognition of this perspective led the New Zealand Government to grant legal personhood status to the Whanganui River as an indivisible and living whole [2]. Rivers provide food and drinking water and are a resource for agricultural and industrial use coupled with waste distribution, thereby reflecting a fingerprint of the total environment. Frequently, these services and activities are provided within an alarming proximity to each other. Regulatory authorities in many regions currently assess river “health” for management and monitoring of water resources using methods such as Biological Condition Gradient [3] and Index of Biotic Integrity [4, 5]. Such assessments score river “health” on the basis of occurrence of certain conditions, response to stress, and abundance of eukaryotic organisms. The recent focus on antimicrobial resistance (AMR) has highlighted the potential of AMR genes in aquatic microbes as a potential threat to human health. Complex microbial river water communities, often contributed to by human and animal activity, have more AMR genes than simple communities [6]. However, it is unclear at the moment which microbial resistance genes (or gene combinations) are a threat to human health and at what concentrations. Given that high-throughput sequencing has become economically viable for environmental monitoring, it is now possible to accurately characterize river metagenomes and determine the extent of taxonomic and functional variability among them. We can use this technology to monitor the levels of water-borne disease microorganisms and AMR genes, highlighting the need to include microbial community data in broad-scale ecosystem models.

## Data Description

It is likely that there is a correlation between river water microbial community composition, as determined by metagenomic sequencing, and river function and health [7–9]. Recent eco-genomic methods offer the capability to understand river ecosystems in greater detail, but for this approach to be widely used, particularly for real-time study in remote river systems such as Amazon, Klinaklini, Onyx, or Yarlung Tsanpo, field-deployable sequencing technology is necessary; the MinION has been demonstrated to be appropriate for on-site analysis [10]. We designed a study to evaluate river water metagenomes and the occurrence of riverine xenobiotic components, on a global scale, using the MinION portable sequencer paired with on-site data analysis. Assigning taxonomy and/or function for complex environmental river samples traditionally has been accomplished using whole-genome short-read sequences or short amplicons of 16S subregions [7, 8]. More recently, Johnson et al. [11] provided data illustrating that taxonomic resolution based on short reads of 16S subregions is less accurate than defining taxa using the full 16S gene sequence, primarily due to intragenomic differences among 16S gene copies. Meanwhile, high-throughput long-read analysis of complex mixtures using MinION and Pacific Biosciences platforms has become routine. Metagenomic analysis of mock communities using long single-molecule reads generated using Oxford Nanopore recently has been validated by comparing taxonomic assignment from long reads against taxonomy assigned using 16S ribosomal DNA genes [12, 13], illustrating that long-read metagenomes significantly match expected microbial taxonomic assignments and abundances. Bioinformatic study has shown that long, even error-prone, reads can significantly increase classification accuracy [13, 14]. Other critical assessments of the strengths and weaknesses of long-read–based metagenomic analysis have shown that these data can enhance our knowledge of ecosystem function coupled to microbial community structure [15, 16] and ultimately should help to more accurately model the biogeochemical processes driven by microbes. A deeper understanding of microbial diversity is needed to discern the implications on human health [17], e.g., the occurrence of antibiotic-resistant strains of bacteria in waterways that provide food and drinking water, and on productivity (e.g., nutrient cycling, crop irrigation, disposal of industrial and sanitation-related waste). Given that metagenomic analysis based on long-read data is promising, we envisioned a study with broad implementation of field-deployable long-read sequencing wherein we sampled a diverse set of 11 contrasting rivers and waterways across the globe. Here we describe a basic, high-level analysis of the results using multiple bioinformatic pipelines, providing all of the underlying raw sequence data for additional discovery and analysis by other researchers. We document the potential of long-read nanopore sequencing and real-time analysis of DNA obtained globally for environmental monitoring of the river biota, detection of microbes that respond to urban anthropogenic influence, and documentation of potential pathogens and AMR presence and diversity, with the aim of ultimately enabling water quality enhancement. We further believe that the methodology developed in this study provides a robust, small-footprint protocol that will facilitate broadening riverine metagenomic studies.

## Analyses

### Length and count statistics

**Figure 1:**
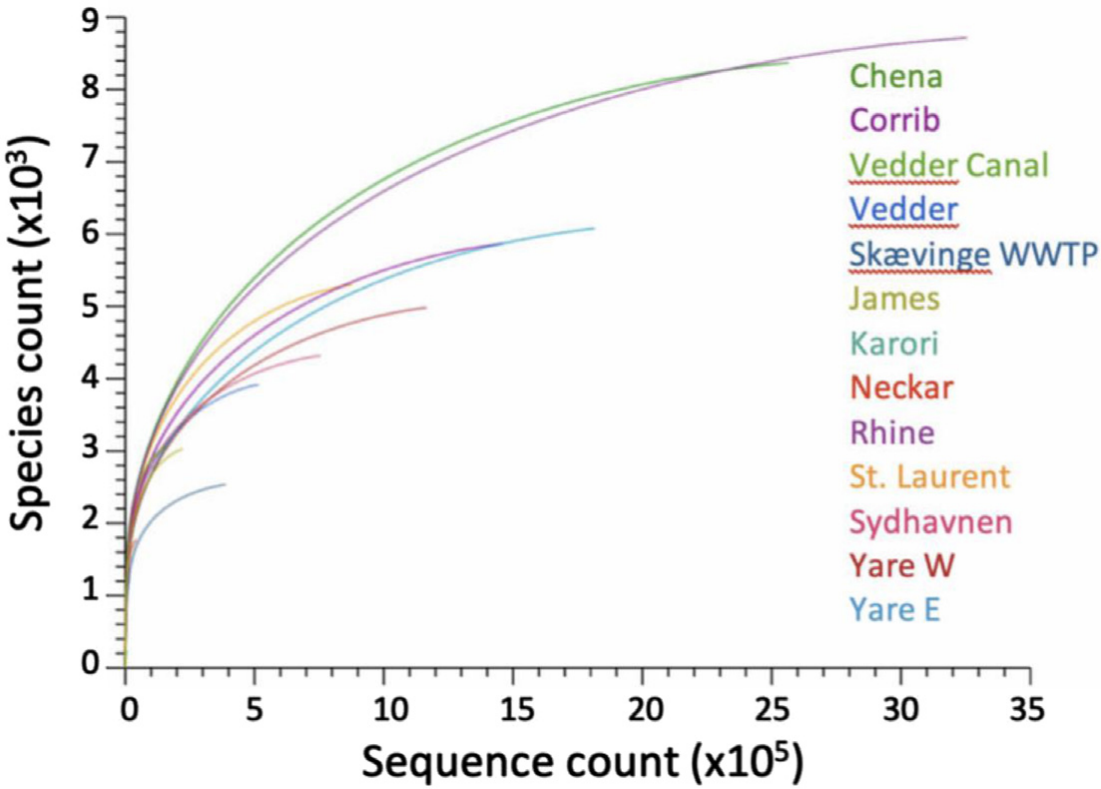
Rarefaction curves of the numbers of annotated species for 13 samples from 11 rivers and waterways based on MinION metagenomic data.

Libraries constructed by the MinION SQK-RLB001 kit consistently produced 2–5 kb fragments that yielded sequencing data sets averaging 1.1 ⋅ 10^6^ reads (3.8 ⋅ 10^9^ bases) of read length 3.4 ⋅ 10^3^ bp (Supplemental Fig. 1, Supplemental Table 1). Metagenomic results rarefaction (Fig. 1) indicated that although curves did not reach saturation, in general 0.5–1.0 ⋅ 10^6^ sampled long reads were adequate to capture most of the operational taxonomic unit (OTU) diversity of most samples.

### Negative control samples

The number of classified reads from the mapped negative control sample reads was very low in comparison with the number of river sample reads (∼0.1%). Within all negative controls, 33 families were identified above a 1% proportion (in any control sample) and negative control reads accounted for 0.04 ± 0.02% of the total read counts in the corresponding samples. Two of the negative control sample data sets had at most 1 read, and all but 4 sets had too few reads to be analysed in MG-RAST. Ultimately, there was no obvious trend that indicated the source of negative control reads being a result of consistent sample contamination during sample preparation (i.e., the isolation, library preparation, barcoding). Combined with the fact that the total number of negative reads was trivial in most cases and that there was no obvious pattern to their derivation, we performed no deeper investigation into the sources of negative control reads.

### Taxonomic diversity

Every read in every metagenome was assigned by MG-RAST to a predicted feature (a protein or ribosomal RNA). The mean proportion of reads was classified using the default criteria by MG-RAST to family 99.72% ± 0.09%, and for Kraken2 the average classification was 60.64% ± 3.75%. Metagenomic assignment of the whole-genome shotgun-sequenced (WGS) long-read data using One Codex resulted in much larger proportions of reads that were not classified (47–89%), which we hypothesize was a result of fewer taxa in the reference database and different assignment criteria. Thus, after preliminary analysis of the results, we opted for the MG-RAST and Kraken2 pipelines. Nearly all of the river metagenomes exhibited multimodal GC distributions (Supplemental Fig. 2), another indication of multiple domain representation that mirrors the GC representation in many other reports of freshwater environmental metagenomes [8, 18–20]. Reads for most river metagenomes were overwhelmingly assigned to the Bacteria domain at ≥94% with 1 exception, Sydhavnen at Copenhagen Harbour, where Virus accounted for >25% of the data and Bacteria only 68%. Eukaryotes were identified by MG-RAST in every metagenome at a level of ≤4% of reads, and Archaea were represented in all metagenomes by 0.2–6.0% of reads.

The 5 most common bacterial phyla observed were Proteobacteria, Bacteroidetes, Actinobacteria, Firmicutes, and Cyanobacteria. Proteobacteria were the most abundant prokaryote in most metagenomes (Vedder Canal was a distinct outlier where Bacteroidetes predominated), and within that group, the predominant taxon was the Burkholderiales, dominated therein by the Comamonadaceae comprising predominantly *Acidovorax* species (0.3–5% of assigned bacterial reads; iron and uranium oxidizers, nitrotolulene degraders, and plant pathogens) and *Polaromonas* (0.1–4% of bacterial reads; degraders of chlorinated alkenes and naphthalene). Another group that dominated the prokaryote hits was Bacteroidetes, composed overwhelmingly of *Flavobacterium* (0.5–35% of bacteria reads; extremely common in soils and freshwaters, and some are known disease agents). Moderately abundant prokaryotes were Actinobacteria, consisting nearly completely of Actinomycetales, fungus-like soil bacteria (0.4–41% of bacteria). Archaea occurred at an average of 1% of read assignments in all metagenomes except Chena River, which contained a high proportion of Archaea (6%); other published river metagenome studies recorded Archaea at the 1% level [7–9, 21]. Archaea groups detected were extremely similar across most metagenomes (most of which were Methanomicrobia, CO_2_ reducers); a notable exception was the metagenome for Sydhavnen (Copenhagen Harbour), where most of this group's representatives were instead Thaumarchaeota (noted for the ability to nitrify via oxidizing ammonia aerobically), dominated by *Nitrosopumilus*, a common player in the marine nitrogen cycle.

Across all metagenomes, 64 families were detected at ≥1% normalized abundance (Table 1). Alpha diversity across the 13 metagenomes ranged from a low of 135 species (Vedder Canal) to a high of 1,139 species (Chena River). Of the temperate urban rivers we investigated, Yare, Rhine, Neckar, Corrib, James, and St. Lawrence had average alpha diversity of 413 ± 29 SE species and exhibited family sets that conformed to the core groups that have been found to dominate other large temperate rivers and lakes [7–9, 22, 23]. The families observed in those 7 rivers concurred with what is expected on the basis of a general understanding of river ecology (covered more extensively below). Like those “typical” rivers, the metagenomes of Vedder River and Canal exhibited prokaryote families ubiquitous in soils and water environments, but these 2 samples stood apart owing to higher abundance of Cytophagaceae and Burkholderiaceae and lower abundance of Streptomycetaceae than in the other rivers. Metagenomes of Chena and Karori Rivers also exhibited prokaryote families ubiquitous in soils and water environments, but their consortia were dominated by different families than the other rivers. We also saw that Chena River showed evidence of hydrocarbon influence as the fourth, eighth, and 10th most abundant microbe families are important degraders of methylnaphthalene and BTEX (benzene, toluene, ethylbenzene, and xylene). Karori River was distinctive in that some of its most numerous microbial families were either marine (Cytophagaceae, Alteromonadaceae, and Vibrionaceae) or signified the presence of sewage (Enterobacteriaceae and Campylobacteraceae). The Skævinge wastewater inlet metagenome was unique, as expected, in that it was dominated by families (5 of the top 10) that are linked to sewage. The Sydhavnen metagenome was unique, as expected, owing to abundance of marine bacteria, marine-related viruses, and algae; only 2 of the major prokaryote families were typical of freshwater river ecosystems.

**Table 1:** Normalized proportions of 64 families that were detected at ≥1% in any of 13 river metagenome samples analysed by MinION, listed with the most commonly noted families at the top of the table

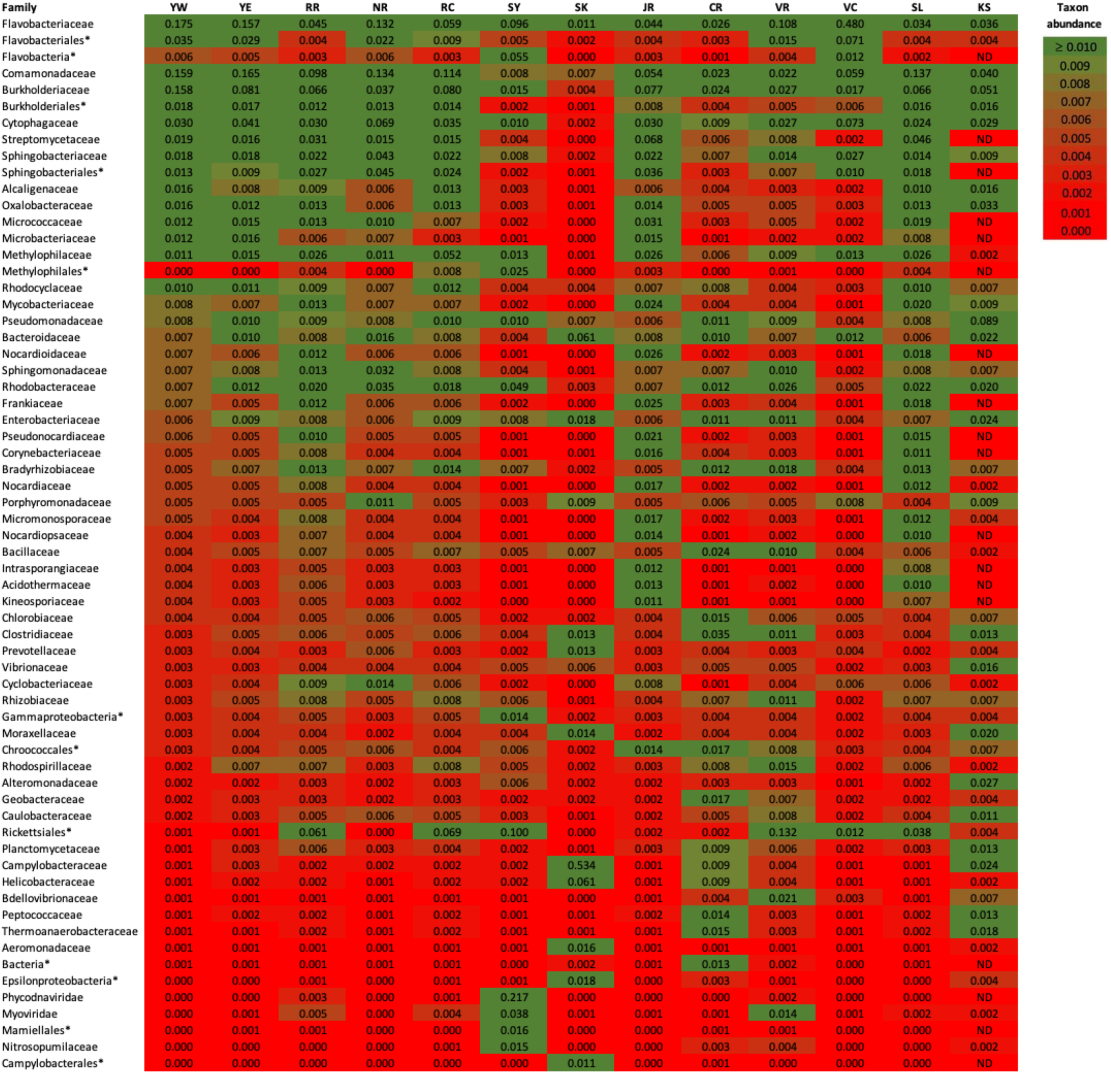

* includes OTUs in root where tools were unable to identify precise taxonomy. ND indicates family was not detected. Green-shaded cells indicate occurrence  ≥1%. River abbreviations are as follows: YW - River Yare West; YE - River Yare East; RR - Rhine River; NR - Neckar; RC - River Corrib; SY - Sydhavnen; SK - Skævinge; JR - James River; CR - Chena River; VR - Vedder River; VC - Vedder Canal; SL - St Laurent; KS - Karori Stream.

Of 1,249 genera classified, 69 occurred at ≥1% in any 1 of the 13 metagenomic samples and of those, 35 genera were represented on average at ≥1% in all of the samples. For the majority of samples, the most common OTUs were the bacterial genera *Flavobacterium, Polynucleobacter, Acidovorax, Polaromonas*, and *Streptomyces*. These microbes, known to be members of the “microbial loop” [24], are among the predominant drivers of water and soil ecosystem processes and have been documented as major contributors to the consortia of other aquatic systems [7, 9, 18, 22, 23, 25–27]. Three rivers exhibited very low frequencies of the common river OTUs. These exceptions included Chena (where *Clostridium, Bacillus*, and *Geobacter* predominated), Vedder (where *Pelagibacter* and Ricketsiales were most common), and Karori Stream (where most numerous were *Cellvibrio, Pseudomonas, Arcobacter, Bacteroides*, and *Burkholderia*). The least typical “river” samples were the wastewater influent at Skævinge (where the dominant genus was *Arcobacter*, 48.7%, followed by *Bacteroides* and *Campylobacter*, both of which are significant clinical pathogens) and Sydhavnen at Copenhagen Harbour (dominated by Prasinovirus and Phycoviridae, and having primary bacterial genera *Flavobacterium* and *Candidatus Pelagibacter*). Across all metagenomes, 5 genera that include some human pathogenic species were detected at ≥1% and many occurred at lower abundances. Present in all 13 metagenomes were *Campylobacter* (normalized proportion of 0.1–2.9%), *Clostridium* (0.3–3.2%), and *Prevotella* (0.2–1.3%). *Corynebacter* was in all except Karori Stream (0.1–1.6%) and *Helicobacter* present in all except Yare W and Neckar (0.1–1.5%). The fact that the taxonomic assignments for most rivers also implicated taxa that are anthropogenically relevant such as xenobiotic processors, disease-causing organisms, and pathogens of humans, fish, and crops is not novel. Xenobiotics and significant pathogens previously were observed for 1 of the rivers examined in this study (James River [7]) and have been documented using WGS data for other river metagenomes [9, 22].

The long-read WGS data provided important novel insight into the viral complements of some river metagenomes. Across the 11 rivers (13 sampling sites), the normalized proportions of viral reads ranged from 0.03% to 25.8% of read assignments. For 11 samples, virus accounted for <1% of reads, a finding typical of other river planktonic metagenomes [7–9, 18, 21]. Except for the 1 metagenome outlier, most virus read annotations were similar to T4-like virus (bacteriophages with some similarity to cloning vectors). The next most common were Phycoviridae (types that infect bacteria and archaea), followed by Iridoviridae (insect virus), and *Cafeteria roenbergensis* virus (a giant virus of marine phagotrophic flagellates). The notable outlier metagenome was Sydhavnen (Copenhagen Harbour), where >25% of all reads were mapped to viruses. These were not the type observed to dominate the other river metagenomes; instead the dominant types were Prasinovirus (52,116 annotations, observed e-values ≥1 ⋅ e^−9^, alignment lengths ≥38, identity ≥80.8%) and Phycodnavirus (e-values ≥1 ⋅ e^−7^, alignment lengths all >34, all showed >72% identity), which infect oceanic picoalgae, *Bathycoccus, Ostreococcus, Micromonas* (family Mamiellaceae), and other common groups of coastal green algae and cyanobacteria. Similar viral annotations were found in other samples but at 5–10 times lower abundance. Capture of this viral event may reflect the effect of oceanic water mixing with fresher water because salinity can influence the rate of viral decay and others have observed that algae transitioning from fresher to more saline waters experience increased viral abundances [28]. Alternatively, there could have been a recent bloom of picoalgae that advected onshore and was at the time of sampling in decline. The detection of such a high proportion of viral reads is notable in comparison to other halophilic WGS metagenomes where viruses generally are detected at ≤2% [29, 30] but actually has been seen recently as a significant benefit of the MinION sequencing method [31].

Despite the intentional methodological focus on picoplankton, a wide variety of eukaryotes (average 2% of read assignments) contributed to the river metagenomes. The same core phyla were detected across all samples, differing in proportion, and were highly similar to the taxa identified in other published riverine metagenomes [7–9, 18, 21]. Groups represented by ≥1% read assignments included Protists of various types (15%: amoebae, flagellates, ciliates), Ascomycota (12%: fungi), Chordata (12%: rodents and insectivores were predominant, followed by amphibians, fishes, and birds), Streptophyta (11%: predominantly castor, *Populus, Arabidopsis*, grape, followed by wheat, rice, corn, and mosses), Chlorophyta (10%: nearly all *Volvox* and *Chlamydomonas*, except for Sydhavnen where the predominant hits were marine prasinophytes), Cnidaria (10%: roughly equally split between Anthozoa and Hydrozoa, freshwater hydroids), Arthropoda (6%: nearly all hits were insects followed by spiders), Bacillariophyta (5%: diatoms), Apicomplexa (5%: nearly all parasitic), Nematoda (4%: equally split between free-living nematodes and parasitic filarial roundworms), and Basidiomycota (3%: in decreasing order, mushrooms, yeasts, smuts, and galls). In many cases, these observed taxa were telling of upstream agricultural and urban effects as has been observed in other river metagenomes [7–9].

**Figure 2:**
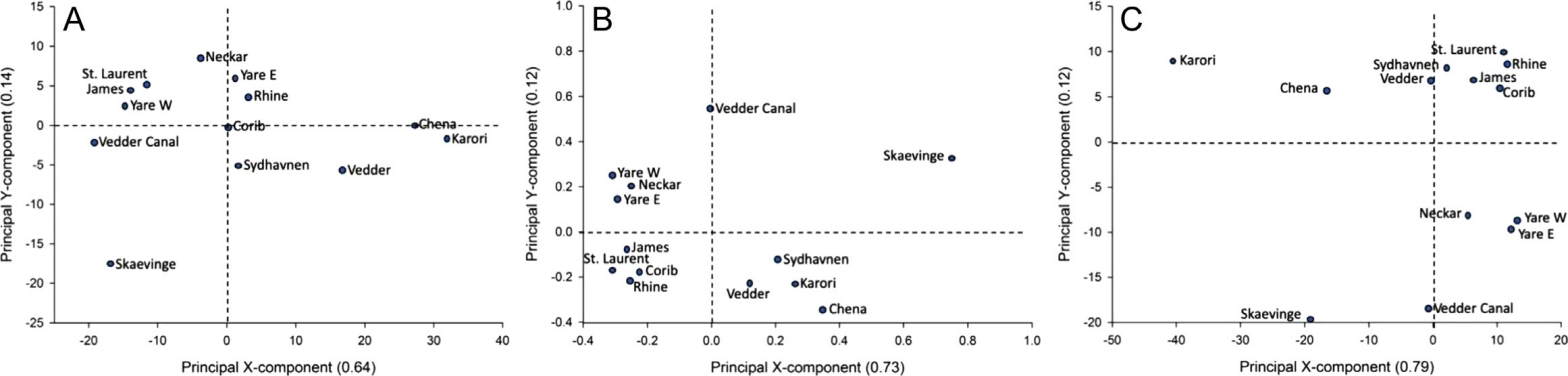
Concurrence of PCAs based on normalized data among 13 metagenomes from 11 rivers and waterways. A: Families annotated in Kraken2, B: Families annotated in MG-RAST, C: Subsystem Functions identified by MG-RAST.

River location by longitude, latitude, country, or continent was not reflected in the principal component analysis (PCA) grouping. The fact that both family and function PCAs yielded similar groupings and that those clusters did not reflect geography leads to the conclusion that the consortia and the ecological functions they fulfil may be more important than a river's precise location. An example can be seen in the paired sets of samples from Yare and Vedder that were collected up- and downstream of an urban center to examine the extent to which supposed anthropogenic effects on the waterways affected the river microbial consortia. Representative PCAs created from both Kraken2 results based on the annotated families imputed with missMDA and the MG-RAST normalized family frequencies ultimately clustered River Yare samples collected east and west of Norwich, suggesting that both samples have similar metagenomic profiles (Fig. 2A and B). Conversely, Vedder River and Vedder Canal metagenomes that were separated by a similar distance as the River Yare samples did not cluster.

### Functional diversity

**Figure 3:**
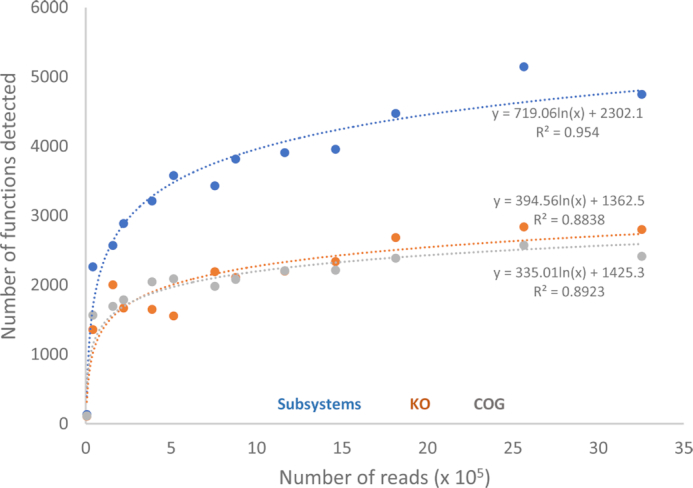
Detected Subsystems, KO, and COG functions versus read count for 13 river and waterway metagenomes.

There were 2,889 Clusters of Orthologous Groups (COG) pathways, 3,806 KO pathways, and 6,554 Subsystem Functions annotated across the 13 metagenomes. The distribution of detected functions versus sequence count was logarithmic for hits from all 3 databases (Fig. 3), indicating in each case that ∼2.5 ⋅ 10^5^ long reads seem to be necessary to adequately diagnose river function potential using the MinION sequencing platform, a range well within the read output for 9 out of 12 experiments in this study. The long-read data yielded assignments for functions of Bacteria comparable to North American river bacterial functions detected using data from other sequencing platforms [7–9], the vast majority of which were associated with basic cellular housekeeping (Table 2).

**Table 2:** Normalized percent abundances of functions annotated through KO and COG

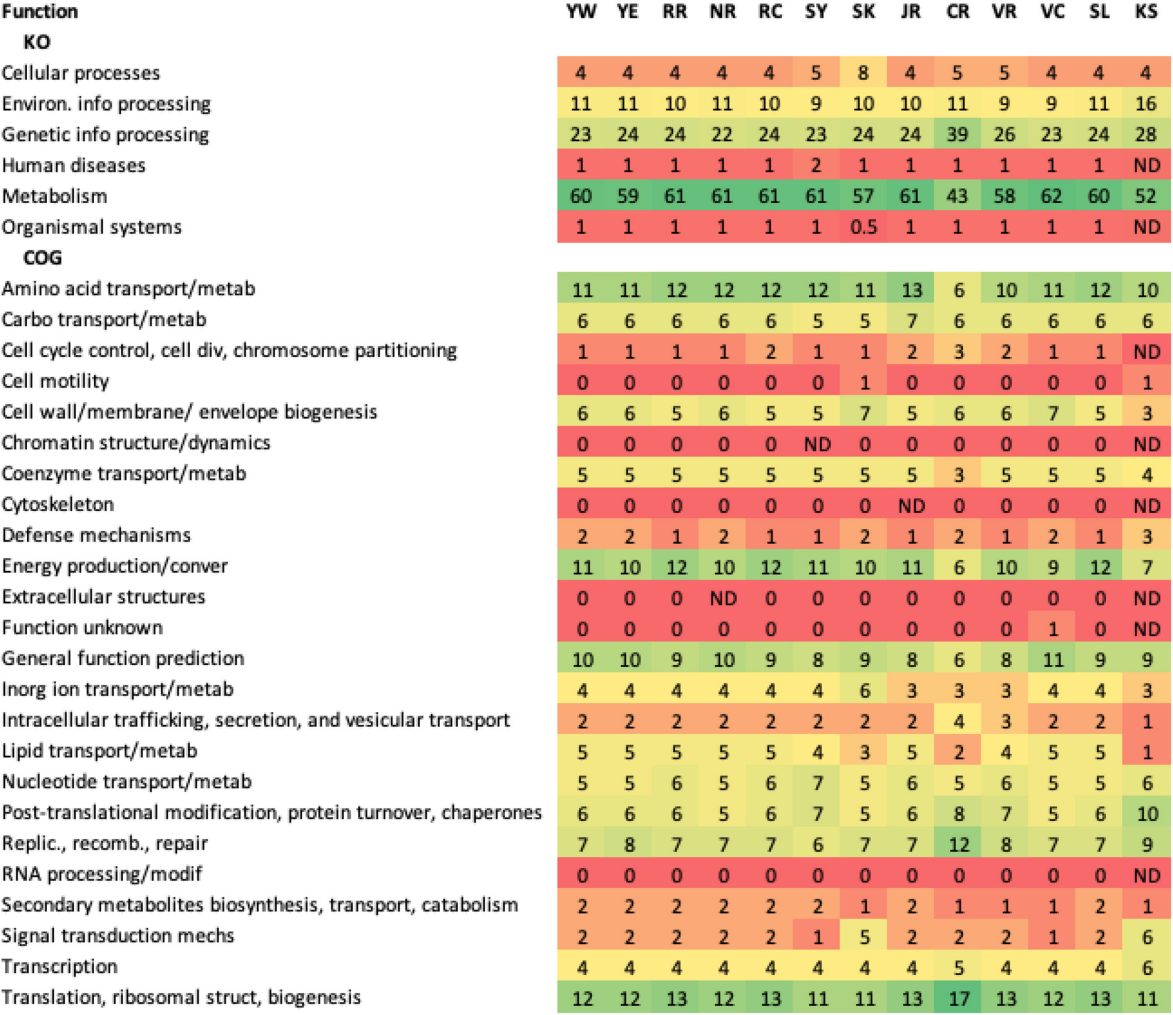

River abbreviations are as shown in Table   1. Colours indicate proportions: green - high; yellow/orange - intermediate; red - function was < 1% or not detected (ND).

Prior studies suggest that wastewater release contributes to river resistomes [22, 32–34] and as mentioned above, we found signals of urban sewage in all of the metagenomes examined, at low abundance in most but unexpectedly high in others. We also detected other functions that indicated how these river consortia respond to the putative anthropogenic influences on these waterways (Table 3). For example, across the river metagenomes, 24 different mechanisms (0.8% of the COG processes detected) were related to antibiotic or multidrug resistance (AMR), toxins, or virulence. The most prevalent of those were AMR pathways dominated by the cation/multidrug efflux pump and the ABC-type multidrug transport system (ATPase and permease components). According to the SEED viewer, 24 genes were detected that direct transporting and processing of heavy metals (arsenic, copper, cobalt, zinc, lead, and cadmium; those for copper were highly represented). Of all KO functional pathways detected, 60 (24% of all annotated pathways) were related to processing of xenobiotic substances or to human or plant pathogens and diseases. The xenobiotic processes were dominated by degradation of benzoate (PATH:ko00362), chlorocyclohexane and chlorobenzene (PATH:ko00361), aminobenzoate (PATH:ko00627), nitrotoluene (PATH:ko00633), atrazine (PATH:ko00791), and dioxin (PATH:ko00621). Similar observations were made for an earlier James River metagenome previously analysed using different WGS sequencing technologies [7]. The PCA analysis for families (Fig. 2A and B) grouped samples in a nearly identical fashion as for Subsystem Functions (Fig. 2C), giving support to the contention that microbial function is driving differences among river and waterway metagenomes, not location.

**Table 3:** Normalized percentage abundances, where a function was represented at ≥0.1% of annotations, of KO pathways detected related to processing of xenobiotic substances or to human or plant pathogens and diseases, and of COG pathways relating to antibiotic or multidrug resistance, toxins, or virulence

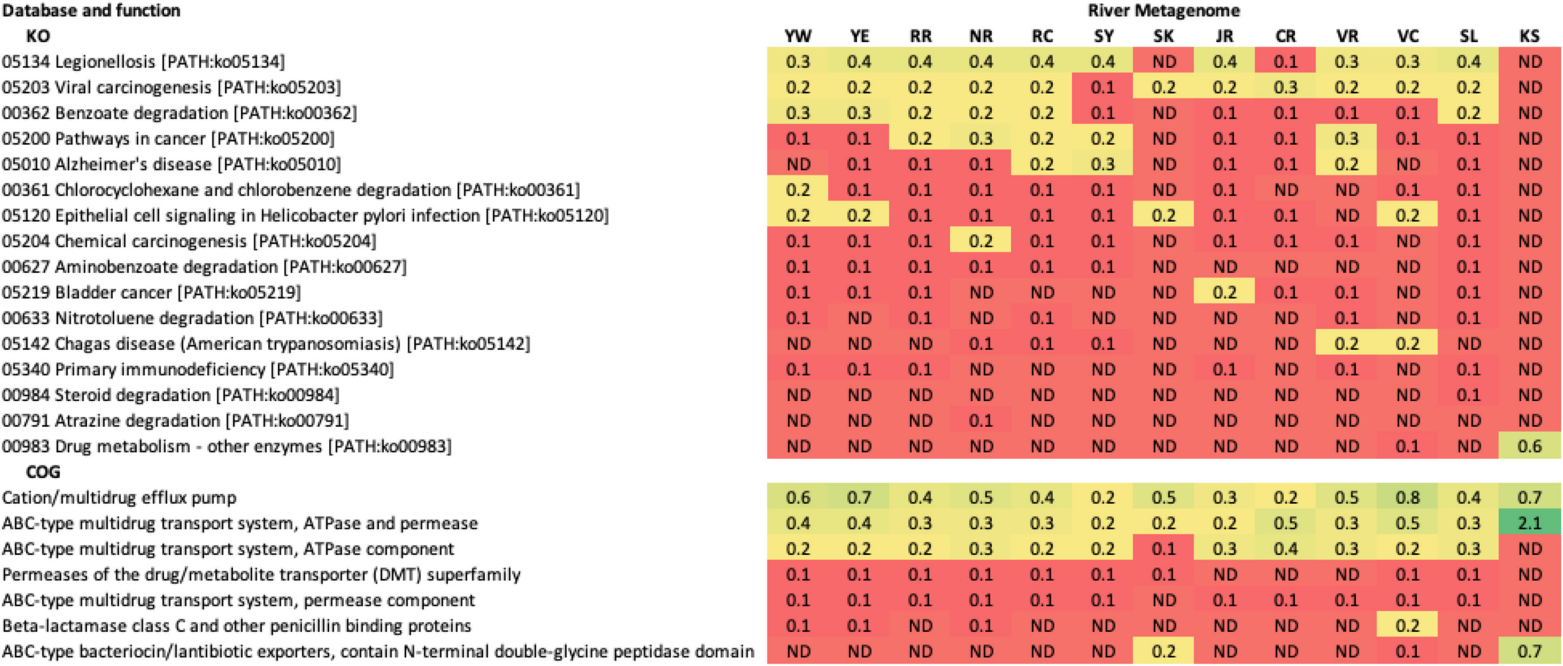

River abbreviations are as in Table   1. Colours indicate relative abundances: green - ≥1%; yellow/orange - intermediate; red - ≤0.1% / not detected (ND).

## Discussion

### Long-read metagenome analysis for river taxonomy and function

Because rivers are used extensively for anthropogenic purposes (drinking water, recreation, agriculture, and industry), it is essential to understand how these activities affect the composition of river microbial consortia. Such understanding could be facilitated on a massive scale if there were a broadly applicable means for spatiotemporal river testing that could produce an unbiased representation of the microbial community. This would be especially helpful to document the presence, distribution, function, and evolution of epidemiologically significant organisms. Nanopore technology has enabled the application of long-read nucleotide sequencing in a number of ecogenomic applications and holds promise for just such testing in river systems. We found that DNA sequence data from river water produced by the portable MinION device joined with metagenomic analysis provided a sensitive platform for investigating the diversity and ecological function of microbiota inhabiting Earth's rivers and waterways. Because we used a WGS approach, we also captured signals of organisms inhabiting the riparian zones and larger watershed. We even captured genomic signals of an algal/viral event in an eighth waterway sample (Sydnavnen). Detecting biodiversity of rivers and their watersheds previously has been reported using short-read whole-genome and targeted strategies [7–9, 35]. This study illustrates that similarly comprehensive results are obtained using long-read sequencing. By sequencing DNA from rivers on 3 continents using MinION rapid sequencing and analysing those data with both local and cloud-based tools, we obtained detailed results on taxonomy and function that implied just how distinct and ecologically responsive those river system microbiota are. Furthermore, the study highlights the added value of portable WGS paired with on-site data analysis, which allows us to avoid assembly approaches and resolve possible gene synteny directly from sequencing reads rather than from contigs, to avoid the artifacts and biases inherent to PCR (e.g., false-negative results, polymerase error, primer mismatch, saturation) and to avoid the need for multiple sample processing to investigate different “fractions” (e.g., 16S, 18S, viral, COI).

In addition to the utility of this approach for studying river consortia, we present data that illustrate its potential for monitoring for anthropogenic effects on river biota, detecting pathogen presence and diversity throughout river systems, judging risk associated with water uses, and hopefully enhancing water quality. Despite <1× coverage of taxa and the precautions espoused by Gweon et al. [36], our analyses exposed previously unrecognized aspects of microbial biodiversity in 4 waterways, where the metagenomes deviated from the expected suite of taxa; several illustrated marine influence, some showed taxa responsive to hydrocarbon pollution, and others had strong signals of taxa related to sewage and AMR gene matches linked to antibiotic resistance. We suggest that the most common OTUs (i.e., groups that occur at ≥1% of the detected consortia) seem to be good indicators of the extent to which rivers and waterways are responding to anthropogenic impacts.

Additional bioinformatic development is necessary, however, to ultimately support a field-deployable sequencing device paired with deeply informative statistical analysis that has the capability of rapidly and comprehensively detecting microbes. Comparing widely available bioinformatic tools to analyse the river metagenomes revealed that One Codex resulted in a very high proportion of unclassified reads and, as the parameters were not adjustable, was unsuitable in our hands for this analysis. Using the local Kraken2 sequence classification system, we had fewer unclassified reads and detected a wider diversity of organisms in each river, concurrent with levels of diversity detected by other sequencing technologies. The web-based MG-RAST service provided zero unclassified reads and deeper, more comprehensive information, particularly with regard to xenobiotics, pathogens, and AMR. Comparative PCA analyses of these river metagenomes using both Kraken2 and MG-RAST data, at both the family and function levels (Fig. 2), yielded highly similar groupings, indicating that geographic proximity is far less important than the ecological functions being carried out by the predominantly microbial consortia. Such relationships among microbial consortia and urban/agriculture effects have been noted in other watersheds wherein, as for studies *op. cit*. here, riverine microbial consortia varied as a function of land use and environmental quality [21, 37].

### Substantial compositional differences between geographically proximal sites

The differences among stories told by the metagenomes were striking in that these microbial “snapshots” (Supplemental Fig. 3) and jobs that the microbial consortia in rivers and waterways are performing (Tables 2 and 3) give signals that we believe could be used to enhance river management. For example, although Yare River samples collected west and east of Norwich yielded highly similar taxonomy and function, our 2 Vedder (River and Canal) samples, which were geographically closer than the Yare samples were to each other, yielded distinct taxonomic and functional arrays. The 2 Vedder sites had substantially different flow and anthropogenic impact. Vedder River is an actively flowing, relatively natural river area with a rock and gravel bottom and fed by an upstream lake of mountain rain and snow-melt runoff, whereas the downstream Vedder Canal is a channelized deep artificial canal with minimal current flow, a mud bottom, and high sediment load. These physicochemical differences manifested in radically different consortia and therefore different predicted river functional pathways, illustrating how informative metagenomic analysis can be for investigating the interaction between geophysical site composition and bacterial community composition. The understanding derived from such observations will be particularly useful for river ecosystem management and, indeed, is emerging as an important component of global change models.

## Potential Implications

We demonstrate that yields of >1 M reads (i.e., 1 Gb data) are easily achievable with MinION, and further that yields of >10 Gb are possible using the rapid PCR barcoding kit, thus allowing for multiplexing of environmental samples. If one were planning multiplexed runs, our study shows that this process should be sufficient for a quick indication of high-level metagenomic diversity. The analyses presented here illustrate that at the present average output, users interested in accurate assessment of taxonomy and function should strive for ≥250,000 reads per sample. Readers should be aware that nanopore sequencing is a new and disruptive technology in a state of constant improvement. As such, by the time of this publication, the approach presented here is anticipated to already have been enhanced through modification of MinION flow cells, library chemistry, and bioinformatic capabilities.

## Methods

### Global river water sites

For this study, 11 diverse global riverine waterways (Supplemental Table 2, Supplemental Fig. 4) were analysed to compare the metagenomic diversity of microorganisms identified and to garner an initial understanding of microbial resistance genes present. In Europe, these rivers included River Yare (collections west and east of Norwich, UK), River Rhine (Bimmen Netherlands/Germany), Neckar River (Tübingen, Germany), River Corrib (Galway, Ireland), Sydhavnen (Copenhagen, Denmark), and the Skævinge wastewater treatment plant (Skævinge, Denmark). In the USA, rivers sampled included James River (Richmond, VA) and Chena River (Fairbanks, AK). In Canada, rivers sampled included Vedder River (Vancouver, BC) and St. Lawrence River (Montreal, QC). A final site sampled in this study was the Karori Stream in New Zealand (Wellington).

River Yare is ∼84 km long and flows from the west of Norfolk to the east coast, passing through the city of Norwich (urban population ∼300,000). Outside the city center, most of the rest of the land that the river traverses is rural, with arable agriculture and tourism (sailing, motorboats). Two sample locations from this river were analysed. This eastern sample was collected from the riverbank by a public house in a small village, ∼3 km downstream from the edge of Norwich. The upstream sample was obtained beside the University of East Anglia sports fields in a suburban area of Norwich.

River Rhine is one of Europe's largest rivers, with a length of 1,230 km. The sampling site was a pier extending into the river where the surrounding land was rural in character with mainly agricultural farmland. Upstream of the sampling point (the lower Rhine) consists of one of Europe's largest industrial and urban areas, the Ruhr area (urban population ∼5 million).

The Neckar River flows 362 km in Germany from the Black Forest to the Rhine River. Upstream of the sampling site near Tübingen (urban population ∼100,000), the river flows through an area with a mix of villages, farmland, and forest. The sample was collected from a multi-lane divided bridge.

River Corrib in the west of Ireland is one of the shortest rivers in Europe. It flows 6 km from Lough Corrib to the Atlantic Ocean. Samples for analysis were taken from the Upper Corrib region, 2 km upstream from the Galway city center (rural population ∼80,000) in an unpopulated area where minor cattle and sheep grazing occurs (pastoral farming).

Sydhavnen is a suburb of Copenhagen situated along Sluseløbet Canal and Copenhagen Harbour, directly connected to the Øresund (a sound that forms the border between Denmark and Sweden) in the northeast and the Baltic Sea in the southwest, stretching ∼8 km. In addition to oceanic influence at both ends, the waterway, not technically a river, has heavy urban and transportation influence. The sample was collected in Sluseløbet Canal, beneath a bridge in Sydhavnen (urban population ∼780,000), ∼5 km below the Øresund.

Influent wastewater to the Skævinge wastewater treatment plant, not technically a river, was included to act as a control for high anthropogenic impact because it was assumed to contain human-associated bacteria. Skævinge wastewater treatment plant is located in a rural area of Zealand, Denmark, and treats wastewater from residential, industrial, and agricultural areas.

James River runs 560 km from the Appalachian Mountains to the Atlantic Ocean. The sampling site was in downtown Richmond, Virginia, and is known to have heavy urban, industrial, and transportation influence. Near the site is one of the largest combined sewer overflow systems on the mid-Atlantic east coast of North America, and the site also receives local permitted input from construction, power plants, failing sewer systems, and industrial discharges, resulting in elevated levels of polychlorinated biphenyls (PCBs). Upstream watershed activities include >170 active industrial discharges and >90 permitted pre-treatment discharge sites.

Chena River, the northernmost river in our sample, is spring-fed, stretches 160 km, and collects water from interior Alaska. Samples were taken on the downstream side of the Moose Creek Dam and upstream of the populated areas of the Fairbanks North Star Borough.

Vedder River is a continuously flowing river 80 km in length that drains Chilliwack Lake, itself snow fed from the Cascade Mountains. The immediate area where the sample was collected was near Chilliwack suburbs and exhibited constant current flow. A second sample was collected from the Vedder Canal, a downstream artificial canal that drains into the Fraser River and is a main area for both swimming and salmon fishing. The canal is surrounded by earthen dykes that are immediately adjacent to active farming land on both flanks; there was little visible water movement at the time of sample collection.

Saint Lawrence River, running nearly 1,200 km, is the third-longest river in Canada. The sampling site was located off of Jean-Drapeau Park ∼5 km downstream of downtown Montreal (urban population ∼1.8 million). This site is close to a municipal routine sampling site named FLS190 where past data have been collected and are available [38].

Karori Stream, the southernmost river in our sample, traverses ∼10 km through Wellington, New Zealand, with headwaters in bush and suburban areas, discharging into the sea at Wellington's south coast. The “Karori Stream at Makara Peak Mountain Bike Park” sampling site in Wellington is one of the Greater Wellington Regional Council's regular river sampling sites, in the middle reaches of the Karori Stream. There it also has some suburban and transportation influence.

### Sample collection, DNA extraction, library generation, sequencing technology

Between April 2017 and October 2018, 12 laboratories with personnel exhibiting a wide diversity of skills and experience followed the standardized protocol [39] outlined below for the filtering and extraction of DNA from shallow waters of 11 riverine waterways. Water samples were taken at 0.5–1 m depth during daylight hours at a time when neither drought nor recent excessive precipitation events occurred within 1 week preceding sample collection. River water (2–4 L) was collected for filtration in sterile collection bottles and was processed immediately or stored at 4°C for prolonged transportation time or until ready for filtration. The water samples were subsequently processed through a GF/C filter to remove suspended solids, particles, etc. (size retention: 1.2 µm). The water recovered after GF/C filtering was subsequently filtered through a 0.22-µm Durapore filter to capture microorganisms present. Upon completion of all filtering, nucleic acid was recovered using a modified procedure combining enzymatic lysis and purification using a DNeasy PowerWater DNA Isolation Kit (Qiagen, Germantown, Maryland, USA). Briefly, each filter was aseptically transferred to a 5-mL tube. To this tube, a lysis mix was added, which contained 1 mL of PW1 (DNeasy Power Water DNA isolation kit) and a previously described enzyme cocktail [40] containing 100 µL lysozyme (10 mg/mL, Sigma-Aldrich, St. Louis, Missouri, USA), 12 µL mutanolysin (25 KU/mL, Sigma-Aldrich), and 6 µL lysostaphin (4,000 U/mL, Sigma-Aldrich). The 5-mL tube with the lysis mixture was subsequently incubated at 37°C for 1 hour, with gentle agitation to facilitate washing of the filters.

Steps 8–23 of the “experienced user protocol from the DNeasy Power Water DNA isolation kit” were followed. The eluted DNA was quantified using a Qubit fluorometer with the dsDNA HS kit. After quantification, a 0.4X SPRI bead clean-up of ∼100 ng neat DNA was performed and eluted in 20 µL molecular grade water (or Tris-Cl pH 8–8.5). Subsequently 10–50 ng of DNA was used in conjunction with the Rapid Low Input by PCR Barcoding kit (SQK-RLB001, Oxford Nanopore, Oxford, United Kingdom) in accordance with manufacturer's protocols to prepare WGS libraries for use with a MinION device with minor alterations outlined below. A barcoding kit was chosen to facilitate multiplexing of negative controls and DNA from river samples to determine whether any contamination was present during the processing of the river water templates. Modifications for the library preparation were (i) 10–50 ng of input DNA and 2.5 µL of fragmentation mix (FRM) were used for the tagmentation/fragmentation reaction and nuclease-free water was used to make the volume up to 10 µL; (ii) for the PCR reaction, 20 cycles were used and the PCR reaction volumes were doubled. When multiplexing (negative filter and DNA from associated river samples), PCR products were pooled together in equal volumes, then subjected to a 0.6x AMPure XP bead wash and eluted in 12 µL of the buffer recommended in the manufacturer's instructions (10 μL 50 mM NaCl, 10 mM Tris-HCl pH 8.0).

After amplification a number of quality control checks were implemented to ensure that successful library preparation was achieved. The quantity was assessed using the Qubit fluorometer dsDNA HS kit and DNA quality and estimated size distribution were subsequently determined via Tapestation, Bioanalyzer, or agarose gel. Following quality control steps and removal of unincorporated primers, sequencing adapters were added to the mix and a room-temperature ligation-free reaction was carried out to link the adapters to the prepared DNA template. This prepared library (100–200 fmol) was then loaded into the MinION flow cell (R9.4) in accordance with manufacturer's guidelines and the unit was run for a full 48 hours of sequencing.

### Sequence processing, annotation, post-processing, and data analysis

WGS reads were processed for basecalling and quality control filtering using Albacore version 2.1.10 (Oxford Nanopore), and adapters were removed from the resulting DNA sequence reads using Porechop version 0.2.3 [41] using the command-line parameters “porechop -i $INPUT -o $INPUT.porechop.fq –format fastq -t 32 –discard_middle” . In a number of instances where replicate runs were performed for the same sample, the replicate data sets were pooled. The final adapter-trimmed data are accessible in both EBI (fast5) and MG-RAST (FASTQ, Supplemental Table 2). Read lengths for sample-pooled FASTQ files were determined using a custom fastx-length.pl script and processed into cumulative read length distribution plots and digital electrophoresis plots using a custom length_plot.pl script [42].

To classify the sequence data for the purposes of identifying the microbial community in each water sample and to consider how these contribute to the ecology of each river ecosystem, FASTQ data initially were submitted to One Codex (based on the recommendation of [12]), an online pipeline that identifies microbial sequences using a *k*-mer–based taxonomic classification algorithm, typically used for short-read data. The 2018 database chosen for analysis comprises a reference database that included ∼80,000 bacterial, viral, fungal, and protozoan genomes. Reads also were processed using Kraken2 [43], a different *k*-mer–based sequence classification algorithm optimized for long-read sequences, which uses a publicly available pre-compiled genome database of bacteria, fungi, and viruses from RefSeq [44]. Last, sequences were uploaded via the command-line API and processed using MG-RAST [45], a pipeline that for whole-genome sequences first performs a protein similarity search between predicted proteins and database proteins and then provides bioinformatic tools to predict ribosomal DNA, gene, and protein functions with default parameters as follows: e-value 1 ⋅ e^−5^ (probability of chance incorrect annotation), identity 60%, and a minimum alignment length of 15 (10–60 bp is common). Pavian plots of the representative taxa for each metagenome [46] were constructed using the Kraken2 output. Using both MG-RAST and Kraken2 taxonomy results and the Bray-Curtis distance matrix among normalized family counts, PCA was implemented, with 1 exception: for PCA on Kraken2 results, families were filtered to only include those that had <20% of samples with missing or zero counts.

To evaluate putative ecosystem-related functions from the reads, the MG-RAST server was used to compare data sets to 3 controlled annotation namespaces: Subsystems, KO, and COG proteins. Normalized function data for each river sample were compared using PCA in MG-RAST (Subsystems Level 1, Minkowski distance matrix).

## Availability of Supporting Data and Materials

Raw signal FAST5 and FASTQ files are available from the ENA via accessions Nos. PRJEB34137 and ERP116996. Basecalled FASTQ read sets are archived in MG-RAST. Supplemental tables and figures are available. Custom codes can be found at [47]. Other data further supporting this work can be found in the *GigaScience* repository, GigaDB [48].

## Additional Files

**Table S1**. Long-read metagenome properties and downstream analysis summary. L50: length of the shortest read in the set of the longest 50% of base-called data; N50: number of reads in the set of the longest 50% of base-called data; rRNA: number of reads that contain ribosomal RNA genes; Features: predicted proteins; CDS: identified proteins; Subsystems: number of reads assigned to all Subsystem Level 1 functional categories; Anthro: percentage of reads with predicted protein functions annotated to Virulence, Disease and Defense, Phages, Prophages, Transposable Elements, Plasmids, Metabolism of Aromatic Compounds, and Stress Response; α-Diversity: estimated from the distribution of the species-level annotations.

**Table S2**. Site, study, and sample information. The notation “NA” in parentheses is included where negative read set files included too few reads to be analysed in MG-RAST. Where accessions are given in parentheses, those refer to the barcoded negative control data. All FASTQ files are available from ENA via accessions Nos. PRJEB34137 and ERP116996.

**Figure S1**. Read length distribution plots for sample (A and B) and negative (C and D) sequencing libraries. The cumulative sequenced bases plots (A and C) allow read length percentiles to be identified; read N10, N50, and N90 are indicated on the plot by vertical lines and a pair of circles. The digital electrophoresis plots (B and D) show the distribution of read lengths in the libraries, as might be seen via gel electrophoresis. The sample libraries generally show a very tight read length distribution (except for the low-count samples, Karori and Neckar), whereas the negative samples have platykurtic length distribution curves. Not shown in panel D: James-neg (because only 1 sequence, *H. sapiens*, ATP synthase, was detected, 1.67 kb).

**Figure S2**. Proportion of GC in each of 13 metagenomes from 11 rivers. Color scale same as in Supplemental Fig. 1.

**Figure S3**. Representative taxonomy shown as Pavian plots for each of 13 metagenomes from 11 rivers.

**Figure S4**. Full-resolution location maps (insets are reduced-resolution Pavian plots) to illustrate the range of sites sampled and the relative diversity of taxa identified in 13 riverine metagenomes.

## Abbreviations

AMR: antimicrobial resistance; API: Application Programming Interface; ATP: adenosine triphosphate; bp: base pairs; COG: Clusters of Orthologous Groups; ENA: European Nucleotide Archive; Gb: gigabase pairs; GC: guanine-cytosine; kb: kilobase pairs; KEGG: Kyoto Encyclopedia of Genes and Genomes; KO: KEGG Orthologues; MG-RAST: Metagenomics Rapid Annotation Using Subsystems Technology; OTU: operational taxonomic unit; PCA: principal component analysis; WGS: whole-genome shotgun sequenced.

## Competing Interests

B.L.B., D.E., J.O.G., J.R.T., M.J., and H.J. have received financial and non-financial benefits from Oxford Nanopore Technologies. Flow cells and library preparation kits were provided for the study by Oxford Nanopore Technologies at a group reduced charge.

## Funding

A.L.D. and D.M.D. were supported by Alaska BLaST through the National Institute of General Medical Sciences of the National Institutes of Health under awards UL1GM118991, TL4GM118992, and RL5GM118990 and Alaska INBRE, an Institutional Development Award (IDeA) from the National Institute of General Medical Sciences of the National Institutes of Health under grant No. P20GM103395. T.K.N. and L.H.H. were supported by AUFF- NOVA grant AUFF-E-201 7-9-38. R.M.L. and D.H.'s contribution to this research was funded by BBSRC grants BB/J004669/1 and BB/CSP17270/1. N.P.'s contribution was funded by BBSRC grant BB/M011216/1. JR was supported by Genome Canada Genomics Technology Platform grant, the Canada Foundation for Innovation (CFI) and the CFI Leaders Opportunity Fund (32,557), Compute Canada Resource Allocation Project (WST-164-AB) and Genome Innovation Node (244,819). B.L.B.'s contribution to this research was funded by NSF DEB award No. 1,355,059. T.P.S. and J.R.T. were supported by the Canada Research Chair in Biotechnology and Genomics-Neurobiology (T.P.S.), the Canadian Institutes of Health Research (No. 10,677; T.P.S.), and the Koerner Foundation (T.P.S.).

## Authors' Contributions

The study was conceived by J.O.G., M.J., J.R.T., K.R., and B.L.B.; M.J. coordinated the collaboration; B.L.B., D.M.D., D.E., A.L.D., R.M.L., D.H., N.P., H.J., L.H.H., T.K.N., J.O.G., H.R., E.v.d.H., A.B., S.O., I.R., J.R.T., T.P.S., and K.R. sampled rivers and performed sequencing; D.M.D., D.E., A.L.D., H.J., J.O.G., B.L.B., M.J., and J.R.T. analysed and interpreted data; K.R., L.H.H., T.K.N., and J.O.G. developed and tested the protocol; D.J., M.J., and J.R.T. performed base-calling and data transfers; B.L.B. and D.E. investigated One Codex analysis; J.R.T. performed all Kraken2 analysis; B.L.B. and D.E. conducted PCA analyses; B.L.B., H.J., and D.M.D. uploaded and performed MG-RAST analysis; B.L.B., D.E., D.M.D., A.L.D., R.M.L., L.H.H., T.K.N., K.R., T.B., J.O.G., E.v.d.H., J.R.T., M.J., and T.P.S. wrote and edited the manuscript.

## Supplementary Material

giaa053_GIGA-D-19-00381_Original_Submission

giaa053_GIGA-D-19-00381_Revision_1

giaa053_Response_to_Reviewer_Comments_Original_Submission

giaa053_Reviewer_1_Report_Original_Submission Daniel Read -- 11/28/2019 Reviewed

giaa053_Reviewer_2_Report_Original_Submission CÃ©dric Laczny -- 12/9/2019 Reviewed

giaa053_Reviewer_3_Report_Revision_1 Melody Clark -- 3/11/2020 Reviewed

giaa053_Supplemental_Files

## References

[1] Shade A, Carey CC, Kara E, et al. Can the black box be cracked? The augmentation of microbial ecology by high-resolution, automated sensing technologies. ISME J. 2009;3(8):881–88. 19458653 10.1038/ismej.2009.56

[2] Rodgers C A new approach to protecting ecosystems: The te awa tupua (Whanganui River Claims Settlement) Act 2017. Environ Law Rev. 2017;19(4):266–79.

[3] Davies SP, Jackson SK The biological condition gradient: a descriptive model for interpreting change in aquatic ecosystems. Ecol Appl. 2006;16(4):1251–66. 16937795 10.1890/1051-0761(2006)016[1251:tbcgad]2.0.co;2

[4] Bramblett RG, Fausch KD Variable fish communities and the index of biotic integrity in a western great plains river. Trans Am Fish Soc. 1991;120(6):752–69.

[5] Karr JR Assessment of biotic integrity using fish communities. Fisheries. 1981;6(6):21–7.

[6] Murray AK, Zhang L, Yin X, et al. Novel insights into selection for antibiotic resistance in complex microbial communities. mBio. 2018;9(4), doi:10.1128/mBio.00969-18. PMC6058293 30042197

[7] Brown BL, LePrell RV, Franklin RB, et al. Metagenomic analysis of planktonic microbial consortia from a non-tidal urban-impacted segment of James River. Stand Genom Sci. 2015;10:65. 10.1186/s40793-015-0062-5 PMC4575436 26388969

[8] Staley C, Gould TJ, Wang P, et al. Core functional traits of bacterial communities in the upper Mississippi River show limited variation in response to land cover. Front Microbiol. 2014;5:414. 25152748 10.3389/fmicb.2014.00414 PMC4126211

[9] Staley C, Unno T, Gould TJ, et al. Application of Illumina next-generation sequencing to characterize the bacterial community of the upper Mississippi River. J Appl Microbiol. 2013;115(5):1147–58. 23924231 10.1111/jam.12323

[10] Menegon M, Cantaloni C, Rodriguez-Prieto A, et al. On site DNA barcoding by nanopore sequencing. PLoS One. 2017;12(10):e0184741. 28977016 10.1371/journal.pone.0184741 PMC5627904

[11] Johnson JS, Spakowicz DJ, Hong B-Y, et al. Evaluation of 16S rRNA gene sequencing for species and strain-level microbiome analysis. Nat Commun. 2019;10:5029. 31695033 10.1038/s41467-019-13036-1 PMC6834636

[12] Brown BL, Watson M, Minot SS, et al. MinION^TM^ nanopore sequencing of environmental metagenomes: a synthetic approach. Gigascience. 2017;6(3):1–10. 10.1093/gigascience/gix007 PMC5467020 28327976

[13] Nicholls SM, Quick JC, Tang S, et al. Ultra-deep, long-read nanowire sequencing of mock microbial community standards. Gigascience. 2019;8(5), doi:10.1093/gigascience/giz043. PMC6520541 31089679

[14] Pearman W, Freed N, Silander O The advantages and disadvantages of short- and long-read metagenomics to infer bacterial and eukaryotic community composition. bioRxiv. 2019, doi:10.1101/650788.

[15] Dilthey AT, Jain C, Koren S, et al. Strain-level metagenomic assignment and compositional estimation for long reads with MetaMaps. Nat Commun. 2019;10:3066. 31296857 10.1038/s41467-019-10934-2 PMC6624308

[16] White RA III, Bottos EM, Chowdhury RT, et al. Moleculo long-read sequencing facilitates assembly and genomic binning from complex soil metagenomes. mSystems. 2016;1:e00045–16. 27822530 10.1128/mSystems.00045-16 PMC5069762

[17] Bertrand D, Shaw J, Kalathiyappan M, et al. Hybrid metagenomic assembly enables high-resolution analysis of resistance determinants and mobile elements in human microbiomes. Nat Biotechnol. 2019;37:937–44. 31359005 10.1038/s41587-019-0191-2

[18] Ghai R, Rodriguez-Valera F, McMahon KD, et al. Metagenomics of the water column in the pristine upper course of the Amazon River. PLoS One. 2011;6(8):e23785. 21915244 10.1371/journal.pone.0023785 PMC3158796

[19] Holben WE GC fractionation allows comparative total microbial community analysis, enhances diversity assessment, and facilitates detection of minority populations of bacteria, de Bruijn FJ, ed. In: Handbook of Molecular Microbial Ecology I: Metagenomics and Complementary Approaches. New York: Wiley; 2011: 183–96.

[20] Oh S, Caro-Quintero A, Tsementzi D, et al. Metagenomic insights into the evolution, function, and complexity of the planktonic microbial community of Lake Lanier, a temperate freshwater ecosystem. Appl Environ Microbiol. 2011;77(17):6000–11. 21764968 10.1128/AEM.00107-11 PMC3165412

[21] Van Rossum T, Peabody MA, Uyaguari-Diaz MI, et al. Year-long metagenomic study of river microbiomes across land use and water quality. Front Microbiol. 2015;6:1405. 26733955 10.3389/fmicb.2015.01405 PMC4681185

[22] Hamner S, Brown BL, Hasan NA, et al. Metagenomic profiling of microbial pathogens in the Little Bighorn River, Montana. Int J Environ Res Public Health. 2019;16(7):1097. 10.3390/ijerph16071097 PMC6479903 30934749

[23] Newton RJ, Jones SE, Eiler A, et al. A guide to the natural history of freshwater lake bacteria. Microbiol Mol Biol Rev. 2011;75(1):14–49. 21372319 10.1128/MMBR.00028-10 PMC3063352

[24] Azam F, Fenchel T, Field JG, et al. The ecological role of water-column microbes in the sea. Mar Ecol Prog Ser. 1983;10:257.

[25] Kirchman DL, Dittel AI, Findlay SEG, et al. Changes in bacterial activity and community structure in response to dissolved organic matter in the Hudson River, New York. Aquat Microb Ecol. 2004;35:243–57.

[26] Pernthaler J Freshwater microbial communities. In: Rosenberg E, DeLong EF, Lory S, E Stackebrandt, Thompson F, eds. The Prokaryotes: Prokaryotic Communities and Ecophysiology. Berlin, Heidelberg; Springer; 2013: 97–112.

[27] Winter C, Hein T, Kavka G, et al. Longitudinal changes in the bacterial community composition of the Danube River: A whole-river approach. Appl Environ Microbiol. 2007;73(2):421–31. 17085708 10.1128/AEM.01849-06 PMC1796958

[28] Junger PC, Amado AM, Paranhos R, et al. Salinity drives the virioplankton abundance but not production in tropical coastal lagoons. Microb Ecol. 2018;75(1):52–63. 28721503 10.1007/s00248-017-1038-3

[29] Biller SJ, Berube PM, Dooley K, et al. Marine microbial metagenomes sampled across space and time. Sci Data. 2018;5:180176. 30179232 10.1038/sdata.2018.176 PMC6122167

[30] Sunagawa S, Coelho LP, Chaffron S, et al. Structure and function of the global ocean microbiome. Science. 2015;348:1261359. 25999513 10.1126/science.1261359

[31] Beaulaurier J, Luo E, Eppley J, et al. Assembly-free single-molecule sequencing recovers complete virus genomes from natural microbial communities, Genome Res. 2020, doi:10.1101/gr.251686.119. PMC7111524 32075851

[32] Amos GCA, Hawkey PM, Gaze WH, et al. Waste water effluent contributes to the dissemination of CTX-M-15 in the natural environment. J Antimicrob Chemother. 2014;69(7):1785–91. 24797064 10.1093/jac/dku079 PMC4054988

[33] Kristiansson E, Fick J, Janzon A, et al. Pyrosequencing of antibiotic-contaminated river sediments reveals high levels of resistance and gene transfer elements. PLoS One. 2011;6(2):e17038. 21359229 10.1371/journal.pone.0017038 PMC3040208

[34] Su J-Q, Yu X, Yao H-Y, et al. Metagenomic assembly unravel microbial response to redox fluctuation in acid sulfate soil. Soil Biol Biochem. 2017;105:244.

[35] Deiner K, Fronhofer EA, Mächler E, et al. Environmental DNA reveals that rivers are conveyer belts of biodiversity information. Nat Commun. 2016;7:12544. 27572523 10.1038/ncomms12544 PMC5013555

[36] Gweon HS, Shaw LP, Swann J, et al. The impact of sequencing depth on the inferred taxonomic composition and AMR gene content of metagenomic samples. Environ Microbiome. 2019;14:7. 10.1186/s40793-019-0347-1 PMC8204541 33902704

[37] Vaz-Moreira I, Nunes OC, Manaia CM Bacterial diversity and antibiotic resistance in water habitats: searching the links with the human microbiome. FEMS Microbiol Rev. 2014;38(4):761–78. 24484530 10.1111/1574-6976.12062

[38] http://ville.montreal.qc.ca/portal/page?_pageid=7237,75397570&_dad=portal&_schema=PORTAL.Interactive map for monitoring the bacteriological quality of watercourses in Montreal. 10 August 2019.

[39] Reddington K, Eccles D, O'Grady J, et al. DNA extraction and purification for MARC phase 3 global river water sequencing. Protocols.io, 2019 10.17504/protocols.io.qtgdwjw

[40] Yuan S, Cohen DB, Ravel J, Abdo Z, Forney LJ Evaluation of methods for the extraction and purification of DNA from the human microbiome. PLoS ONE. 2012;7(3):e33865. 22457796 10.1371/journal.pone.0033865 PMC3311548

[41] Wick R Porechop. https://github.com/rrwick/Porechop. Accessed 1 August 2019.

[42] gringer Eccles D Gringer/bioinfscripts: Tree Lab / Global River Release. Zenodo. 2019, doi:10.5281/zenodo.3240748.

[43] Wood DE, Salzberg SL Kraken: Ultrafast metagenomic sequence classification using exact alignments. Genome Biol. 2014;15(3):R46. 24580807 10.1186/gb-2014-15-3-r46 PMC4053813

[44] *Mockcommunity*. https://github.com/LomanLab/mockcommunity. Accessed 29 July 2019.

[45] Keegan KP, Glass EM, Meyer F MG-RAST, a metagenomics service for analysis of microbial community structure and function. Methods Mol Biol. 2016;1399:207–33. 26791506 10.1007/978-1-4939-3369-3_13

[46] Breitwieser FP, Salzberg SL Pavian: Interactive analysis of metagenomics data for microbiomics and pathogen identification. bioRxiv. 2016, doi:10.1101/084715. PMC8215911 31553437

[47] Eccles D MinION Global River Sequencing. CodeOcean 2019 10.24433/CO.6736538.v1.

[48] Reddington K, Eccles D, O'Grady J, et al. Supporting data for “Metagenomic analysis of planktonic riverine microbial consortia using nanopore sequencing reveals insight into river microbe taxonomy and function.”. GigaScience Database. 2020 10.5524/100725. PMC7285869 32520351

