# Corrigendum to: Metagenomic analysis of planktonic riverine microbial consortia using nanopore sequencing reveals insight into river microbe taxonomy and function

**DOI:** 10.1093/gigascience/giaa074

**Published:** 2020-06-24

**Authors:** Kate Reddington, David Eccles, Justin O'Grady, Devin M Drown, Lars Hestbjerg Hansen, Tue Kjærgaard Nielsen, Anne-Lise Ducluzeau, Richard M Leggett, Darren Heavens, Ned Peel, Terrance P Snutch, Anthony Bayega, Spyridon Oikonomopoulos, Jiannis Ragoussis, Thomas Barry, Eric van der Helm, Dino Jolic, Hollian Richardson, Hans Jansen, John R Tyson, Miten Jain, Bonnie L Brown

**Affiliations:** Microbial Diagnostics Research Laboratory, Microbiology, School of Natural Sciences, National University of Ireland, University Road, Galway, Ireland H91 TK33, Ireland; Malaghan Institute of Medical Research, Gate 7, Victoria University Kelburn Parade, Wellington 6140, Wellington 6242, New Zealand; Quadram Institute Bioscience, Norwich Research Park, Norwich NR4 7UQ, UK; Norwich Medical School, University of East Anglia, James Watson Rd, Norwich NR4 7TJ, UK; Department of Biology and Wildlife, Institute of Arctic Biology, University of Alaska Fairbanks, 2140 Koyukuk Drive, Fairbanks, AK 9975-7000, USA; Department of Environmental Science, Aarhus University, PO Box 358, Frederiksborgvej 399, DK-4000 Roskilde, Denmark; Department of Plant and Environmental Sciences, University of Copenhagen, Thorvaldsensvej 40, 1871 Frederiksberg C, Denmark; Department of Environmental Science, Aarhus University, PO Box 358, Frederiksborgvej 399, DK-4000 Roskilde, Denmark; Department of Plant and Environmental Sciences, University of Copenhagen, Thorvaldsensvej 40, 1871 Frederiksberg C, Denmark; Institute of Arctic Biology, University of Alaska Fairbanks, 311 Irving 1 Building P.O. Box 757000 2140 Koyukuk Drive Fairbanks, AK 99775-7000, USA; Earlham Institute, Norwich Research Park, Norwich NR4 7UQ, UK; Earlham Institute, Norwich Research Park, Norwich NR4 7UQ, UK; Earlham Institute, Norwich Research Park, Norwich NR4 7UQ, UK; Michael Smith Laboratories and Department of Zoology, University of British Columbia, #301-2185 East Mall Vancouver, BC V6T 1Z4, Canada; McGill University and Genome Quebec Innovation Centre, Department of Human Genetics, McGill University, 3640 rue University, Montreal, Quebec H3A 0C7, Canada; McGill University and Genome Quebec Innovation Centre, Department of Human Genetics, McGill University, 3640 rue University, Montreal, Quebec H3A 0C7, Canada; McGill University and Genome Quebec Innovation Centre, Department of Human Genetics, McGill University, 3640 rue University, Montreal, Quebec H3A 0C7, Canada; Nucleic Acid Diagnostics Research Laboratory, Microbiology, School of Natural Sciences, National University of Ireland, University Road, Galway, Ireland H91 TK33, Ireland; Novo Nordisk Foundation Center for Biosustainability, Technical University of Denmark, Building 220, Kemitorvet, 2800 Kgs. Lyngby, Denmark; Department for Evolutionary Biology, Max Planck Institute for Developmental Biology, Max-Planck-Ring 5 72076 Tubingen, Germany; Norwich Medical School, University of East Anglia, James Watson Rd, Norwich NR4 7TJ, UK; Future Genomics Technologies B.V., Nucleus building, Sylviusweg 74, 2333 BE Leiden, The Netherlands; Michael Smith Laboratories and Department of Zoology, University of British Columbia, #301-2185 East Mall Vancouver, BC V6T 1Z4, Canada; UC Santa Cruz Genomics Institute, 1156 High Street, Santa Cruz, CA 95064, USA; Department of Biological Sciences, University of New Hampshire, 38 Academic Way, Durham, NH 03824, USA

In the original publication of this article, the author Jiannis Ragoussis was not part of the author byline. His name, affiliation, and funding information have been added to this article. The authors regret this error.

