# Supplementary material for: Metagenomic analysis of planktonic riverine microbial consortia using nanopore sequencing reveals insight into river microbe taxonomy and function
Source: Gigascience. 2020 Jun 10;9(6):giaa053. doi: 10.1093/gigascience/giaa053 (PMC7285869; doi:10.1093/gigascience/giaa053)
Supplement: giaa053_GIGA-D-19-00381_Original_Submission [file giaa053_giga-d-19-00381_original_submission.pdf]

GigaScience

Metagenomic analysis of planktonic riverine microbial consortia using nanopore sequencing reveals urban influence on river ecology

--Manuscript Draft--

**Manuscript Number:**	GIGA-D-19-00381
**Full Title:**	Metagenomic analysis of planktonic riverine microbial consortia using nanopore sequencing reveals urban influence on river ecology
**Article Type:**	Research

Funding Information:

National Institute of General Medical Sciences (UL1GM118991, TL4GM118992, and RL5GM118990)	Dr Devin M. Drown<br>Dr Anne-Lise Ducluzeau
Alaska INBRE (P20GM103395)	Dr Devin M. Drown<br>Dr Anne-Lise Ducluzeau
AUFF- NOVA (AUFF-E-201 7-9-38)	Dr Lars Hestbjerg Hansen<br>Dr Tue Kjærgaard Nielsen
Biotechnology and Biological Sciences Research Council (BB/J004669/1 and BB/CSP17270/1)	Dr Richard M. Leggett<br>Dr Darren Heavens
Biotechnology and Biological Sciences Research Council (BB/M011216/1)	Dr Ned Peel
Genome Canada (CA)	Dr John R. Tyson
Canada Foundation for Innovation (32557)	Dr John R. Tyson
Compute Canada (WST-164-AB)	Dr John R. Tyson
national science foundation division of environmental biology (1355059)	Dr Bonnie L Brown
Canada Research Chair in Biotechnology and Genomics-Neurobiology	Dr Terrance P. Snutch
Canadian Institutes of Health Research (10677)	Dr Terrance P. Snutch
Koerner Foundation (NA)	Dr Terrance P. Snutch

Abstract:

Riverine ecosystems are biogeochemical powerhouses driven largely by microbial communities that inhabit water columns and sediments. To gain a broader understanding of the ecological and anthropogenic implications of river microbial consortia, we investigated the metagenomes of eleven rivers across three continents using MinION nanopore sequencing, a portable platform that could be useful for future global river monitoring. Up to 10 Gb of data per run were generated with average read lengths of 3.4 kb. Diversity and diagnosis of river function potential was accomplished with 0.5-1.0×106 long reads. Our observations for seven of the eleven rivers conformed to previous findings and we exposed previously unrecognized microbial biodiversity in the other four rivers. Deeper understanding that emerged is that river microbial consortia and the ecological functions they fulfil did not align with geographic location, but instead implicated ecological responses to urban and other anthropogenic effects, and that changes in taxa manifested over a very short geographic space.

**Corresponding Author:**	Bonnie L Brown<br>Virginia Commonwealth University<br>Durham, NH UNITED STATES
**Corresponding Author Secondary Information:**	
**Corresponding Author's Institution:**	Virginia Commonwealth University
**Corresponding Author's Secondary**	

Powered by Editorial Manager® and ProduXion Manager® from Aries Systems Corporation

**Institution:**	
**First Author:**	Bonnie L Brown
**First Author Secondary Information:**	
**Order of Authors:**	Bonnie L Brown
	Kate Reddington
	David Eccles
	Justin O'Grady
	Devin M. Drown
	Lars Hestbjerg Hansen
	Tue Kjærgaard Nielsen
	Anne-Lise Ducluzeau
	Richard M. Leggett
	Darren Heavens
	Ned Peel
	Terrance P. Snutch
	Anthony Bayega
	Spyridon Oikonomopoulos
	Ioannis Ragoussis
	Thomas Barry
	Eric van der Helm
	Dino Jolic
	Hollian Richardson
	John R. Tyson
	Miten Jain
	Hans Jansen
**Order of Authors Secondary Information:**	
**Additional Information:**	
**Question**	**Response**
Are you submitting this manuscript to a special series or article collection?	No
**Experimental design and statistics**<br><br>Full details of the experimental design and statistical methods used should be given in the Methods section, as detailed in our Minimum Standards Reporting Checklist. Information essential to interpreting the data presented should be made available in the figure legends.	Yes

Have you included all the information requested in your manuscript?	
**Resources**<br><br>A description of all resources used, including antibodies, cell lines, animals and software tools, with enough information to allow them to be uniquely identified, should be included in the Methods section. Authors are strongly encouraged to cite Research Resource Identifiers (RRIDs) for antibodies, model organisms and tools, where possible.<br><br>Have you included the information requested as detailed in our Minimum Standards Reporting Checklist?	Yes
**Availability of data and materials**<br><br>All datasets and code on which the conclusions of the paper rely must be either included in your submission or deposited in publicly available repositories (where available and ethically appropriate), referencing such data using a unique identifier in the references and in the “Availability of Data and Materials” section of your manuscript.<br><br>Have you have met the above requirement as detailed in our Minimum Standards Reporting Checklist?	Yes

Click here to download Manuscript Metagenomic analysis of planktonic 24x19 for GigaScience.docx

Metagenomic analysis of planktonic riverine microbial consortia using nanopore sequencing reveals urban influence on river ecology

Kate Reddington[1*], David Eccles[2*], Justin O'Grady[3,4*], Devin M. Drown[5*], Lars Hestbjerg Hansen[6,7], Tue Kjærgaard Nielsen[6,7], Anne-Lise Ducluzeau[8], Richard M. Leggett[9], Darren Heavens[9], Ned Peel[9], Terrance P. Snutch[10], Anthony Bayega[11], Spyridon Oikonomopoulos[11], Ioannis Ragoussis[11], Thomas Barry[12], Eric van der Helm[13], Dino Jolic[14], Hollian Richardson[4], Hans Jansen[15*], John R. Tyson[10*], Miten Jain[16*], Bonnie L. Brown[17*]

[1] Microbial Diagnostics Research Laboratory, Microbiology, School of Natural Sciences, National University of Ireland, Galway, Ireland
[2] Malaghan Institute of Medical Research, Newtown, Wellington 6242, New Zealand
[3] Quadram Institute Bioscience, Norwich Research Park, Norwich, UK NR4 7UQ.
[4] Norwich Medical School, University of East Anglia, Norwich, UK NR4 7TJ.
[5] Department of Biology and Wildlife, Institute of Arctic Biology, University of Alaska Fairbanks, Fairbanks, AK, USA
[6] Department of Environmental Science, Aarhus University, Frederiksborgvej 399, Roskilde, Denmark
[7] Department of Plant and Environmental Sciences, University of Copenhagen, Thorvaldsensvej 40, Frederiksberg, Denmark
[8] Institute of Arctic Biology, University of Alaska Fairbanks, Fairbanks, AK, USA
[9] Earlham Institute, Norwich Research Park, Norwich, UK
[10] Michael Smith Laboratories and Department of Zoology, University of British Columbia, Vancouver, BC Canada V6T 1Z4
[11] McGill University and Genome Quebec Innovation Centre, Department of Human Genetics, McGill University, Montreal, Canada
[12] Nucleic Acid Diagnostics Research Laboratory, Microbiology, School of Natural Sciences, National University of Ireland, Galway, Ireland
[13] Novo Nordisk Foundation Center for Biosustainability, Technical University of Denmark, Lyngby 2800, Denmark
[14] Department for Evolutionary Biology, Max Planck Institute for Developmental Biology, 72076 Tubingen, Germany
[15] Future Genomics Technologies B.V., Leiden, the Netherlands
[16] UC Santa Cruz Genomics Institute, Santa Cruz, CA 95064, USA
[17] University of New Hampshire, Dept. of Biological Sciences, 38 Academic Way, Durham, NH USA 03824
* Equal contributors and anchors
Anchor author emails:

- Kate Reddington: kate.reddington@nuigalway.ie
- David Eccles: bioinformatics@gringene.org
- Justin O'Grady: Justin.OGrady@uea.ac.uk
- Devin M. Drown: dmdrown@alaska.edu
- Hans Jansen: jansen@futuregenomics.tech
- John R. Tyson: jtyson@msl.ubc.ca
- Miten Jain: miten@soe.ucsc.edu
- Bonnie L. Brown: bonnie.brown@unh.edu (contact author)

Keywords: temperate river metagenomes, MinION, long read, nanopore sequencing

Abstract

Riverine ecosystems are biogeochemical powerhouses driven largely by microbial communities that inhabit water columns and sediments. To gain a broader understanding of the ecological and anthropogenic implications of river microbial consortia, we investigated the metagenomes of eleven rivers across three continents using MinION nanopore sequencing, a portable platform that could be useful for future global river monitoring. Up to 10 Gb of data per run were generated with average read lengths of 3.4 kb. Diversity and diagnosis of river function potential was accomplished with $0.5\text{-}1.0\times10^6$ long reads. Our observations for seven of the eleven rivers conformed to previous findings and we exposed previously unrecognized microbial biodiversity in the other four rivers. Deeper understanding that emerged is that river microbial consortia and the ecological functions they fulfil did not align with geographic location, but instead implicated ecological responses to urban and other anthropogenic effects, and that changes in taxa manifested over a very short geographic space.

Background

River ecosystems are Earth's biogeochemical powerhouses, and riverine processes largely are driven by the microbial communities that inhabit their water columns and sediments (Shade et al. 2009). From an applied anthropogenic perspective, rivers are the life-blood of human communities; recognition of this perspective led the New Zealand Government to grant legal personhood status to the Whanganui River as an indivisible and living whole (Rodgers 2017). Rivers provide food, drinking water, and are a resource for agricultural and industrial use coupled with waste distribution, thereby reflecting a fingerprint of the total environment. Frequently, these

services and activities are provided within an alarming proximity to each other. Regulatory authorities in many regions currently assess river "health" for management and monitoring of water resources using methods such as Biological Condition Gradient (Davies and Jackson 2006) and Index of Biotic Integrity (Karr 1981; Bramblett and Fausch 1991). Such assessments score river "health" based on occurrence of certain conditions, response to stress, and abundance of eukaryotic organisms. The recent focus on antimicrobial resistance (AMR) has highlighted the potential of AMR genes in aquatic microbes as a potential threat to human health. Complex microbial river water communities, often contributed to by human and animal activity, have more AMR genes than simple communities (Murray et al. 2018). However, it is unclear at the moment which microbial resistance genes (or gene combinations) are a threat to human health and at what concentrations. Given that high-throughput sequencing is becoming economically viable for environmental monitoring, it is now possible to accurately characterize river metagenomes and determine the extent of variability among them. We can utilize this technology to monitor the levels of water-borne disease microorganisms and AMR genes and we are gaining an understanding that microbial community data should be included in broad-scale ecosystem models.

Data Description

It is likely that there is a correlation between river water microbial community composition, as determined by metagenomic sequencing, and river function and health. Recent eco-genomic methods offer the capability to understand river ecosystems in greater detail, but for this approach to be widely utilized, field deployable sequencing technology is necessary. We designed a study to evaluate river water metagenomes and the occurrence of riverine xenobiotic components, on a global scale, using the MinION portable sequencer paired with rapid data analysis. Assigning

taxonomy and/or function for complex environmental samples such as rivers traditionally has been accomplished using whole genome short-read sequences or 16S amplicon sequences (Staley et al. 2014; Brown et al. 2015), and only recently has been investigated using long reads (Brown et al. 2017). Such long-read data could enhance our knowledge of ecosystem functions coupled to microbial community structure and likely more accurately model the biogeochemical processes driven by microbes. A deeper understanding of microbial diversity is needed to discern the implications on human health (Bertrand et al. 2019), e.g., the occurrence of antibiotic-resistant strains of bacteria in waterways that provide food and drinking water, and on productivity (e.g., nutrient cycling, crop irrigation, disposal of industrial and sanitation-related waste). To evaluate proof-of-concept, we sampled a diverse set of 11 contrasting rivers and waterways across the globe, and we describe here a basic, high-level analysis of the results using multiple bioinformatic pipelines, providing all of the underlying raw sequence data for additional discovery and analysis by other researchers. We document the potential of long-read nanopore sequencing and real-time analysis of DNA obtained globally for environmental monitoring of the river biota, detecting pathogen and AMR presence and diversity, with the aim of ultimately enabling water quality enhancement. We further believe that the methodology developed in this study provides a robust, small footprint protocol that will facilitate broadening riverine metagenomic studies.

Analyses

Length and count statistics

Libraries constructed by the MinION SQK-RLB001 kit consistently produced 2-5 kbp fragments (Figure 1 B&D) that yielded sequencing data sets averaging $1.1 \cdot 10^6$ reads ($3.8 \cdot 10^9$ bases) of length $3.4 \cdot 10^3$ bp (Figure 1 A&C, Table 2). Metagenomic results rarefaction (Figure 2) indicated that 0.5-$1.0 \cdot 10^6$ sampled reads were adequate to capture the OTU diversity of most samples.

Taxonomic diversity

Using One Codex for metagenomic assignment of the WGS long-read data, a large number of reads were not classified, possibly a result of the low number of eukaryotic and viral genomes in the database. After preliminary analysis of the results generated, One Codex was removed from the pipeline in favour of MG-RAST and Kraken2. Nearly all of the river metagenomes exhibited multimodal GC distributions, an indication of multiple domain representation; this mirrors the GC representation in many other freshwater environmental metagenomes (Ghai et al. 2011; Staley et al. 2014; Holben 2011; Oh et al. 2011). Reads for most river metagenomes were overwhelmingly assigned to the Bacteria domain at ≥94% with one exception, Sydhavnen at Copenhagen Harbor, where Virus accounted for >25% of the data and Bacteria only 68%. Eukaryotes were identified by MG-RAST in every metagenome at a level of ≤4% of reads, and Archaea were represented in all metagenomes by 0.2-6.0% of reads.

The five most common bacterial phyla observed were Proteobacteria, Bacteroidetes, Actinobacteria, Firmicutes, and Cyanobacteria. Proteobacteria were the most abundant prokaryote in most metagenomes (Vedder Canal was a distinct outlier where Bacteroidetes predominated) and within that group, the predominant taxon was the Burkholderiales, dominated therein by the Comamonadaceae comprised predominantly of *Acidovorax* species (0.3-5% of assigned bacterial reads; iron and uranium oxidizers, nitrotolulene degraders, and plant pathogens) and *Polaromonas* (0.1-4% of bacterial reads; degraders of chlorinated-alkenes and naphthalene). Another group that dominated the prokaryote hits was Bacteroidetes, composed overwhelmingly of *Flavobacterium* (0.5-35% of bacteria reads; extremely common in soils and freshwaters, and some are known disease agents). Moderately abundant prokaryotes were Actinobacteria, consisting nearly

completely of Actinomycetales, fungus-like soil bacteria (0.4-41% of bacteria). A final group of prokaryotes, Archaea, occurred at an average of 1% of read assignments in all metagenomes except Chena River, which contained a high proportion of Archaea, 6%. Other published river metagenome studies recorded Archaea at the 1% level (C. Staley et al. 2013; Staley et al. 2014; Van Rossum et al. 2015; Brown et al. 2015). Archaebacterial groups detected were extremely similar across most metagenomes (most of which were Methanomicrobia, CO_2 reducers); a notable exception was the metagenome for Sydhavnen (Copenhagen Harbour), where most of this group's representatives were instead Thaumarchaeota (noted for the ability to nitrify via oxidizing ammonia aerobically), dominated by *Nitrosopumilus*, a common promulgator of the marine nitrogen cycle.

Alpha diversity across the 13 samples ranged from a low of 135 species (Vedder Canal) to a high of 1139 species (Chena River). Metagenomes of the typical temperate urban rivers Yare, Rhine, Neckar, Corrib, James, and St. Laurent had average alpha diversity of 413+- 29 SE, and exhibited family sets that conformed to the core groups that have been found to dominate other large temperate rivers and lakes (Newton et al. 2011; Staley et al. 2013; Staley et al. 2014; Brown et al. 2015, Hamner et al. 2019), and the genera observed concurred with what is expected based on a general understanding of river ecology (covered more extensively below). Like those "typical" rivers, the metagenomes of Vedder River and Canal exhibited prokaryote families ubiquitous in soils and water environments, but these two samples stood apart due to higher abundance of Cytophagaceae and Burkholderiaceae and lower abundance of Streptomycetaceae than in the other rivers. Metagenomes of Chena and Karori Rivers also exhibited prokaryote families ubiquitous in soils and water environments, but their consortia were dominated by different families than the other rivers. We also saw that Chena River showed evidence of hydrocarbon influence as the 4th,

8th, and 10th most abundant microbe families are important degraders of methylnaphthalene and BTEX (benzene, toluene, ethylbenzene and xylene). Karori River was distinctive in that some of its most numerous microbial families were either marine (Cytophagaceae, Alteromonadaceae, and Vibrionaceae) or signified the presence of sewage (Enterobacteriaceae and Campylobacteraceae). The Skævinge waste water inlet metagenome was unique, as expected, in that it was dominated by families (5 of the top 10) that are linked to sewage. The Sydhavnen metagenome was unique, as expected, due to abundance of marine bacteria, marine-related viruses, and algae; only two of the major prokaryote families were typical of freshwater river ecosystems.

Of 1249 genera classified, 69 occurred at 1% or greater in any one of the 13 metagenomic samples and of those, 35 genera were represented on average at 1% or greater in all of the samples (Table 3). For the majority of samples, the most common OTUs were the bacterial genera *Flavobacterium*, *Polynucleobacter*, *Acidovorax*, *Polaromonas*, and *Streptomyces*. These microbes, known to be members of the "microbial loop" (Azam et al. 1983), are among the predominant drivers of water and soil ecosystem processes and have been documented as major contributors to the consortia of other aquatic systems (Kirchman et al. 2004; Winter et al. 2007; Newton et al. 2011; Ghai et al. 2011; Pernthaler 2013; C. Staley et al. 2013; Brown et al. 2015; Hamner et al. 2019). Three rivers exhibited very low frequencies of the common river OTUs. These exceptions included Chena (where *Clostridium*, *Bacillus*, and *Geobacter* predominated), Vedder (where *Pelagibacter* and Ricketsiales were most common), and Karori Stream (where most numerous were *Cellvibrio*, *Pseudomonas*, *Arcobacter*, *Bacteroides*, and *Burkholderia*). The least typical "river" samples were the wastewater influent at Skævinge (where the dominant genus was *Arcobacter*, 48.7%, followed by *Bacteroides* and *Campylobacter*, both of which are significant clinical pathogens) and Sydhavnen at Copenhagen Harbor (dominated by Prasinovirus and

Phycoviridae, and having primary bacterial genera *Flavobacterium* and *Candidatus Pelagibacter*). Across all metagenomes, five significant pathogens were detected at >=1%. Present in all 13 metagenomes were *Campylobacter* (normalized proportion of 0.1-2.9%), *Clostridium* (0.3-3.2%), and *Prevotella* (0.2-1.3%). *Corynebacter* was in all except Karori Stream (0.1-1.6%) and *Helicobacter* present in all except Yare W and Neckar (0.1-1.5%). The fact that the taxonomic assignments for most rivers also implicated taxa that are anthropogenically-relevant such as xenobiotic processors, disease-causing organisms, and pathogens of humans, fish, and crops is not novel. Xenobiotics and significant pathogens previously were observed for one of the rivers examined in this study (James River, (Brown et al. 2015)) and have been documented using WGS data for other river metagenomes (C. Staley et al. 2013; Hamner et al. 2019).

The long read WGS data provided important novel insight into the viral complements of some river metagenomes. Across the 11 rivers (13 sampling sites), the normalized proportions of viral reads ranged from 0.03-25.8% of read assignments. The most common observation for 11 samples (virus accounting for <1% of reads) is typical of other river planktonic metagenomes (Ghai et al. 2011; C. Staley et al. 2013; Staley et al. 2014; Van Rossum et al. 2015; Brown et al. 2015). Except for the one metagenome outlier, most virus read annotations were similar to T4-like virus (bacteriophages with some similarity to cloning vectors). The next most common were Phycoviridae (types that infect bacteria and archaea), followed by Iridoviridae (insect virus), and *Cafeteria roenbergensis* virus (CroV; a giant virus of marine phagotrophic flagellates). The notable outlier metagenome was Sydhavnen (Copenhagen Harbour), where more than 25% of all reads were virus. These were not the type observed to dominate the ther river metagenomes; instead the dominant types were Prasinovirus (52,116 annotations, observed e-values >= $1\times e^{-9}$, alignment lengths >= 38, identity >= 80.8%) and Phycodnavirus (e-values >= $1\times e^{-7}$, alignment

lengths all >34, all showed >72% identity), which infect oceanic picoalgae - *Bathycoccus*, *Ostreococcus*, *Micromonas* (family Mamiellaceae) and other common groups of coastal green algae and cyanobacteria. Similar viral annotations were found in other samples, but at 5-10 times lower abundance. Capture of this viral event may reflect the effect of oceanic water mixing with fresher water as salinity can influence the rate of viral decay and others have observed that algae transitioning from fresher to more saline waters experience increased viral abundances (Junger et al. 2018). Alternatively, there could have been a recent bloom of picoalgae that advected onshore and was at the time of sampling in decline. The detection of such a high proportion of viral reads is notable in comparison to other halophilic WGS metagenomes where viruses generally are detected at <= 2% (Biller et al. 2018, Sunagawa et al. 2018), but actually has been seen recently as a significant benefit of the MinION sequencing method (Beaulaurier et al., 2019).

Despite the intentional methodological focus on picoplankton, a wide variety of eukaryotes (average 2% of read assignments) contributed to the river metagenomes. The same core phyla were detected across all samples, differing in proportion, and were highly similar to the taxa identified in other published riverine metagenomes (Ghai et al. 2011; C. Staley et al. 2013; Staley et al. 2014; Van Rossum et al. 2015; Brown et al. 2015). Groups represented by ≥1% read assignments included Protists of various types (15%: amoebae, flagellates, ciliates), Ascomycota (12%: fungi), Chordata (12%: rodents and insectivores were predominant, followed by amphibian, fishes, and birds), Streptophyta (11%: predominantly castor, *Populus*, *Arabidopsis*, grape, followed by wheat, rice, corn, and mosses), Chlorophyta (10%: nearly all *Volvox* and *Chlamydomonas,* except for Sydhavnen where the predominant hits were marine prasinophytes), Cnidaria (10%: roughly equally split between Anthozoa and Hydrozoa, freshwater hydroids), Arthropoda (6%: nearly all hits were insects followed by spiders), Bacillariophyta (5%: diatoms), Apicomplexa (5%: nearly

all parasitic), Nematoda (4%: equally split between free living nematodes and parasitic filarial roundworms), and Basidiomycota (3%: in decreasing order, mushrooms, yeasts, smuts, and galls). In many cases, these observed taxa were telling of upstream agricultural and urban effects as has been observed in other river metagenomes (C. Staley et al. 2013; Staley et al. 2014; Brown et al. 2015).

Representative PCAs created from both Kraken2 results based on the annotated families imputed with missMDA (not shown) and the MG-RAST normalized family frequencies, clustered River Yare samples collected east and west of Norwich, suggesting that they have similar metagenomic profiles (Figure 3 A&B). Conversely, geographically proximal Vedder River and Vedder Canal metagenomes did not cluster, indicating that although these are within the same tributary, the highly different anthropogenic effects on the waterways affects the river microbial consortia. River location by longitude, latitude, country, or continent was not reflected in the PCA grouping. The fact that both family and function PCAs yielded similar groupings and that those clusters did not reflect geography, leads to the conclusion that the consortia and the ecological functions they fulfil may be more important than a river's precise location.

Functional diversity

There were 2889 COG pathways and 249 KO pathways annotated across the 13 samples. The distribution of detected functions *versus* sequence count was logarithmic (Figure 4) indicating that approximately $2.5 \cdot 10^5$ long reads appear necessary to adequately diagnose river function potential using the MinION sequencing platform, a range well within the read output for 9 out of 12 experiments in this study. The long read data yielded assignments for functions of Bacteria comparable to North American river bacterial functions detected using data from other sequencing

platforms (C. Staley et al. 2013; Staley et al. 2014; Brown et al. 2015), the vast majority of which were associated with basic cellular housekeeping (Table 4).

Prior studies suggest that waste water release contributes to river resistomes (Amos et al. 2014; Kristiansson et al. 2011; Su et al. 2017; Hamner et al. 2019) and as mentioned above, we found signals of urban sewage in all of the metagenomes examined, at low abundance in most but unexpectedly high in others. We also detected other functions that indicated how these river consortia respond to the anthropogenic influences on these waterways (Table 5). For example, across the river metagenomes, 24 different mechanisms (0.8% of the COG processes detected) were related to antibiotic or multidrug resistance, toxins, or virulence. The most prevalent of those were AMR pathways dominated by the cation/multidrug efflux pump and the ABC-type multidrug transport system (ATPase and permease components). According to the SEED viewer, 24 genes were detected that direct transporting and processing of heavy metals (As, Cu, Co, Zn, Pb, and Cd, those for Cu were highly represented). Of all KO functional pathways detected, 60 (24% of all annotated pathways) were related to processing of xenobiotic substances or to human or plant pathogens and diseases. The xenobiotic processes were dominated by degradation of benzoate [PATH:ko00362], chlorocyclohexane and chlorobenzene [PATH:ko00361], aminobenzoate [PATH:ko00627], nitrotoluene [PATH:ko00633], atrazine [PATH:ko00791], and dioxin [PATH:ko00621]. Similar observations were made for an earlier James River metagenome previously analyzed using different WGS sequencing technologies (Brown et al. 2015). The PCA analysis of Subsystem Functions (Figure 3) grouped samples in a nearly identical fashion as for the Family grouping giving support to the contention that microbial function is driving differences among river and waterway metagenomes, not location.

Negative Control Samples

The number of classified reads from the mapped negative control samples reads was very low in comparison to the number of river sample reads (~0.1%). Within all negative controls, 33 families were identified above a 1% proportion (in any control sample) and negative control reads accounted for 0.04±0.02% of the total read counts in the corresponding samples. Two of the negative control sample data sets had at most one read, and all but four sets had too few reads to be analyzed in MG-RAST. Ultimately, there was no obvious trend that indicated the source of negative control reads being a result of consistent sample contamination during sample preparation (i.e., the isolation, library prep, barcoding). Combined with the fact that the total number of negative reads were trivial in most cases and that there was no obvious pattern to their derivation, we performed no deeper investigation into the sources of negative control reads.

Discussion

Long read metagenome analysis for river taxonomy and function

Because rivers are used extensively for anthropogenic purposes (drinking water, recreation, agriculture, and industry), it is essential to understand how these activities affect the composition of river microbial consortia. Such understanding could be facilitated on a massive scale if there were a broadly applicable means for spatiotemporal river testing that could produce an unbiased representation of the microbial community. This would be especially helpful to document presence, distribution, and evolution of epidemiologically significant organisms. Nanopore technology is enabling the application of long-read nucleotide sequencing in a number of ecogenomic applications and holds promise for just such testing. We found that DNA sequence data from river water produced by the portable MinION device joined with metagenomic analysis provides a sensitive platform for investigating the diversity and ecological function of microbiota

inhabiting Earth's rivers and waterways. By sequencing DNA from rivers on three continents using MinION rapid sequencing and analyzing those data with both local and cloud-based tools, we obtained detailed results on taxonomy and function that implied just how distinct and ecologically responsive those river system microbiota are. Of the temperate urban rivers we investigated, 7 had average alpha diversity of 413+-29 SE species and were composed of family sets that conformed to the core groups that have been found to dominate other rivers previously studied using other sequencing platforms. An atypical observation was that we captured genomic signals of an algal/viral event in an eighth waterway sample (Sydnavnen).

In addition to the utility of this approach for studying river consortia, we present data that illustrate its potential for monitoring for anthropogenic effects on river biota, detecting pathogen presence and diversity throughout river systems, judging risk associated with water uses, and hopefully enhancing water quality. Our analyses exposed previously unrecognized aspects of microbial biodiversity in 4 waterways, where the metagenomes deviated from the expected suite of taxa; several illustrated marine influence, some showed taxa responsive to hydrocarbon pollution, and others had strong signals of taxa related to sewage and AMR gene matches linked to antibiotic resistance. We specifically observed that the most common OTUs (i.e., groups that occur at >= 1% of the detected consortia) are quite good indicators of the extent to which rivers and waterways are responding to anthropogenic impacts.

Additional bioinformatic development is necessary, however, to ultimately support a field-deployable sequencing device paired with deeply informative statistical analysis that has the capability of rapidly and comprehensively detecting microbes. Comparing widely available bioinformatic tools to analyze the river metagenomes, revealed that One Codex resulted in a very

high proportion of unclassified reads and, as the parameters were not adjustable, was unsuitable in our hands for this analysis. Using the local Kraken2 sequence classification system, we had fewer unclassified reads and detected a wider diversity of organisms in each river, concurrent with levels of diversity detected by other sequencing technologies. The web-based MG-RAST service provided zero unclassified reads and deeper, more comprehensive information, particularly with regard to xenobiotics, pathogens, and AMR. Comparative PCA analyses of these river metagenomes using both Kraken2 and MG-RAST data, at both the Family and Function levels (Figure 3), yielded highly similar groupings indicating that geographic proximity is far less important than the ecological functions being carried out by the predominantly microbial consortia. Such relationships among microbial consortia and urban/agriculture effects have been noted in other watersheds wherein, as for studies *op. cit*. here, riverine microbial consortia varied as a function of land use and environmental quality (Van Rossum et al. 2015; Vaz-Moreira, Nunes, and Manaia 2014).

Substantial compositional differences between geographically proximal sites

The differences among stories told by the metagenomes were striking in that these "snapshots" of microbes (Figure 5) and jobs that the microbial consortia in rivers and waterways are performing give signals that we believe could be used to enhance river management. For example, although Yare River samples collected west and east of Norwich yielded highly similar taxonomy and function, our two Vedder (River and Canal) samples, which were geographically closer than the Yare samples were to each other, yielded distinct taxonomic and functional arrays. The two Vedder sites had phenomenally different flow and anthropogenic impact. Vedder River is an actively flowing, relatively natural river area with a rock and gravel bottom and fed by an upstream lake of mountain rain and snow-melt runoff, whereas the downstream Vedder Canal is a channelized deep

artificial canal with minimal current flow, a mud bottom, and high sediment load. These physicochemical differences manifested in radically different consortia and therefore different predicted river functional pathways, illustrating how informative metagenomic analysis can be for investigating the interaction between geophysical site composition and bacterial community composition. The understanding derived from such observations will be particularly useful for river ecosystem management and indeed, is emerging as an important component of global change models.

Potential Implications

We demonstrate that yields of over 1M reads (i.e., 1 Gb data) are easily achievable with MinION, and further that yields of over 10 Gb are possible using the rapid PCR barcoding kit thus allowing for multiplexing of environmental samples. If one were planning multiplexed runs, our study shows this process should be sufficient for a quick indication of high-level metagenomic diversity. The analyses presented here illustrate that at the present average output, users interested in accurate assessment of taxonomy and function should strive for at least 250,000 reads per sample. Readers should be aware that nanopore sequencing is a new and disruptive technology in a state of constant improvement. As such, by the time of this publication, the approach presented here is anticipated to already have been enhanced through modification of MinION flow-cells, library chemistry, and bioinformatic capabilities.

Methods

Global river water sites

For this study, 11 diverse global riverine waterways (Table 1) were analyzed to compare the metagenomic diversity of microorganisms identified and to garner an initial understanding of microbial resistance genes present. In Europe, these rivers included River Yare (collections west

and east of Norwich, UK), River Rhine (Bimmen NL/DE), Neckar River (Tübingen, DE), River Corrib (Galway, IE), Sydhavnen (Copenhagen, DK), the Skævinge wastewater treatment plant (Zealand, DE). In the USA, rivers sampled included James River (Richmond, VA, USA) and Chena River (Fairbanks, AK, USA). In Canada, rivers sampled included Vedder River (Vancouver, CA) and St. Laurent River (Montreal, CA). A final site sampled in this study was the Karori Stream in New Zealand (Wellington, NZ).

River Yare is approximately 84 km long and flows from the West of Norfolk to the East coast, passing through the city of Norwich (urban population ~300,000). Outside the city center, most of the rest of the land that the river traverses is rural, with arable agriculture and tourism (sailing, motor boats). Two sample locations from this river were analyzed. This eastern sample was collected from the river bank by a public house in a small village, approximately 3 km downstream from the edge of Norwich. The upstream sample was obtained beside the University of East Anglia sports fields in a suburban area of Norwich.

River Rhine is one of Europe's largest rivers with a length of 1230 km. The sampling site was a pier extending into the river where the surrounding land was rural in character with mainly agricultural farmland. Upstream of the sampling point (the lower Rhine) consists of one of Europe's largest industrial and urban areas, the Ruhr area (urban population ~ 5 million).

Neckar River flows 362 km in Germany from the Black Forest to the Rhine River. Upstream of the sampling site near Tübingen (urban population ~ 100,000), the river flows through an area with a mix of villages, farmland, and forest. The sample was collected from a multi-lane divided bridge.

River Corrib in the west of Ireland is one of the shortest rivers in Europe. It flows 6 km from Lough Corrib to the Atlantic Ocean. Samples for analysis were taken from Upper Corrib region, 2 km upstream from the Galway city center (rural population ~80,000) in an unpopulated area where minor cattle and sheep grazing occur (pastoral farming).

Sydhavnen is a suburb of Copenhagen situated along Sluseløbet Canal and Copenhagen Harbour, directly connected to the Øresund (a sound that forms the border between Denmark and Sweden) in the northeast and the Baltic Sea in the southwest, stretching ~8 km. In addition to oceanic influence at both ends, the waterway, not technically a river, has heavy urban and transportation influence. The sample was collected in Sluseløbet Canal, beneath a bridge in Sydhavnen (urban population ~ 780,000), ~5 km below the Øresund.

Influent wastewater to the Skævinge wastewater treatment plant, not technically a river, was included to act as a control for high anthropogenic impact as it was assumed to contain human-associated bacteria. Skævinge wastewater treatment plant is located in a rural area of Zealand, Denmark, and treats wastewater from residential, industrial, and agricultural areas.

James River runs 560 km from the Appalachian Mountains to the Atlantic Ocean. The sampling site was in downtown Richmond, and is known to have heavy urban, industrial, and transportation influence. Near the site is one of the largest combined sewer overflow systems (CSOs) on the mid-Atlantic East Coast of North America and the site also receives local permitted input from construction, power plants, failing sewer systems, and industrial discharges resulting in elevated levels of polychlorinated biphenyls (PCBs). Upstream watershed activities include >170 active industrial discharges and >90 permitted pre-treatment discharge sites.

Chena River, the northern-most river in our sample, is spring-fed, stretches 160 km, and collects water from Interior Alaska. Samples were taken on the downstream side of the Moose Creek Dam and upstream of the populated areas of the Fairbanks North Star Borough.

Vedder River is a continuously flowing river 80 km in length that drains Chilliwack Lake, itself snow fed from the Cascade Mountains. The immediate area where the sample was collected was near Chilliwack suburbs and exhibited constant current flow. A second sample was collected from the Vedder Canal, a downstream artificial canal that drains into the Fraser River and is a main area for both swimming and salmon fishing. The canal is surrounded by earthen dykes that are immediately adjacent to active farming land on both flanks; there was little visible water movement at the time of sample collection.

Saint Lawrence River, running nearly 1200 km, is the third longest river in Canada. The sampling site was located off of Jean-Drapeau park ~5 km downstream of downtown Montreal (urban population ~1.8 million). This site is close to a municipal routine sampling site named FLS190 where past data have been collected and are available (https://bit.ly/1pDVTFg).

Karori Stream, the southern-most river in our sample, traverses ~10 km through Wellington, New Zealand with headwaters in bush and suburban areas, discharging into the sea at Wellington's south coast. The "Karori Stream at Makara Peak Mountain Bike Park" sampling site in Wellington is one of the Greater Wellington Regional Council's regular river sampling sites, in the middle reaches of the Karori Stream. There it also has some suburban and transportation influence.

Sample collection, DNA extraction, library generation, sequencing technology

Between April 2017 and October 2018, twelve laboratories with personnel exhibiting a wide diversity of skills and experience followed the standardized protocol (doi: 10.17504/protocols.io.qtgdwjw) outlined below for the filtering and extraction of DNA from shallow waters of 11 riverine waterways. Water samples were taken at 0.5-1 m depth during daylight hours at a time when neither drought nor recent excessive precipitation events occurred within one-week preceding sample collection. River water (2-4L) was collected for filtration in sterile collection bottles and were processed immediately or stored at 4°C for prolonged transportation time or until ready for filtration. The water samples were subsequently processed through a GF/C filter to remove suspended solids, particles etc. (size retention: 1.2 µm). The water recovered after GF/C filtering was subsequently filtered through a 0.22 um Durapore filter to capture microorganisms present. Upon completion of all filtering, nucleic acid was recovered using a modified procedure combining enzymatic lysis and purification using a DNeasy PowerWater DNA Isolation Kit (Qiagen). Briefly, each filter was aseptically transferred to a 5 ml tube. To this tube, a lysis mix was added which contained 1 ml of PW1 (DNeasy Power Water DNA isolation kit) and a previously described enzyme cocktail (Yuan et al. 2012) containing 100 µl lysozyme (10 mg/ml, Sigma-Aldrich), 12 µl mutanolysin (25 KU/ml, Sigma-Aldrich), and 6 µl lysostaphin (4000 U/ml, Sigma-Aldrich). The 5 ml tube with the lysis mixture was subsequently incubated at 37 °C for 1 hour, with gentle agitation to facilitate washing of the filters.

Steps 8-23 of the "experienced user protocol from the DNeasy Power Water DNA isolation kit" were followed. The eluted DNA was quantified using a Qubit fluorometer with the dsDNA HS kit. After quantification, a 0.4X SPRI bead clean-up of approx. 100 ng neat DNA was performed and eluted in 20 ul molecular grade water (or Tris-Cl pH 8-8.5). Subsequently 10-50 ng of DNA was used in conjunction with the Rapid Low Input by PCR Barcoding kit (SQK-RLB001, Oxford

Nanopore Tech) in accordance with manufacturers protocols to prepare a whole-genome sequencing libraries for use with a MinION device with minor alterations outlined below. A barcoding kit was chosen to facilitate multiplexing of negative controls and DNA from river samples to determine if any contamination was present during the processing of the river water templates. Modifications for the library preparation were i) 10-50 ng of input DNA and 2.5 µl of FRM were used for the tagmentation/fragmentation reaction and nuclease-free water was used to make the volume up to 10 µl, ii) for the PCR reaction, 20 cycles were used and the PCR reaction volumes were doubled. When multiplexing (negative filter and DNA from associated river samples), PCR products were pooled together in equal volumes, then subjected to a 0.6x AMPure XP bead wash and eluted in 12 µl of the buffer recommended in the manufacturer's instructions (10 µL 50 mM NaCl, 10 mM Tris-HCl pH 8.0).

After amplification a number of quality control checks were implemented to ensure successful library preparation was achieved. The quantity was assessed using the Qubit fluorometer dsDNA HS kit and DNA quality and estimated size distribution were subsequently determined via Tapestation, Bioanalyzer, or agarose gel. Following QC steps and removal of unincorporated primers, sequencing adapters were added to the mix and a room-temperature ligation-free reaction was carried out to link the adapters to the prepared DNA template. This prepared library (100-200 fmol) was then loaded into the MinION flow cell (R9.4) in accordance with manufacturers guidelines and the unit was run for a full 48 hours of sequencing.

Sequence processing, annotation, post-processing, and data analysis

Whole-genome shotgun sequenced reads were processed for basecalling and QC filtering using Albacore version 2.1.10 (Oxford Nanopore), and adapters were removed from the resulting DNA

sequence reads using Porechop version 0.2.3 (Wick 2019) using the command-line parameters '*porechop -i $INPUT -o $INPUT.porechop.fq --format fastq -t 32 --discard_middle*' (doi: 10.24433_CO.6736538). In a number of instances where replicate runs were performed for the same sample, the replicate data sets were pooled. The final adapter-trimmed data are accessible in both EBI (fast5) and MG-RAST (FASTQ, Table 1). Read lengths for sample-pooled FASTQ files were determined using a custom *fastx-length.pl* script, and processed into cumulative read length distribution plots and digital electrophoresis plots using a custom *length_plot.pl* script ((gringer) 2019).

To classify the sequence data for the purposes of identifying the microbial community in each water sample and to consider how these contribute to the ecology of each river ecosystem, FASTQ data initially were submitted to One Codex (based on the recommendation of Brown et al. 2017), an online pipeline that identifies microbial sequences using a k-mer based taxonomic classification algorithm, typically used for short-read data. The 2018 database was chosen for analysis which comprises a reference database that included approximately 80,000 bacterial, viral, fungal, and protozoan genomes. Reads also were processed using Kraken2 (Wood and Salzberg 2014), a different k-mer based sequence classification algorithm optimized for long-read sequences, which uses a publicly available pre-compiled genome database of bacteria, fungi, and viruses from RefSeq (Mockcommunity 2019). Lastly, sequences were uploaded via the command-line API and processed using MG-RAST (Keegan et al. 2016), a pipeline that for whole genome sequences first performs a protein similarity search between predicted proteins and database proteins and then provides bioinformatic tools to predict rDNA, gene, and protein functions with default parameters of: e-value $1{\times}e^{-5}$ (probability of chance incorrect annotation), identity 60 %, and a minimum alignment length of 15 (10-60 bp is common). Pavian plots of the representative taxa for each

metagenome (Breitwieser and Salzberg 2016) were constructed using the Kraken2 output. Using both MG-RAST and Kraken2 taxonomy results and the Bray-Curtis distance matrix among normalized family counts, Principal Components Analysis (PCA) was implemented, with one exception. For PCA on Kraken2 results, families were filtered to only include those that had fewer than 20% of samples with missing or zero counts.

To evaluate putative ecosystem-related functions from the reads, the MG-RAST server was used to compare data sets to three controlled annotation namespaces: Subsystems, KEGG Orthologues (KO), and Clusters of Orthologous Groups of proteins (COG). Normalized function data for each river sample were compared using PCA in MG-RAST (Subsystems Level 1, Minkowski distance matrix).

Data Availability

Raw signal FAST5 and FASTQ files are available from ENA via accessions numbers PRJEB34137 and ERP116996. Basecalled FASTQ read sets are archived in MG-RAST. Supplemental tables are available.

Competing interests

BLB, DE, JOG, JRT, MJ, HJ have received financial and non-financial benefits from Oxford Nanopore Technologies. Flow cells and library preparation kits were provided for the study by Oxford Nanopore Technologies at a group reduced charge.

Funding

ALD and DMD were supported by Alaska BLaST through the National Institute of General Medical Sciences of the National Institutes of Health under awards UL1GM118991,

TL4GM118992, and RL5GM118990 and Alaska INBRE, an Institutional Development Award (IDeA) from the National Institute of General Medical Sciences of the National Institutes of Health under grant number P20GM103395. TKN and LHH were supported by AUFF- NOVA grant AUFF-E-201 7-9-38. RML and DH's contribution to this research was funded by BBSRC grants BB/J004669/1 and BB/CSP17270/1. NP's contribution was funded by BBSRC grant BB/M011216/1. JRT was supported by Genome Canada Genomics Technology Platform grant, the Canada Foundation for Innovation (CFI) and the CFI Leaders Opportunity Fund (32557), Compute Canada Resource Allocation Project (WST-164-AB) and Genome Innovation Node (244819). BLB’s contribution to this research was funded by NSF DEB award number 1355059. TPS & JRT were supported by the Canada Research Chair in Biotechnology and Genomics-Neurobiology (TPS), the Canadian Institutes of Health Research (#10677; TPS), the Koerner Foundation (TPS).

Author Contributions

The study was conceived by JOG, MJ, JRT, KR, BLB; MJ coordinated the collaboration; BLB, DMD, DE, ALD, RML, DH, NP, HJ, LHH, TKN, JOG, HR, EvdH, AB, SO, JR, JRT, TPS, KR sampled rivers and performed sequencing; DMD, DE, ALD, HJ, JOG, BLB, MJ, and JRT analyzed and interpreted data; KR, LHH, TKN, and JOG developed and tested the protocol; DJ, MJ, JRT performed base-calling and data transfers; BLB and DE investigated One Codex analysis; JRT performed all Kraken2 analysis; BLB, DE conducted PCA analyses; BLB, HJ, and DMD uploaded and performed MG-RAST analysis; BLB, DE, DMD, ALD, RML, LHH, TKN, KR, TB, JOG, EvdH, JRT, MJ, and TPS wrote and edited the manuscript.

Acknowledgements

The Cloud Infrastructure for Microbial Bioinformatics (CLIMB) service in the U.K. was used to facilitate the upload and transfer of raw FAST5 files. We are thankful to Ewan Birney (EBI) for providing advice on the project and on the consortium. We thank Elizabeth Harvey for her deep insight into the possible explanations for the higher than expected viral loads in the Sydhavnen sample. We are grateful to Amanda Hodges for H_2O quality data, and Henk Zemmelink from Rijkswaterstaat, the Netherlands for allowing us to use their facilities in Bimmen and monitoring data from that day. Rosemary Dokos and Alina Ham (Oxford Nanopore Technologies) provided advice and assistance on technology, especially barcoding chemistry and negative controls.

References

Amos, G. C. A., P. M. Hawkey, W. H. Gaze, and E. M. Wellington. 2014. "Waste Water Effluent Contributes to the Dissemination of CTX-M-15 in the Natural Environment." *The Journal of Antimicrobial Chemotherapy* 69 (7): 1785–91.

Azam, F., T. Fenchel, J. G. Field, J. S. Gray, L. A. Meyer-Reil, and F. Thingstad. 1983. "The Ecological Role of Water-Column Microbes in the Sea." *Marine Ecology Progress Series*. https://doi.org/10.3354/meps010257.

Beaulaurier, John, Elaine Luo, John Eppley, Paul Den Uyl, Xiaoguang Dai, Daniel J. Turner, Matthew Pendelton, Sissel Juul, Eoghan Harrington, and Edward F. DeLong. Accessed 29 July 2019. "Assembly-Free Single-Molecule Nanopore Sequencing Recovers Complete Virus Genomes from Natural Microbial Communities." https://doi.org/10.1101/619684.

Bertrand, Denis, Jim Shaw, Manesh Kalathiyappan, Amanda Hui Qi Ng, M. Senthil Kumar, Chenhao Li, Mirta Dvornicic, et al. 2019. "Hybrid Metagenomic Assembly Enables High-Resolution Analysis of Resistance Determinants and Mobile Elements in Human Microbiomes." *Nature Biotechnology,* July. https://doi.org/10.1038/s41587-019-0191-2.

Biller, Steven J, and 19 co-authors. 2018. Marine microbial metagenomes sampled across space and time.

Scientific Data 5:180176, https://doi.org/10.1038/sdata.2018.176

Bramblett, Robert G., and Kurt D. Fausch. 1991. “Variable Fish Communities and the Index of Biotic Integrity in a Western Great Plains River.” *Transactions of the American Fisheries Society* 120 (6): 752–69.

Breitwieser, Florian P., and Steven L. Salzberg. 2016. “Pavian: Interactive Analysis of Metagenomics Data for Microbiomics and Pathogen Identification.” *bioRxiv*. https://doi.org/10.1101/084715.

Brown, Bonnie L., Rebecca V. LePrell, Rima B. Franklin, Maria C. Rivera, Francine M. Cabral, Hugh L. Eaves, Vicki Gardiakos, Kevin P. Keegan, and Timothy L. King. 2015. “Metagenomic Analysis of Planktonic Microbial Consortia from a Non-Tidal Urban-Impacted Segment of James River.” *Standards in Genomic Sciences* 10 (September): 65.

Brown, Bonnie L., Mick Watson, Samuel S. Minot, Maria C. Rivera, and Rima B. Franklin. 2017. “MinION™ Nanopore Sequencing of Environmental Metagenomes: A Synthetic Approach.” *GigaScience* 6 (3): 1–10.

Davies, Susan P., and Susan K. Jackson. 2006. “The Biological Condition Gradient: A Descriptive Model for Interpreting Change in Aquatic Ecosystems.” *Ecological Applications: A Publication of the Ecological Society of America* 16 (4): 1251–66.

Ghai, Rohit, Francisco Rodriguez-Valera, Katherine D. McMahon, Danyelle Toyama, Raquel Rinke, Tereza Cristina Souza de Oliveira, José Wagner Garcia, Fernando Pellon de Miranda, and Flavio Henrique-Silva. 2011. “Metagenomics of the Water Column in the Pristine Upper Course of the Amazon River.” *PloS One* 6 (8): e23785.

(gringer), David Eccles. 2019. *Gringer/bioinfscripts: Tree Lab / Global River Release*. https://doi.org/10.5281/zenodo.3240748.

Hamner, Steve, Bonnie L. Brown, Nur A. Hasan, Michael J. Franklin, John Doyle, Margaret J. Eggers, Rita R. Colwell, and Timothy E. Ford. 2019. “Metagenomic Profiling of Microbial Pathogens in the Little Bighorn River, Montana.” *International Journal of Environmental Research and Public Health* 16 (7). https://doi.org/10.3390/ijerph16071097.

Holben, William E. 2011. “GC Fractionation Allows Comparative Total Microbial Community Analysis, Enhances Diversity Assessment, and Facilitates Detection of Minority Populations of Bacteria.” *Handbook of Molecular Microbial Ecology I: Metagenomics and Complementary Approaches. New York: John Wiley & Sons, Inc*, 183–96.

Junger, Pedro C., André M. Amado, Rodolfo Paranhos, Anderson S. Cabral, Saulo M. S. Jacques, and Vinicius F. Farjalla. 2018. “Salinity Drives the Virioplankton Abundance but Not Production in Tropical Coastal Lagoons.” *Microbial Ecology* 75 (1): 52–63.

Karr, J. R. 1981. “Assessment of Biotic Integrity Using Fish Communities.” *Fisheries*. https://afspubs.onlinelibrary.wiley.com/doi/abs/10.1577/1548-8446(1981)006%3C0021:AOBIUF%3E2.0.CO;2.

Keegan KP, EM Glass, F Meyer. 2016. MG-RAST, a Metagenomics Service for Analysis of Microbial Community Structure and Function. pp 207-233 *In*: Martin F and S Uroz (eds) Microbial Environmental Genomics (MEG). Methods in Molecular Biology, vol 1399. Humana Press, New York, NY

Kirchman, D. L., A. I. Dittel, S. E. G. Findlay, and D. Fischer. 2004. “Changes in Bacterial Activity and Community Structure in Response to Dissolved Organic Matter in the Hudson River, New York.” *Aquatic Microbial Ecology: International Journal* 35: 243–57.

Kristiansson, Erik, Jerker Fick, Anders Janzon, Roman Grabic, Carolin Rutgersson, Birgitta Weijdegård, Hanna Söderström, and D. G. Joakim Larsson. 2011. “Pyrosequencing of Antibiotic-Contaminated River Sediments Reveals High Levels of Resistance and Gene Transfer Elements.” *PloS One* 6 (2): e17038.

Mockcommunity. Github. Accessed 29 July 2019. https://github.com/LomanLab/mockcommunity.

Murray, Aimee K., Lihong Zhang, Xiaole Yin, Tong Zhang, Angus Buckling, Jason Snape, and William H. Gaze. 2018. “Novel Insights into Selection for Antibiotic Resistance in Complex Microbial Communities.” *mBio* 9 (4). https://doi.org/10.1128/mBio.00969-18.

Newton, Ryan J., Stuart E. Jones, Alexander Eiler, Katherine D. McMahon, and Stefan Bertilsson. 2011.

"A Guide to the Natural History of Freshwater Lake Bacteria." *Microbiology and Molecular Biology Reviews: MMBR* 75 (1): 14–49.

Oh, Seungdae, Alejandro Caro-Quintero, Despina Tsementzi, Natasha DeLeon-Rodriguez, Chengwei Luo, Rachel Poretsky, and Konstantinos T. Konstantinidis. 2011. "Metagenomic Insights into the Evolution, Function, and Complexity of the Planktonic Microbial Community of Lake Lanier, a Temperate Freshwater Ecosystem." *Applied and Environmental Microbiology* 77 (17): 6000–6011.

Pernthaler, Jakob. 2013. "Freshwater Microbial Communities." In *The Prokaryotes: Prokaryotic Communities and Ecophysiology*, edited by Eugene Rosenberg, Edward F. DeLong, Stephen Lory, Erko Stackebrandt, and Fabiano Thompson, 97–112. Berlin, Heidelberg: Springer Berlin Heidelberg.

Rodgers, Christopher. 2017. "A New Approach to Protecting Ecosystems: The Te Awa Tupua (Whanganui River Claims Settlement) Act 2017." *Environmental Law Review* 19 (4): 266–79.

Shade, Ashley, Cayelan C. Carey, Emily Kara, Stefan Bertilsson, Katherine D. McMahon, and Matthew C. Smith. 2009. "Can the Black Box Be Cracked? The Augmentation of Microbial Ecology by High-Resolution, Automated Sensing Technologies." *The ISME Journal* 3 (8): 881–88.

Staley, Christopher, Trevor J. Gould, Ping Wang, Jane Phillips, James B. Cotner, and Michael J. Sadowsky. 2014. "Core Functional Traits of Bacterial Communities in the Upper Mississippi River Show Limited Variation in Response to Land Cover." *Frontiers in Microbiology* 5 (August): 414.

Staley, C., T. Unno, T. J. Gould, B. Jarvis, J. Phillips, J. B. Cotner, and M. J. Sadowsky. 2013. "Application of Illumina next-Generation Sequencing to Characterize the Bacterial Community of the Upper Mississippi River." *Journal of Applied Microbiology* 115 (5): 1147–58.

Su, Jian-Qiang, Yu Xia, Huai-Ying Yao, Ya-Ying Li, Xin-Li An, Brajesh K. Singh, Tong Zhang, and Yong-Guan Zhu. 2017. "Metagenomic Assembly Unravel Microbial Response to Redox Fluctuation in Acid Sulfate Soil." *Soil Biology and Biochemistry*. https://doi.org/10.1016/j.soilbio.2016.11.027.

Sunagawa, Shinichi, and 50 co-authors. 2015. STructure and function of the global ocean microbiome. *Science*. 348:1261359, https://doi.org/10.1126/science.1261359

Van Rossum, Thea, Michael A. Peabody, Miguel I. Uyaguari-Diaz, Kirby I. Cronin, Michael Chan, Jared

R. Slobodan, Matthew J. Nesbitt, et al. 2015. “Year-Long Metagenomic Study of River Microbiomes Across Land Use and Water Quality.” *Frontiers in Microbiology* 6 (December): 1405.

Vaz-Moreira, Ivone, Olga C. Nunes, and Célia M. Manaia. 2014. “Bacterial Diversity and Antibiotic Resistance in Water Habitats: Searching the Links with the Human Microbiome.” *FEMS Microbiology Reviews* 38 (4): 761–78.

Wick, Ryan. *Porechop*. Github. Accessed 1 August 2019. https://github.com/rrwick/Porechop.

Winter, Christian, Thomas Hein, Gerhard Kavka, Robert L. Mach, and Andreas H. Farnleitner. 2007. “Longitudinal Changes in the Bacterial Community Composition of the Danube River: A Whole-River Approach.” *Applied and Environmental Microbiology* 73 (2): 421–31.

Wood, Derrick E., and Steven L. Salzberg. 2014. “Kraken: Ultrafast Metagenomic Sequence Classification Using Exact Alignments.” *Genome Biology* 15 (3): R46.

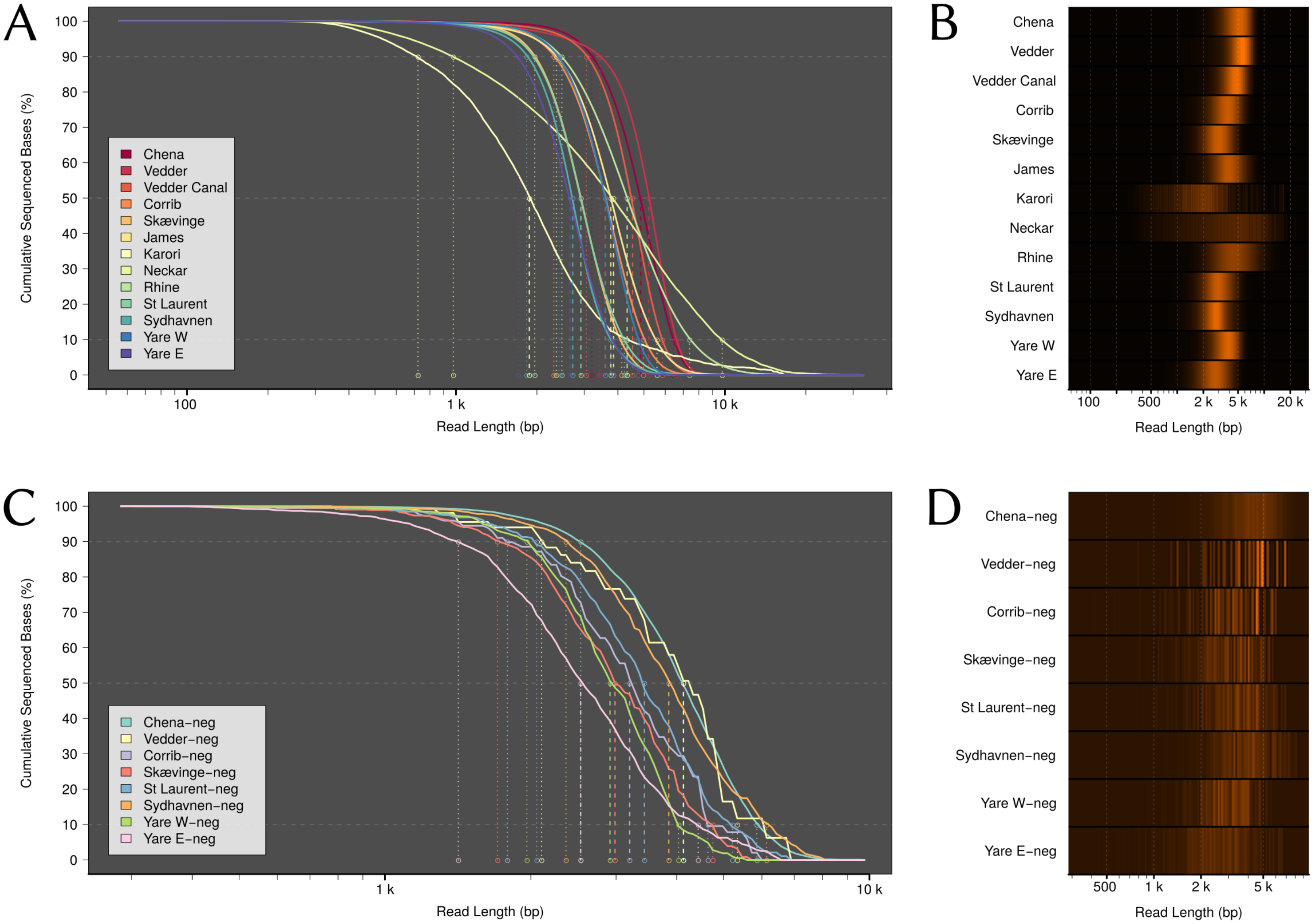
A
B
C
D
Cumulative Sequenced Bases (%)
Read Length (bp)
Chena
Vedder
Vedder Canal
Corrib
Skævinge
James
Karori
Neckar
Rhine
St Laurent
Sydhavnen
Yare W
Yare E
Chena–neg
Vedder–neg
Corrib–neg
Skævinge–neg
St Laurent–neg
Sydhavnen–neg
Yare W–neg
Yare E–neg
100
1 k
10 k
500
2 k
5 k
20 k

692

Figure 1: Read length distribution plots for sample (A and B) and negative (C and D) sequencing libraries. The cumulative sequenced bases plots (A and C) allow read length percentiles to be identified; read N10, N50, and N90 are indicated on the plot by vertical lines and a pair of circles. The digital electrophoresis plots (B and D) show the distribution of read lengths in the libraries, as might be seen via gel electrophoresis. The sample libraries generally show a very tight read length distribution (except for the low-count samples, Karori and Neckar), whereas the negative samples have platykurtic length distribution curves. Not shown in panel D: James-neg (because only one sequence, *H. sapiens*, ATP synthase, was detected, 1.67 kbp).

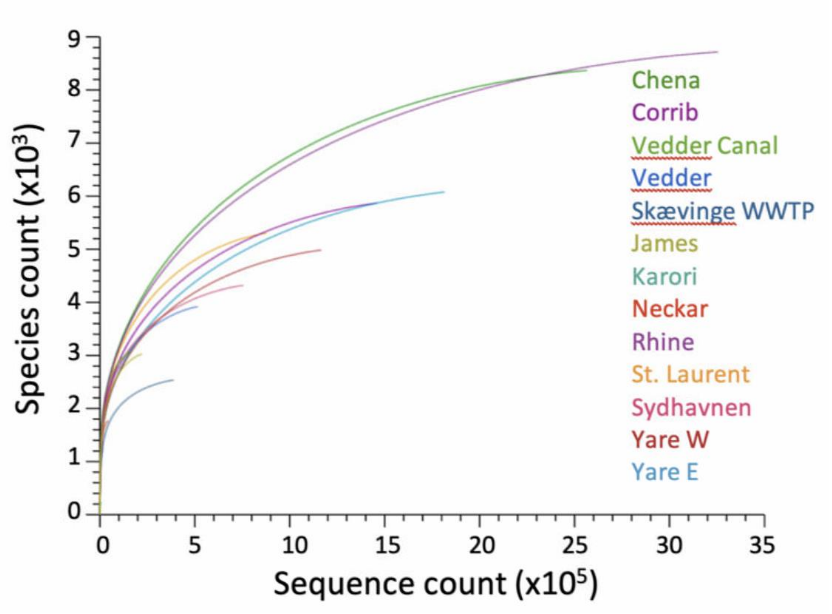

Figure 2. Rarefaction curves of annotated species for 13 samples from 11 rivers and waterways.

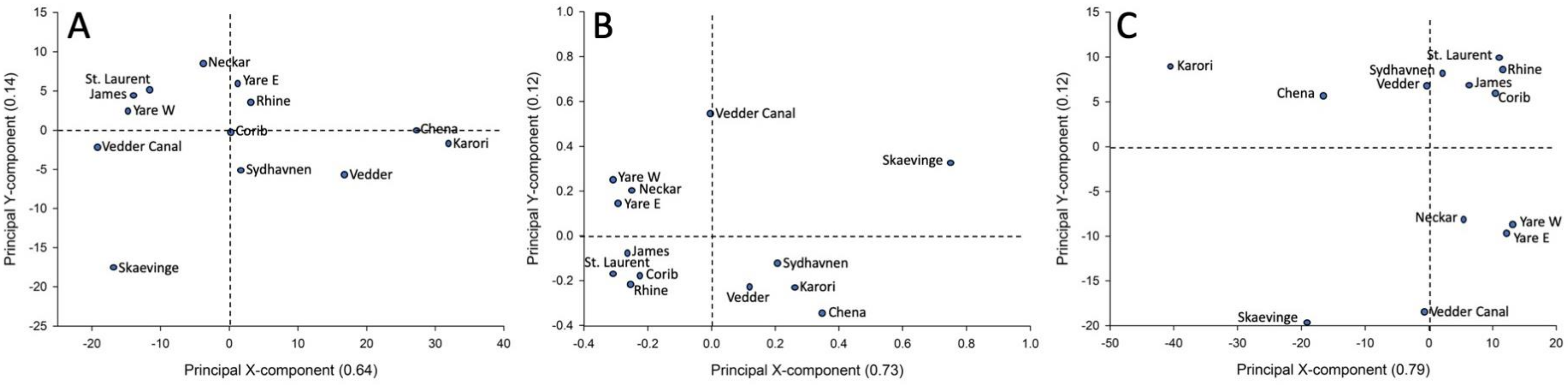

Figure 3. Concurrence of PCAs based on normalized data among 13 metagenomes from 11 rivers and waterways. A: Families annotated in Kraken2, B: Families annotated in MG-RAST, C: Subsystem functions identified by MG-RAST.

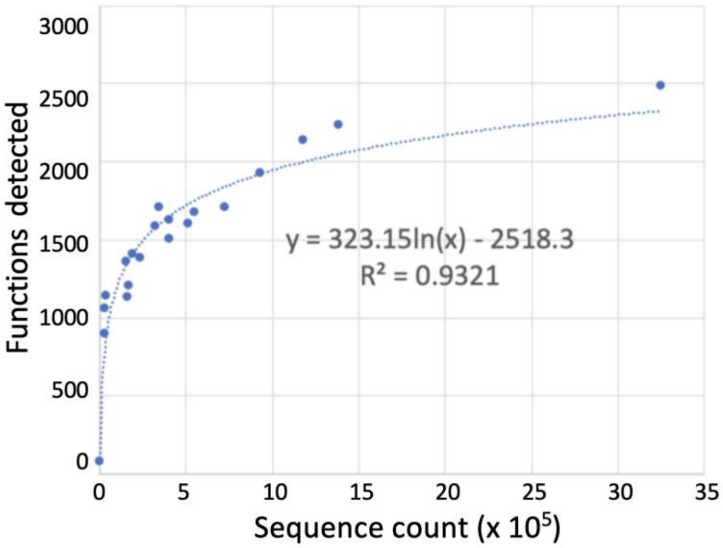

Figure 4. Distribution of detected Subsystems functions *versus* sequence count for 13 river and waterway metagenomes.

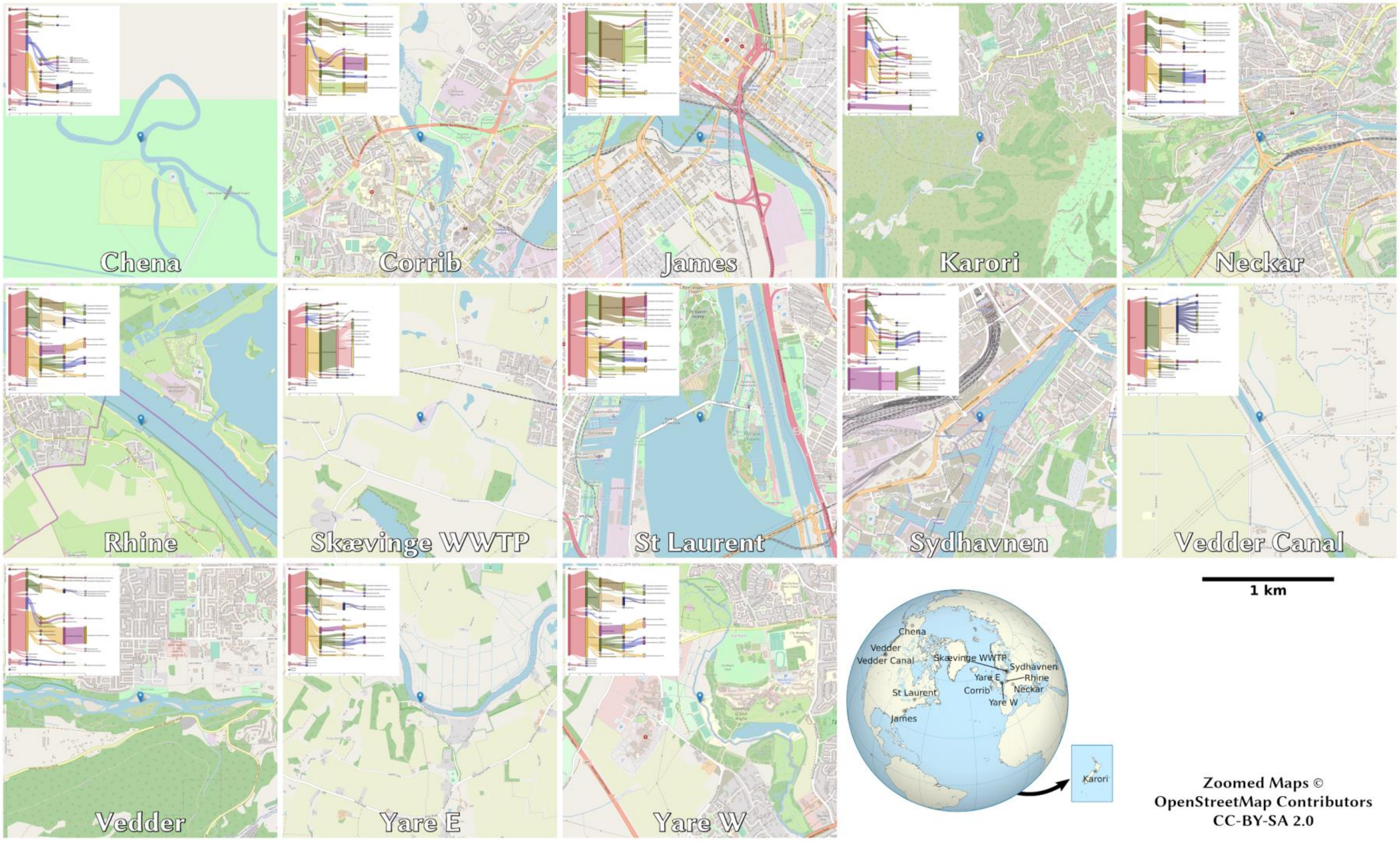

Figure 5. Representative taxonomy in each of 13 riverine metagenomes overlaid upon a map of each site to illustrate the range of sites sampled and the relative diversity of taxa identified.

Table 1. Site, study, and sample information. The notation "NA" in parentheses is included where negative read set files included too few reads to be analyzed in MG-RAST. Where accessions are given in parentheses, those refer to the barcoded negative control data. All FASTQ files are available from ENA via accessions numbers PRJEB34137 and ERP116996, individual indexing is in progress as of 30 August 2019.

River Name	Lat Long	Date	Lab ID	URID	Region	ENA	MG-RAST (negative)
River Yare W	52.620751, 1.229554	2017-04-24	4	Yare W	Norwich UK	TBD	mgl735606 (mgl735602)
River Yare E	52.6054, 1.3825	2017-10-11	9	Yare E	Norwich UK	TBD	mgl735614 (mgl735603)
Rhine River	51.861409, 6.068399	2017-07-05	5	Rhine	Bimmen NL/DE	TBD	mgl735607 (NA)
Neckar River	48.516222, 9.04575	2017-06-26	12	Neckar	Tübingen DE	TBD	mgl735617 (NA)
River Corrib	53.281242, -9.059482	2018-02-05	13	Corrib	Galway IE	TBD	mgl735618 (NA)

Sydhavnen	55.662192, 12.564093	2018-03-17	14	Sydhavnen	København DK	TBD	mgl735619 (mgl735604)
Skævinge WWTP	55.90583, 12.12806	2017-05-30	17	Skævinge WWTP	Skaevinge DK	TBD	mgl735623 (NA)
James River	37.527941, -77.436144	2017-06-13	15	James	Richmond USA	TBD	mgl735622 (NA)
Chena River	64.79583, -147.19361	2018-10-26	18	Chena	Fairbanks USA	TBD	mgl735626 (mgl735605)
Vedder River	49.0973, -121.9858	2017-08-28	6/A	Vedder	Vancouver CA	TBD	mgl735613 (NA)
Vedder Canal	49.1052, -122.079	2017-08-28	6/B	Vedder Canal	Vancouver CA	TBD	mgl735610 (NA)
St. Laurent River	45.50609, -73.53414	2017-10-13	11	St Laurent	Montreal CA	TBD	mgl735616 (NA)
Karori Stream	-41.29699, 174.72226	2017-11-01	10	Karori	Wellington NZ	TBD	mgl735615 (NA)

Table 2. Long-read metagenome properties and downstream analysis summary. L50: length of the shortest read in the set of the longest 50% of base-called data, N50: number of reads in the set of the longest 50% of base-called data, rRNA: number of reads that contain ribosomal RNA genes, Features: predicted proteins, CDS: identified proteins, Subsystems: number of reads assigned to all Subsystem level 1 functional categories, Anthro: percentage of reads with predicted protein functions annotated to Virulence, Disease and Defense, Phages, Prophages, Transposable elements, Plasmids, Metabolism of Aromatic Compounds, and Stress Response, α-Diversity: estimated from the distribution of the species-level annotations.

**River**	**Reads**	**Bases (Gbp)**	**L50 (N50)**	**rRNA**	**Features**	**CDS**	**Subsystem reads**	**% Anthro**	**α-Diversity**
River Yare W	$1.2\cdot10^6$	3.8	3.6 kb ($4.5\cdot10^5$)	4799	$4.3\cdot10^6$	$7.5\text{x}10^5$	$1.7\cdot10^6$	6.6	296
River Yare E	$1.8\cdot10^6$	4.4	2.6 kb ($6.7\cdot10^5$)	648,702	$5.1\cdot10^6$	$6.5\cdot10^5$	$1.4\cdot10^6$	7.5	443
Rhine River	$3.2\cdot10^6$	12.6	4.3 kb ($1.1\cdot10^6$)	13,412	$13.9\cdot10^6$	$1.8\cdot10^6$	$4.1\cdot10^6$	6.6	492
Neckar River	$4.2\cdot10^4$	0.1	3.8 kb ($7.4\cdot10^3$)	203	$1.1\cdot10^5$	$2.5\cdot10^4$	$5.5\cdot10^5$	7.1	316

River Corrib	$1.5\cdot10^6$	4.8	3.6 kb ($5.4\cdot10^5$)	4,467	$5.6\cdot10^6$	$6.8\cdot10^5$	$1.5\cdot10^6$	6.4	413
Sydhavnen	$7.6\cdot10^5$	1.9	2.7 kb ($2.9\cdot10^5$)	1,712	$2.3\cdot10^6$	$3.0\cdot10^5$	$3.9\cdot10^5$	9.5	156
Skævinge WWTP	$3.9\cdot10^5$	1.0	2.9 kb ($1.5\cdot10^5$)	1,436	$1.4\cdot10^6$	$1.6\cdot10^5$	$2.6\cdot10^5$	7.3	218
James River	$2.2\cdot10^5$	0.8	3.8 kb ($8.0\cdot10^5$)	627	$8.14\cdot10^5$	$1.4\cdot10^5$	$3.4\cdot10^5$	6.0	452
Chena River	$2.6\cdot10^6$	11.4	4.8 kb ($9.8\cdot10^5$)	4,242	$12.4\cdot10^6$	$8.9\cdot10^5$	$1.8\cdot10^6$	7.1	1139
Vedder River	$1.6\cdot10^5$	0.7	5.2 kb ($5.9\cdot10^4$)	430	$7.8\cdot10^5$	$7.3\cdot10^5$	$1.5\cdot10^5$	18.9	486
Vedder Canal	$5.2\cdot10^5$	2.1	4.5 kb ($2.0\cdot10^5$)	640,261	$2.2\cdot10^6$	$6.4\cdot10^5$	$1.3\cdot10^6$	6.2	135
St. Laurent River	$8.8\cdot10^5$	2.4	2.9 kb ($3.3\cdot10^5$)	2,561	$2.8\cdot10^6$	$5.8\cdot10^5$	$1.3\cdot10^6$	6.1	480
Karori Stream	$7.0\cdot10^4$	0.01	1.9 kb	27	$1.4\cdot10^4$	$3.2\cdot10^2$	$4.8\cdot10^2$	7.2	178

			$(1.7 \cdot 10^3)$						

Table 3. Normalized proportions of 64 genera that were detected at >0.01% in any of 13 river metagenome samples analyzed by MinION, listed with the most commonly noted genera at the top of the table. ND: not detected. Green shaded cells are occurrence > 0.01. *: unclassified derived from this group. YW: Yare River West, YE: Yare River East, RR: Rhine River, NR: Neckar River, SY: Sydhavnen, SK: Skævinge, JR: James River, CR: Chena River, VR: Vedder River, VC: Vedder Canal, SL: St. Laurent River, KS: Karori Stream.

Genus	YW	YE	RR	NR	RC	SY	SK	JR	CR	VR	VC	SL	KS
Flavobacteriaceae	0.175	0.157	0.045	0.132	0.059	0.096	0.011	0.044	0.026	0.108	0.480	0.034	0.036
Flavobacteriales*	0.035	0.029	0.004	0.022	0.009	0.005	0.002	0.004	0.003	0.015	0.071	0.004	0.004
Flavobacteria*	0.006	0.005	0.003	0.006	0.003	0.055	0.000	0.003	0.001	0.004	0.012	0.002	0.000
Comamonadaceae	0.159	0.165	0.098	0.134	0.114	0.008	0.007	0.054	0.023	0.022	0.059	0.137	0.040
Burkholderiaceae	0.158	0.081	0.066	0.037	0.080	0.015	0.004	0.077	0.024	0.027	0.017	0.066	0.051
Burkholderiales*	0.018	0.017	0.012	0.013	0.014	0.002	0.001	0.008	0.004	0.005	0.006	0.016	0.016
Cytophagaceae	0.030	0.041	0.030	0.069	0.035	0.010	0.002	0.030	0.009	0.027	0.073	0.024	0.029
Streptomycetaceae	0.019	0.016	0.031	0.015	0.015	0.004	0.000	0.068	0.006	0.008	0.002	0.046	0.000
Sphingobacteriaceae	0.018	0.018	0.022	0.043	0.022	0.008	0.002	0.022	0.007	0.014	0.027	0.014	0.009
Sphingobacteriales*	0.013	0.009	0.027	0.045	0.024	0.002	0.001	0.036	0.003	0.007	0.010	0.018	0.000
Alcaligenaceae	0.016	0.008	0.009	0.006	0.013	0.003	0.001	0.006	0.004	0.003	0.002	0.010	0.016
Oxalobacteraceae	0.016	0.012	0.013	0.006	0.013	0.003	0.001	0.014	0.005	0.005	0.003	0.013	0.033
Micrococcaceae	0.012	0.015	0.013	0.010	0.007	0.002	0.000	0.031	0.003	0.005	0.002	0.019	0.000
Microbacteriaceae	0.012	0.016	0.006	0.007	0.003	0.001	0.000	0.015	0.001	0.002	0.002	0.008	0.000
Methylophilaceae	0.011	0.015	0.026	0.011	0.052	0.013	0.001	0.026	0.006	0.009	0.013	0.026	0.002
Methylophilales*	0.000	0.000	0.004	0.000	0.008	0.025	0.000	0.003	0.000	0.001	0.000	0.004	0.000
Rhodocyclaceae	0.010	0.011	0.009	0.007	0.012	0.004	0.004	0.007	0.008	0.004	0.003	0.010	0.007
Mycobacteriaceae	0.008	0.007	0.013	0.007	0.007	0.002	0.000	0.024	0.004	0.004	0.001	0.020	0.009
Pseudomonadaceae	0.008	0.010	0.009	0.008	0.010	0.010	0.007	0.006	0.011	0.009	0.004	0.008	0.089
Bacteroidaceae	0.007	0.010	0.008	0.016	0.008	0.004	0.061	0.008	0.010	0.007	0.012	0.006	0.022

Nocardioidaceae	0.007	0.006	0.012	0.006	0.006	0.001	0.000	0.026	0.002	0.003	0.001	0.018	0.000
Sphingomonadaceae	0.007	0.008	0.013	0.032	0.008	0.004	0.001	0.007	0.007	0.010	0.002	0.008	0.007
Rhodobacteraceae	0.007	0.012	0.020	0.035	0.018	0.049	0.003	0.007	0.012	0.026	0.005	0.022	0.020
Frankiaceae	0.007	0.005	0.012	0.006	0.006	0.002	0.000	0.025	0.003	0.004	0.001	0.018	0.000
Enterobacteriaceae	0.006	0.009	0.008	0.006	0.009	0.008	0.018	0.006	0.011	0.011	0.004	0.007	0.024
Pseudonocardiaceae	0.006	0.005	0.010	0.005	0.005	0.001	0.000	0.021	0.002	0.003	0.001	0.015	0.000
Corynebacteriaceae	0.005	0.005	0.008	0.004	0.004	0.001	0.001	0.016	0.004	0.003	0.001	0.011	0.000
Bradyrhizobiaceae	0.005	0.007	0.013	0.007	0.014	0.007	0.002	0.005	0.012	0.018	0.004	0.013	0.007
Nocardiaceae	0.005	0.005	0.008	0.004	0.004	0.001	0.000	0.017	0.002	0.002	0.001	0.012	0.002
Porphyromonadaceae	0.005	0.005	0.005	0.011	0.005	0.003	0.009	0.005	0.006	0.005	0.008	0.004	0.009
Micromonosporaceae	0.005	0.004	0.008	0.004	0.004	0.001	0.000	0.017	0.002	0.003	0.001	0.012	0.004
Nocardiopsaceae	0.004	0.003	0.007	0.004	0.004	0.001	0.000	0.014	0.001	0.002	0.000	0.010	0.000
Bacillaceae	0.004	0.005	0.007	0.005	0.007	0.005	0.007	0.005	0.024	0.010	0.004	0.006	0.002
Intrasporangiaceae	0.004	0.003	0.005	0.003	0.003	0.001	0.000	0.012	0.001	0.001	0.000	0.008	0.000
Acidothermaceae	0.004	0.003	0.006	0.003	0.003	0.001	0.000	0.013	0.001	0.002	0.000	0.010	0.000
Kineosporiaceae	0.004	0.003	0.005	0.003	0.002	0.000	0.000	0.011	0.001	0.001	0.000	0.007	0.000
Chlorobiaceae	0.004	0.004	0.005	0.006	0.005	0.002	0.002	0.004	0.015	0.006	0.005	0.004	0.007
Clostridiaceae	0.003	0.005	0.006	0.005	0.006	0.004	0.013	0.004	0.035	0.011	0.003	0.004	0.013
Prevotellaceae	0.003	0.004	0.003	0.006	0.003	0.002	0.013	0.003	0.004	0.003	0.004	0.002	0.004
Vibrionaceae	0.003	0.003	0.004	0.004	0.004	0.005	0.006	0.003	0.005	0.005	0.002	0.003	0.016
Cyclobacteriaceae	0.003	0.004	0.009	0.014	0.006	0.002	0.000	0.008	0.001	0.004	0.006	0.006	0.002
Rhizobiaceae	0.003	0.005	0.008	0.005	0.008	0.006	0.001	0.004	0.007	0.011	0.002	0.007	0.007
Gammaproteobacteria*	0.003	0.004	0.005	0.003	0.005	0.014	0.002	0.003	0.004	0.004	0.002	0.004	0.004
Moraxellaceae	0.003	0.004	0.004	0.002	0.004	0.004	0.014	0.002	0.004	0.004	0.002	0.003	0.020
Chroococcales*	0.003	0.004	0.005	0.006	0.004	0.006	0.002	0.014	0.017	0.008	0.003	0.004	0.007
Rhodospirillaceae	0.002	0.007	0.007	0.003	0.008	0.005	0.002	0.003	0.008	0.015	0.002	0.006	0.002
Alteromonadaceae	0.002	0.002	0.003	0.002	0.003	0.006	0.002	0.002	0.003	0.003	0.001	0.002	0.027
Geobacteraceae	0.002	0.003	0.003	0.002	0.003	0.002	0.002	0.002	0.017	0.007	0.002	0.002	0.004
Caulobacteraceae	0.002	0.003	0.005	0.006	0.005	0.003	0.001	0.002	0.005	0.008	0.002	0.004	0.011
Rickettsiales*	0.001	0.001	0.061	0.000	0.069	0.100	0.000	0.002	0.002	0.132	0.012	0.038	0.004
Planctomycetaceae	0.001	0.003	0.006	0.003	0.004	0.002	0.001	0.003	0.009	0.006	0.002	0.003	0.013

Campylobacteraceae	0.001	0.003	0.002	0.002	0.002	0.002	0.534	0.001	0.009	0.004	0.001	0.001	0.024
Helicobacteraceae	0.001	0.002	0.002	0.001	0.002	0.002	0.061	0.001	0.009	0.004	0.001	0.001	0.002
Bdellovibrionaceae	0.001	0.001	0.001	0.001	0.001	0.001	0.000	0.001	0.004	0.021	0.003	0.001	0.007
Peptococcaceae	0.001	0.002	0.002	0.001	0.002	0.001	0.001	0.002	0.014	0.003	0.001	0.002	0.013
Thermoanaerobacteraceae	0.001	0.001	0.002	0.001	0.002	0.001	0.001	0.001	0.015	0.003	0.001	0.002	0.018
Aeromonadaceae	0.001	0.001	0.001	0.001	0.001	0.001	0.016	0.001	0.001	0.001	0.001	0.001	0.002
Epsilonproteobacteria*	0.000	0.000	0.001	0.000	0.001	0.001	0.018	0.000	0.003	0.001	0.000	0.000	0.004
Phycodnaviridae	0.000	0.000	0.003	0.000	0.001	0.217	0.000	0.000	0.000	0.002	0.000	0.000	0.000
Myoviridae	0.000	0.001	0.005	0.000	0.004	0.038	0.001	0.001	0.001	0.014	0.001	0.002	0.002
Mamiellales*	0.000	0.001	0.001	0.000	0.000	0.016	0.000	0.000	0.001	0.001	0.000	0.000	0.000
Nitrosopumilaceae	0.000	0.000	0.000	0.000	0.001	0.015	0.000	0.000	0.003	0.004	0.000	0.000	0.002
Campylobacterales*	0.000	0.000	0.000	0.000	0.000	0.000	0.011	0.000	0.001	0.000	0.000	0.000	0.000
Bacteria*	0.001	0.001	0.001	0.001	0.001	0.000	0.002	0.001	0.013	0.002	0.000	0.001	0.000

Table 4. Normalized percent abundances of functions annotated through KO and COG. ND indicates "not detected." River abbreviations are as shown in Table 3.

Function	YW	YE	RR	NR	RC	SY	SK	JR	CR	VR	VC	SL	KS
**KO**													
Cellular processes	4	4	4	4	4	5	8	4	5	5	4	4	4
Environmental information processing	11	11	10	11	10	9	10	10	11	9	9	11	16
Genetic information processing	23	24	24	22	24	23	24	24	39	26	23	24	28
Human diseases	1	1	1	1	1	2	1	1	1	1	1	1	ND
Metabolism	60	59	61	61	61	61	57	61	43	58	62	60	52
Organismal systems	1	1	1	1	1	1	ND	1	1	1	1	1	ND
**COG**													
Amino acid transport/metabolism	11	11	12	12	12	12	11	13	6	10	11	12	10
Carbo transport/metabolism	6	6	6	6	6	5	5	7	6	6	6	6	6
Cell cycle control, cell division, chromosome partitioning	1	1	1	1	2	1	1	2	3	2	1	1	ND
Cell motility	<1	<1	<1	<1	<1	<1	1	<1	<1	<1	<1	<1	1
Cell wall/membrane/ envelope biogenesis	6	6	5	6	5	5	7	5	6	6	7	5	3
Chromatin structure/dynamics	<1	<1	<1	<1	<1	<1	ND	<1	<1	<1	<1	<1	ND
Coenzyme transport/metabolism	5	5	5	5	5	5	5	5	3	5	5	5	4
Cytoskeleton	<1	<1	<1	<1	<1	<1	<1	<1	<1	<1	<1	<1	<1
Defense mechanisms	2	2	1	2	1	1	2	1	2	1	2	1	3
Energy production/conversion	11	10	12	10	12	11	10	11	6	10	9	12	7
Extracellular structures	<1	<1	<1	ND	<1	<1	<1	<1	<1	<1	<1	<1	ND
Function unknown	<1	<1	<1	<1	<1	<1	<1	<1	<1	<1	<1	<1	ND
General function prediction	10	10	9	10	9	8	9	8	6	8	11	9	9
Inorganic ion transport/metabolism	4	4	4	4	4	4	6	3	3	3	4	4	3
Intracellular trafficking, secretion, and vesicular transport	2	2	2	2	2	2	2	2	4	3	2	2	1
Lipid transport/metabolism	5	5	5	5	5	4	3	5	2	4	5	5	1
Nucleotide transport/metabolism	5	5	6	5	6	7	5	6	5	6	5	5	6
Post-translational modification, protein turnover, chaperones	6	6	6	5	6	7	5	6	8	7	5	6	10

Replication, recombination, repair	7	8	7	7	7	6	7	7	12	8	7	7	9
RNA processing/modification	<1	<1	<1	<1	<1	<1	<1	<1	<1	<1	<1	<1	ND
Secondary metabolites biosynthesis, transport, catabolism	2	2	2	2	2	2	1	2	1	1	1	2	1
Signal transduction mechanisms	2	2	2	2	2	1	5	2	2	2	1	2	6
Transcription	4	4	4	4	4	4	4	4	5	4	4	4	6
Translation, ribosomal struct, biogenesis	12	12	13	12	13	11	11	13	17	13	12	13	11

Table 5. Normalized percentage abundances, where a function was represented at >= 0.1% of annotations, of KO pathways detected related to processing of xenobiotic substances or to human or plant pathogens and diseases, and of COG pathways relating to antibiotic or multidrug resistance, toxins, or virulence. River abbreviations are as shown in Table 3.

**Database and function**	**YW**	**YE**	**RR**	**NR**	**RC**	**SY**	**SK**	**JR**	**CR**	**VR**	**VC**	**SL**	**KS**
**KO**													
05134 Legionellosis [PATH:ko05134]	0.3	0.4	0.4	0.4	0.4	0.4	ND	0.4	0.1	0.3	0.3	0.4	ND
05203 Viral carcinogenesis [PATH:ko05203]	0.2	0.2	0.2	0.2	0.2	0.1	0.2	0.2	0.3	0.2	0.2	0.2	ND
00362 Benzoate degradation [PATH:ko00362]	0.3	0.3	0.2	0.2	0.2	0.1	ND	0.1	0.1	0.1	0.1	0.2	ND
05200 Pathways in cancer [PATH:ko05200]	0.1	0.1	0.2	0.3	0.2	0.2	ND	0.1	0.1	0.3	0.1	0.1	ND
05010 Alzheimer's disease [PATH:ko05010]	ND	0.1	0.1	0.1	0.2	0.3	ND	0.1	0.1	0.2	ND	0.1	ND
00361 Chlorocyclohexane and chlorobenzene degradation [PATH:ko00361]	0.2	0.1	0.1	0.1	0.1	0.1	ND	0.1	ND	ND	0.1	0.1	ND
05120 Epithelial cell signaling in Helicobacter pylori infection [PATH:ko05120]	0.2	0.2	0.1	0.1	0.1	0.1	0.2	0.1	0.1	ND	0.2	0.1	ND
05204 Chemical carcinogenesis [PATH:ko05204]	0.1	0.1	0.1	0.2	0.1	0.1	ND	0.1	0.1	0.1	ND	0.1	ND
00627 Aminobenzoate degradation [PATH:ko00627]	0.1	0.1	0.1	0.1	0.1	0.1	ND	ND	ND	ND	ND	0.1	ND
05219 Bladder cancer [PATH:ko05219]	0.1	0.1	0.1	ND	ND	ND	ND	0.2	0.1	0.1	ND	0.1	ND
00633 Nitrotoluene degradation [PATH:ko00633]	0.1	ND	0.1	ND	0.1	ND	ND	ND	ND	0.1	ND	0.1	ND
05142 Chagas disease (American trypanosomiasis) [PATH:ko05142]	ND	ND	ND	0.1	0.1	0.1	ND	ND	ND	0.2	0.2	ND	ND
05340 Primary immunodeficiency [PATH:ko05340]	0.1	0.1	0.1	ND	ND	ND	ND	0.1	ND	0.1	ND	0.1	ND
00984 Steroid degradation [PATH:ko00984]	ND	ND	ND	ND	ND	ND	ND	ND	ND	ND	ND	0.1	ND
00791 Atrazine degradation [PATH:ko00791]	ND	ND	ND	0.1	ND	ND	ND	ND	ND	ND	ND	ND	ND
00983 Drug metabolism - other enzymes [PATH:ko00983]	ND	ND	ND	ND	ND	ND	ND	ND	ND	ND	0.1	ND	0.6
**COG**													
Cation/multidrug efflux pump													
ABC-type multidrug transport system, ATPase and permease	0.4	0.4	0.3	0.3	0.3	0.2	0.2	0.2	0.5	0.3	0.5	0.3	2.1

ABC-type multidrug transport system, ATPase component	0.2	0.2	0.2	0.3	0.2	0.2	0.1	0.3	0.4	0.3	0.2	0.3	ND
Permeases of the drug / metabolite transporter (DMT) superfamily	0.1	0.1	0.1	0.1	0.1	0.1	0.1	ND	ND	ND	0.1	0.1	ND
ABC-type multidrug transport system, permease component	0.1	0.1	0.1	0.1	0.1	0.1	ND	0.1	0.1	0.1	0.1	0.1	ND
Beta-lactamase class C and other penicillin binding proteins	0.1	0.1	ND	0.1	ND	ND	ND	ND	ND	ND	0.2	ND	ND
ABC-type bacteriocin / lantibiotic exporters, contain N-terminal double-glycine peptidase domain	ND	ND	ND	ND	ND	ND	0.2	ND	ND	ND	0.1	ND	0.7
