# Supplementary material for: Metagenomic analysis of planktonic riverine microbial consortia using nanopore sequencing reveals insight into river microbe taxonomy and function
Source: Gigascience. 2020 Jun 10;9(6):giaa053. doi: 10.1093/gigascience/giaa053 (PMC7285869; doi:10.1093/gigascience/giaa053)
Supplement: giaa053_Supplemental_Files [file giaa053_supplemental_files.zip › GIGA-S-19-00534-3_pavian.pdf]

Chena

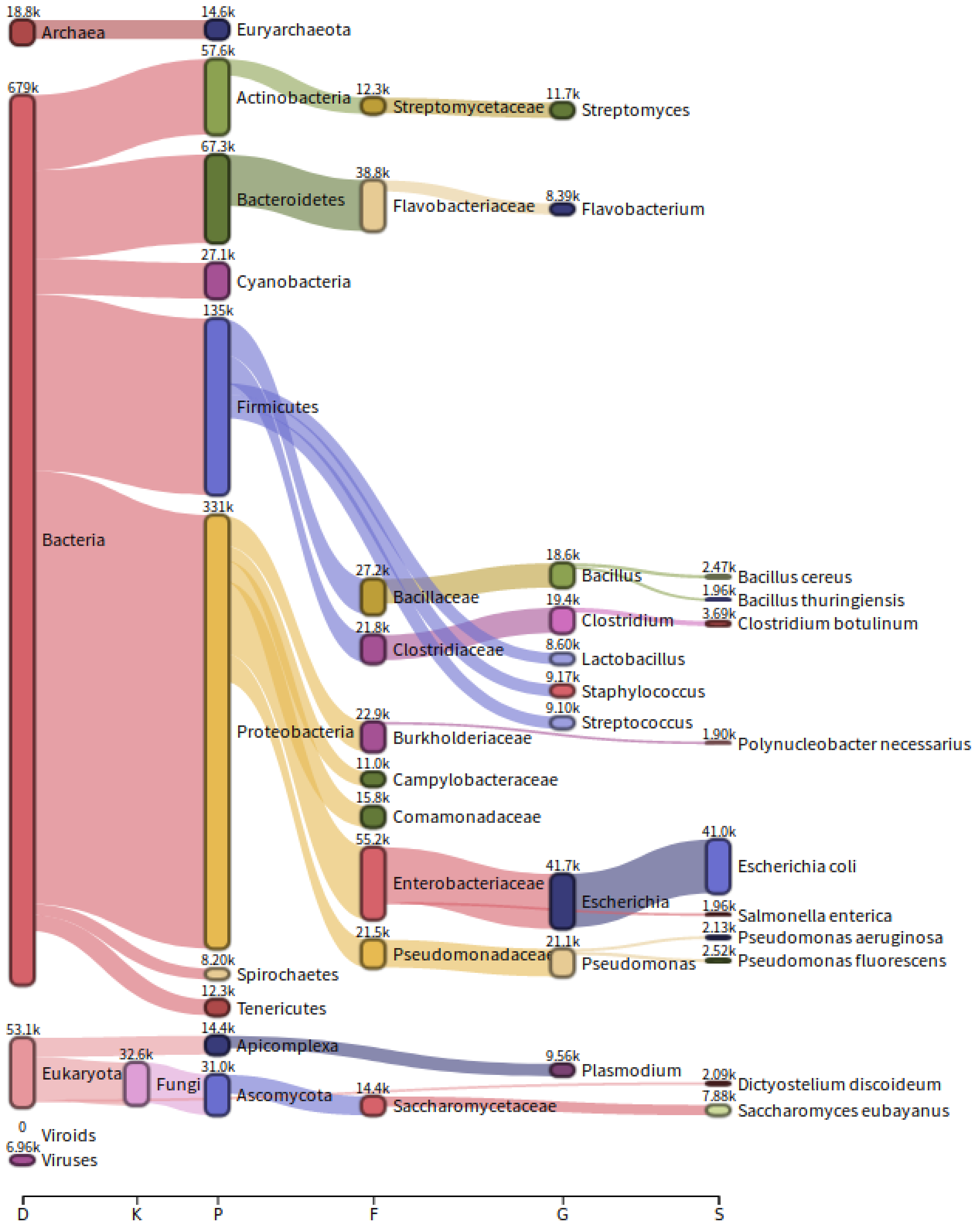

18.8k
Archaea
14.6k
Euryarchaeota
57.6k
Actinobacteria
12.3k
Streptomycetaceae
11.7k
Streptomyces
679k
67.3k
Bacteroidetes
38.8k
Flavobacteriaceae
8.39k
Flavobacterium
27.1k
Cyanobacteria
135k
Firmicutes
331k
Bacteria
18.6k
Bacillus
2.47k
Bacillus cereus
27.2k
Bacillaceae
1.96k
Bacillus thuringiensis
19.4k
Clostridium
3.69k
Clostridium botulinum
21.8k
Clostridiaceae
8.60k
Lactobacillus
9.17k
Staphylococcus
9.10k
Streptococcus
22.9k
Proteobacteria
Burkholderiaceae
1.90k
Polynucleobacter necessarius
11.0k
Campylobacteraceae
15.8k
Comamonadaceae
41.0k
55.2k
Escherichia coli
Enterobacteriaceae
41.7k
Escherichia
1.96k
Salmonella enterica
21.5k
2.13k
Pseudomonas aeruginosa
Pseudomonadaceae
21.1k
2.52k
Pseudomonas fluorescens
Pseudomonas
8.20k
Spirochaetes
12.3k
Tenericutes
53.1k
14.4k
Apicomplexa
32.6k
Eukaryota
31.0k
9.56k
Plasmodium
Fungi
2.09k
Dictyostelium discoideum
Ascomycota
14.4k
7.88k
Saccharomycetaceae
Saccharomyces eubayanus
0
Viroids
6.96k
Viruses
D
K
P
F
G
S

Corrib

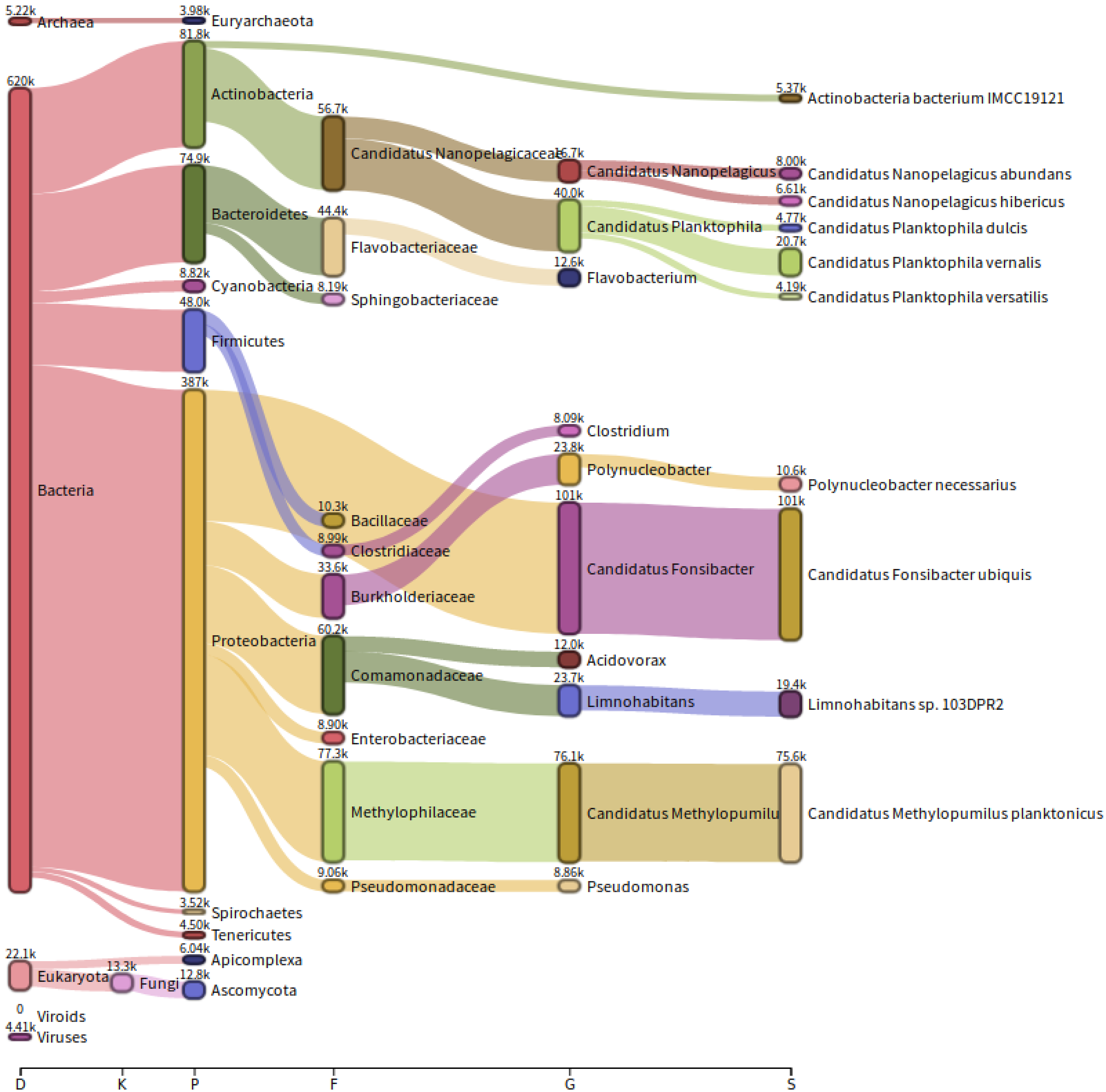

5.22k Archaea
3.98k Euryarchaeota
81.8k Actinobacteria
620k Bacteria
5.37k Actinobacteria bacterium IMCC19121
56.7k Candidatus Nanopelagicaceae
16.7k Candidatus Nanopelagicus
8.00k Candidatus Nanopelagicus abundans
6.61k Candidatus Nanopelagicus hibericus
74.9k Bacteroidetes
44.4k Flavobacteriaceae
40.0k Candidatus Planktophila
4.77k Candidatus Planktophila dulcis
20.7k Candidatus Planktophila vernalis
12.6k Flavobacterium
8.82k Cyanobacteria
8.19k Sphingobacteriaceae
4.19k Candidatus Planktophila versatilis
48.0k Firmicutes
387k Proteobacteria
8.09k Clostridium
23.8k Polynucleobacter
10.6k Polynucleobacter necessarius
101k Candidatus Fonsibacter
101k Candidatus Fonsibacter ubiquis
10.3k Bacillaceae
8.99k Clostridiaceae
33.6k Burkholderiaceae
60.2k Comamonadaceae
12.0k Acidovorax
23.7k Limnohabitans
19.4k Limnohabitans sp. 103DPR2
8.90k Enterobacteriaceae
77.3k Methylophilaceae
76.1k Candidatus Methylopumilu
75.6k Candidatus Methylopumilus planktonicus
9.06k Pseudomonadaceae
8.86k Pseudomonas
3.52k Spirochaetes
4.50k Tenericutes
22.1k Eukaryota
13.3k Fungi
6.04k Apicomplexa
12.8k Ascomycota
0 Viroids
4.41k Viruses
D
K
P
F
G
S

James

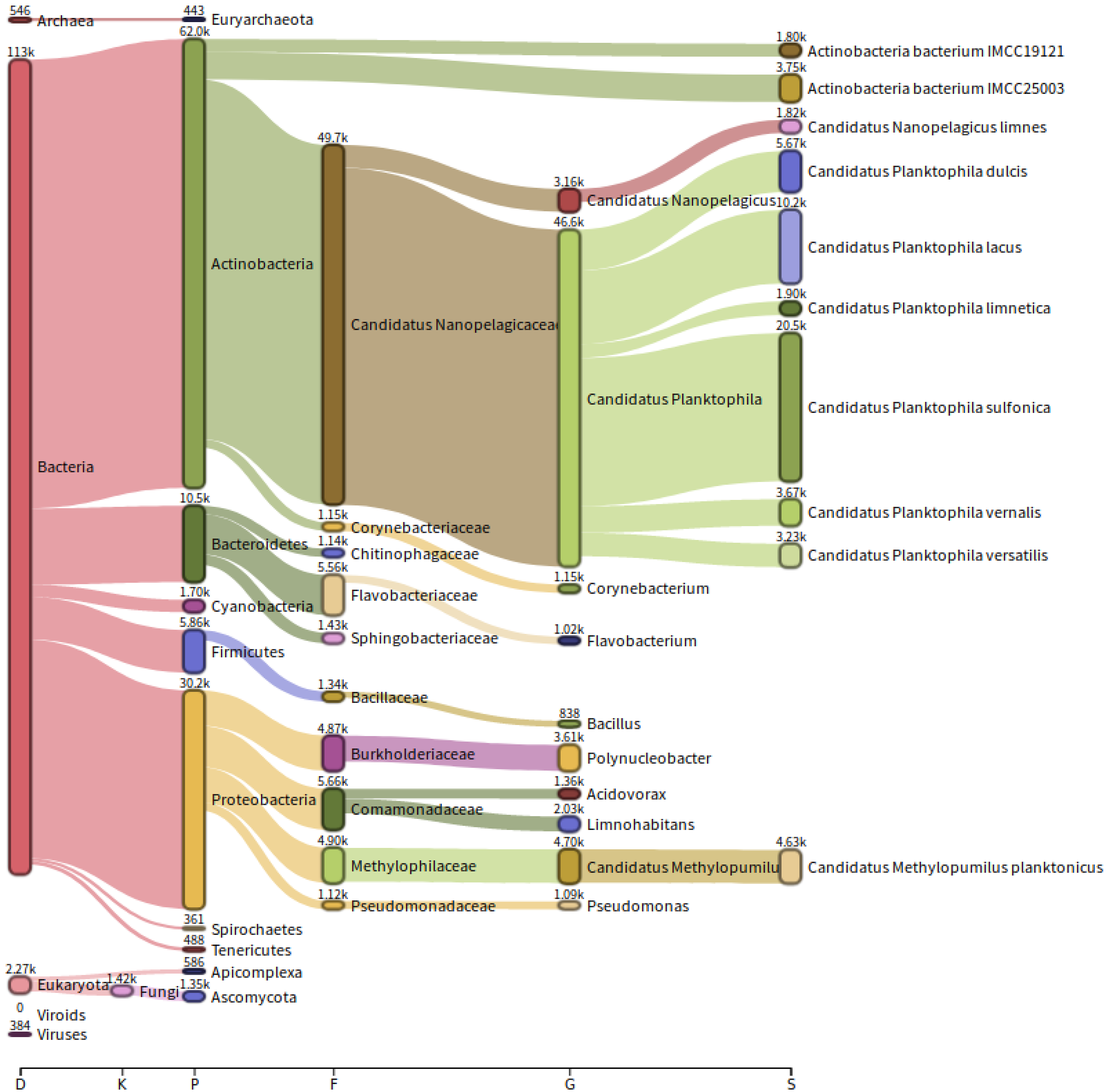
546
Archaea
443
Euryarchaeota
62.0k
113k
1.80k
Actinobacteria bacterium IMCC19121
3.75k
Actinobacteria bacterium IMCC25003
1.82k
Candidatus Nanopelagicus limnes
5.67k
Candidatus Planktophila dulcis
49.7k
3.16k
Candidatus Nanopelagicus
10.2k
46.6k
Candidatus Planktophila lacus
Actinobacteria
1.90k
Candidatus Planktophila limnetica
20.5k
Candidatus Nanopelagicaceae
Candidatus Planktophila
Candidatus Planktophila sulfonica
Bacteria
10.5k
3.67k
1.15k
Candidatus Planktophila vernalis
Corynebacteriaceae
3.23k
Bacteroidetes
1.14k
Candidatus Planktophila versatilis
Chitinophagaceae
5.56k
1.15k
Corynebacterium
1.70k
Flavobacteriaceae
Cyanobacteria
5.86k
1.43k
1.02k
Sphingobacteriaceae
Flavobacterium
Firmicutes
30.2k
1.34k
Bacillaceae
838
Bacillus
4.87k
3.61k
Burkholderiaceae
Polynucleobacter
5.66k
1.36k
Proteobacteria
Acidovorax
Comamonadaceae
2.03k
Limnohabitans
4.90k
4.70k
4.63k
Methylophilaceae
Candidatus Methylopumilu
Candidatus Methylopumilus planktonicus
1.12k
1.09k
Pseudomonadaceae
Pseudomonas
361
Spirochaetes
488
Tenericutes
2.27k
586
Apicomplexa
1.42k
Eukaryota
Fungi
1.35k
Ascomycota
0
Viroids
384
Viruses
D
K
P
F
G
S

Karori

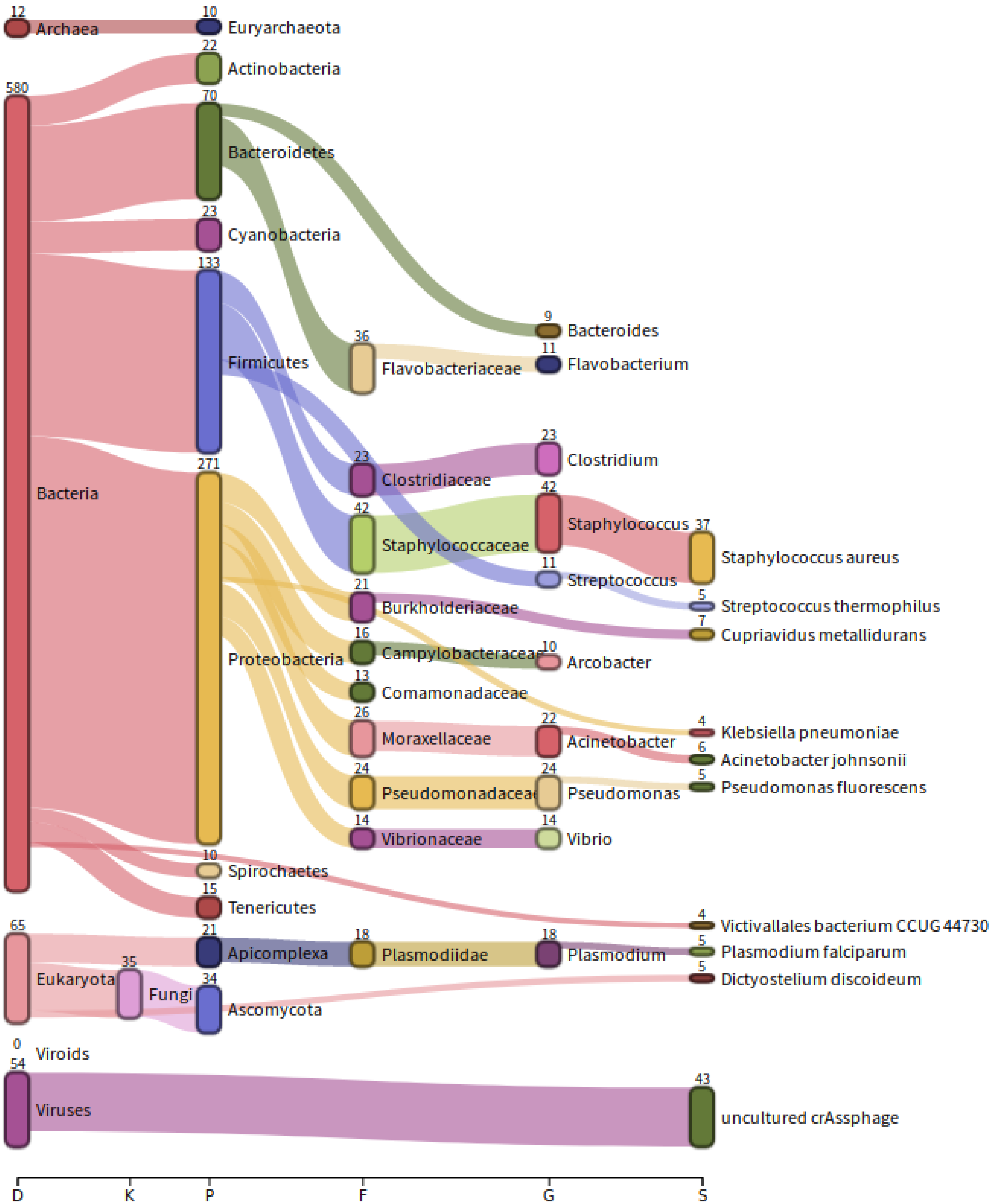
12 Archaea
10 Euryarchaeota
22 Actinobacteria
580 Bacteria
70 Bacteroidetes
23 Cyanobacteria
133 Firmicutes
271 Proteobacteria
10 Spirochaetes
15 Tenericutes
36 Flavobacteriaceae
9 Bacteroides
11 Flavobacterium
23 Clostridiaceae
23 Clostridium
42 Staphylococcaceae
42 Staphylococcus
37 Staphylococcus aureus
11 Streptococcus
5 Streptococcus thermophilus
21 Burkholderiaceae
7 Cupriavidus metallidurans
16 Campylobacteraceae
10 Arcobacter
13 Comamonadaceae
26 Moraxellaceae
22 Acinetobacter
4 Klebsiella pneumoniae
6 Acinetobacter johnsonii
24 Pseudomonadaceae
24 Pseudomonas
5 Pseudomonas fluorescens
14 Vibrionaceae
14 Vibrio
4 Victivallales bacterium CCUG 44730
65 Eukaryota
21 Apicomplexa
18 Plasmodiidae
18 Plasmodium
5 Plasmodium falciparum
5 Dictyostelium discoideum
35 Fungi
34 Ascomycota
0 Viroids
54 Viruses
43 uncultured crAssphage
D
K
P
F
G
S

Neckar

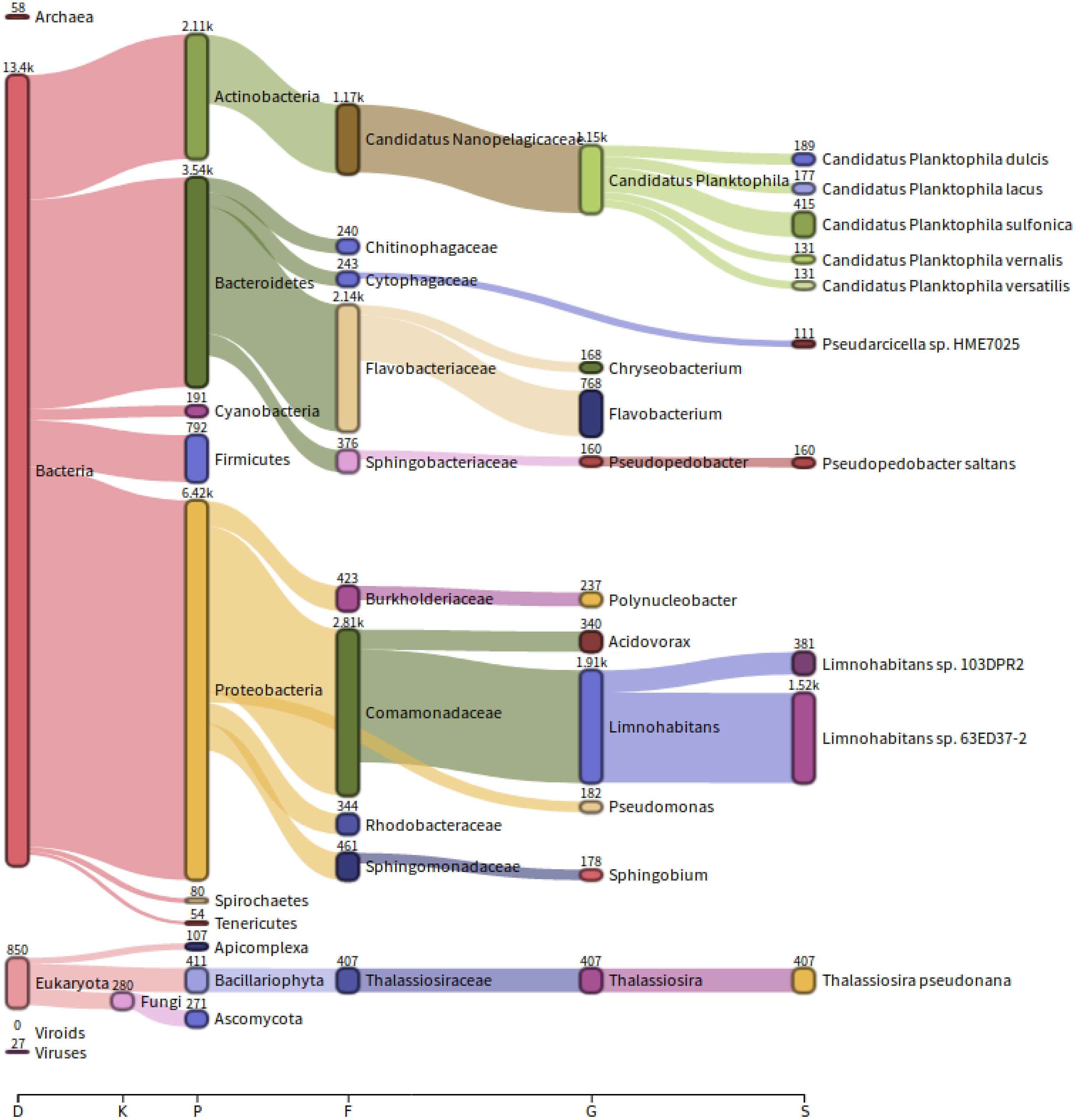
58 Archaea
13.4k Bacteria
2.11k Actinobacteria
3.54k Bacteroidetes
191 Cyanobacteria
792 Firmicutes
6.42k Proteobacteria
80 Spirochaetes
54 Tenericutes
1.17k Candidatus Nanopelagicaceae
1.15k Candidatus Planktophila
189 Candidatus Planktophila dulcis
177 Candidatus Planktophila lacus
415 Candidatus Planktophila sulfonica
131 Candidatus Planktophila vernalis
131 Candidatus Planktophila versatilis
240 Chitinophagaceae
243 Cytophagaceae
111 Pseudarcicella sp. HME7025
2.14k Flavobacteriaceae
168 Chryseobacterium
768 Flavobacterium
376 Sphingobacteriaceae
160 Pseudopedobacter
160 Pseudopedobacter saltans
423 Burkholderiaceae
237 Polynucleobacter
2.81k Comamonadaceae
340 Acidovorax
1.91k Limnohabitans
381 Limnohabitans sp. 103DPR2
1.52k Limnohabitans sp. 63ED37-2
182 Pseudomonas
344 Rhodobacteraceae
461 Sphingomonadaceae
178 Sphingobium
850 Eukaryota
280 Fungi
107 Apicomplexa
411 Bacillariophyta
271 Ascomycota
407 Thalassiosiraceae
407 Thalassiosira
407 Thalassiosira pseudonana
0 Viroids
27 Viruses
D
K
P
F
G
S

Rhine

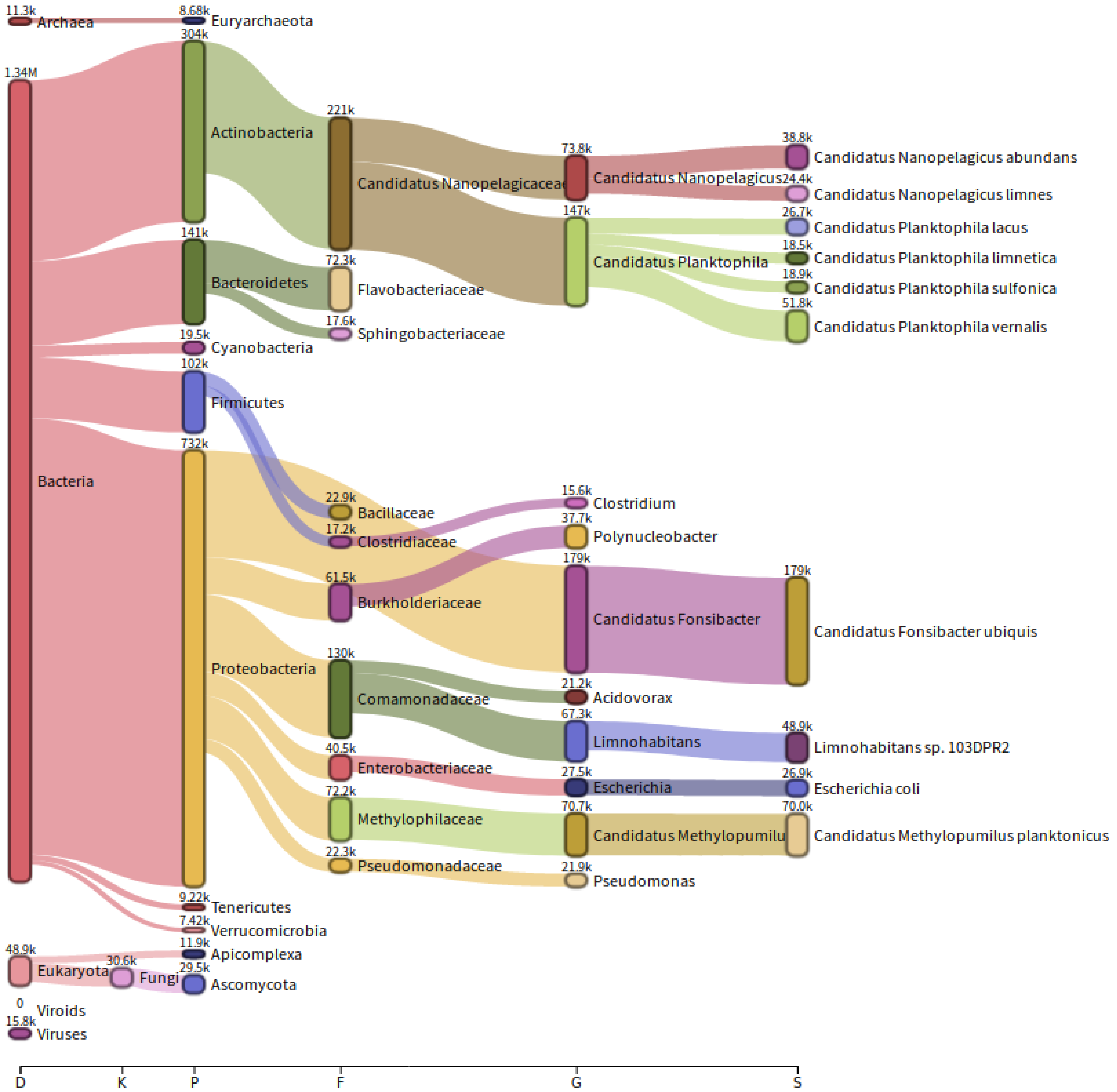

11.3k
Archaea
8.68k
Euryarchaeota
304k
1.34M
Actinobacteria
221k
73.8k
38.8k
Candidatus Nanopelagicus abundans
Candidatus Nanopelagicaceae
Candidatus Nanopelagicus
24.4k
Candidatus Nanopelagicus limnes
147k
26.7k
Candidatus Planktophila lacus
141k
18.5k
Candidatus Planktophila limnetica
72.3k
Candidatus Planktophila
Bacteroidetes
18.9k
Candidatus Planktophila sulfonica
Flavobacteriaceae
51.8k
17.6k
Candidatus Planktophila vernalis
Sphingobacteriaceae
19.5k
Cyanobacteria
102k
Firmicutes
732k
Bacteria
22.9k
15.6k
Bacillaceae
Clostridium
17.2k
37.7k
Clostridiaceae
Polynucleobacter
179k
179k
61.5k
Burkholderiaceae
Candidatus Fonsibacter
Candidatus Fonsibacter ubiquis
Proteobacteria
130k
21.2k
Comamonadaceae
Acidovorax
67.3k
48.9k
Limnohabitans
Limnohabitans sp. 103DPR2
40.5k
Enterobacteriaceae
27.5k
26.9k
Escherichia
Escherichia coli
72.2k
70.7k
70.0k
Methylophilaceae
Candidatus Methylopumilu
Candidatus Methylopumilus planktonicus
22.3k
21.9k
Pseudomonadaceae
Pseudomonas
9.22k
Tenericutes
7.42k
Verrucomicrobia
48.9k
11.9k
Apicomplexa
30.6k
29.5k
Eukaryota
Fungi
Ascomycota
0
Viroids
15.8k
Viruses
D
K
P
F
G
S

Skævinge WWTP

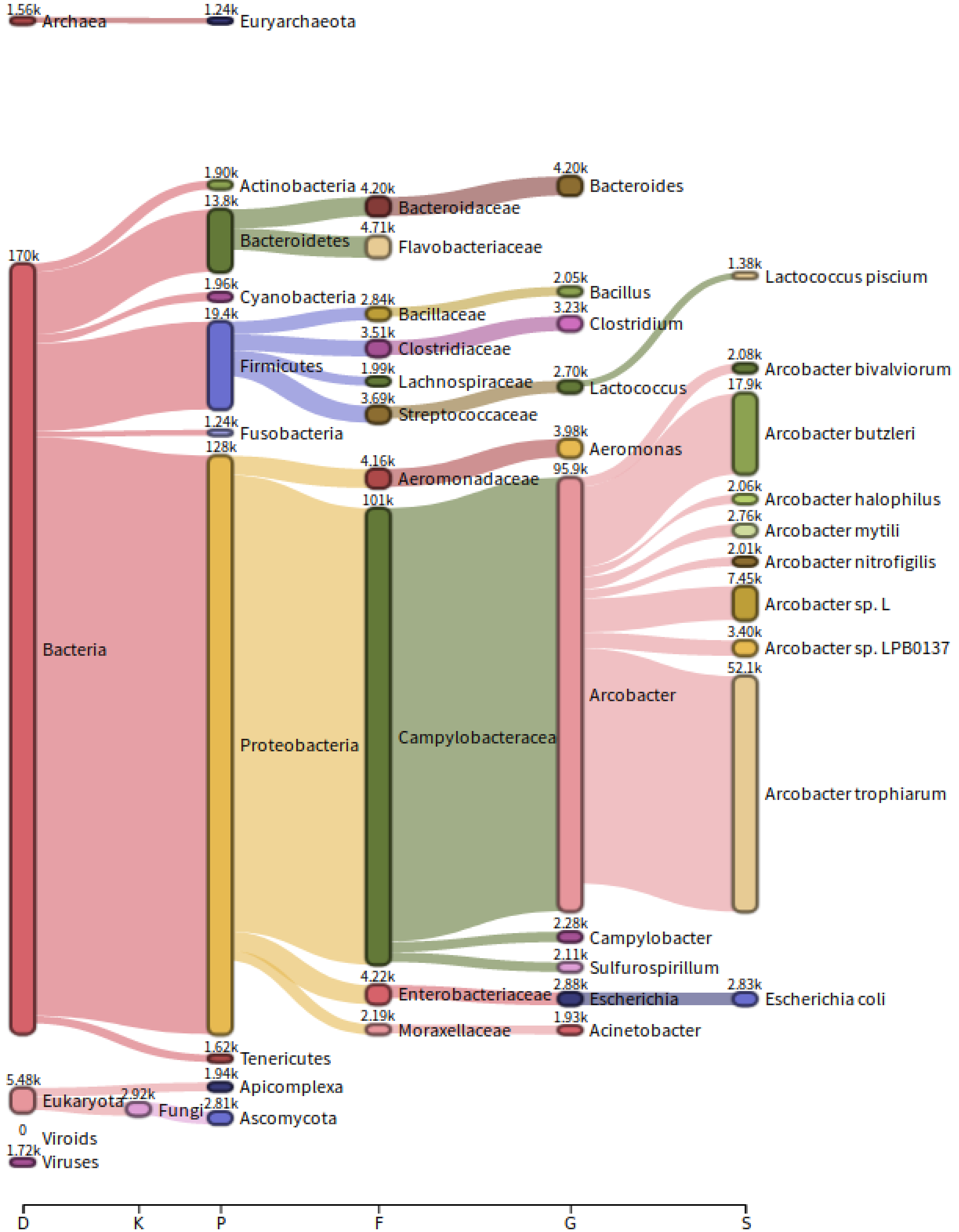

St Laurent

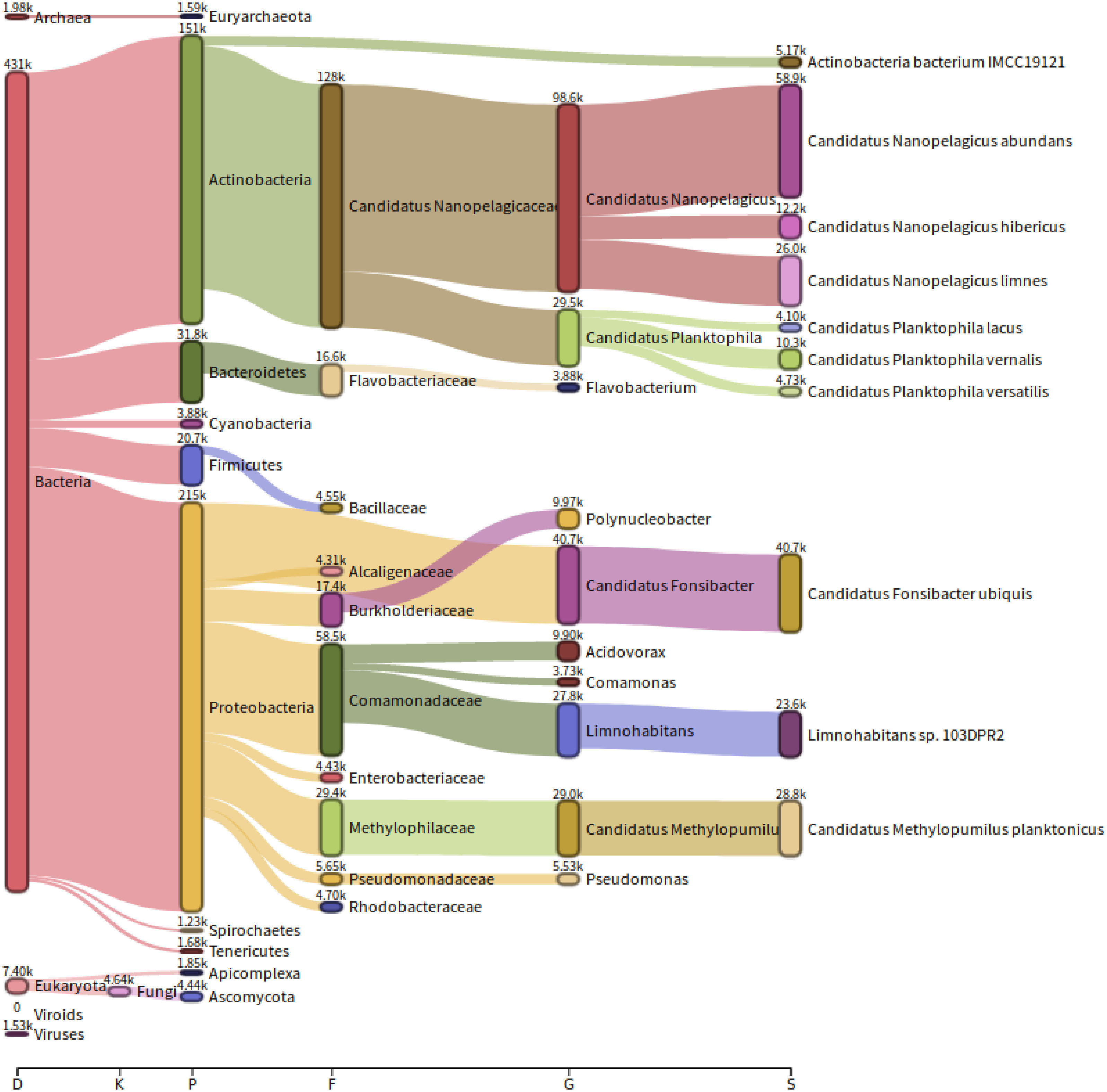
1.98k Archaea
1.59k Euryarchaeota
151k
431k
5.17k Actinobacteria bacterium IMCC19121
58.9k
128k
98.6k
Candidatus Nanopelagicus abundans
Actinobacteria
Candidatus Nanopelagicaceae
Candidatus Nanopelagicus
12.2k Candidatus Nanopelagicus hibericus
26.0k Candidatus Nanopelagicus limnes
29.5k
4.10k Candidatus Planktophila lacus
Candidatus Planktophila
31.8k
10.3k Candidatus Planktophila vernalis
16.6k
Bacteroidetes
Flavobacteriaceae
3.88k Flavobacterium
4.73k Candidatus Planktophila versatilis
3.88k Cyanobacteria
20.7k Firmicutes
Bacteria
215k
4.55k Bacillaceae
9.97k Polynucleobacter
40.7k
40.7k
4.31k Alcaligenaceae
Candidatus Fonsibacter
Candidatus Fonsibacter ubiquis
17.4k Burkholderiaceae
58.5k
9.90k Acidovorax
3.73k Comamonas
Comamonadaceae
27.8k
23.6k
Proteobacteria
Limnohabitans
Limnohabitans sp. 103DPR2
4.43k Enterobacteriaceae
29.4k
29.0k
28.8k
Methylophilaceae
Candidatus Methylopumilu
Candidatus Methylopumilus planktonicus
5.65k Pseudomonadaceae
5.53k Pseudomonas
4.70k Rhodobacteraceae
1.23k Spirochaetes
1.68k Tenericutes
7.40k
1.85k Apicomplexa
4.64k
Eukaryota
Fungi
4.44k Ascomycota
0 Viroids
1.53k Viruses
D
K
P
F
G
S

Sydhavnen

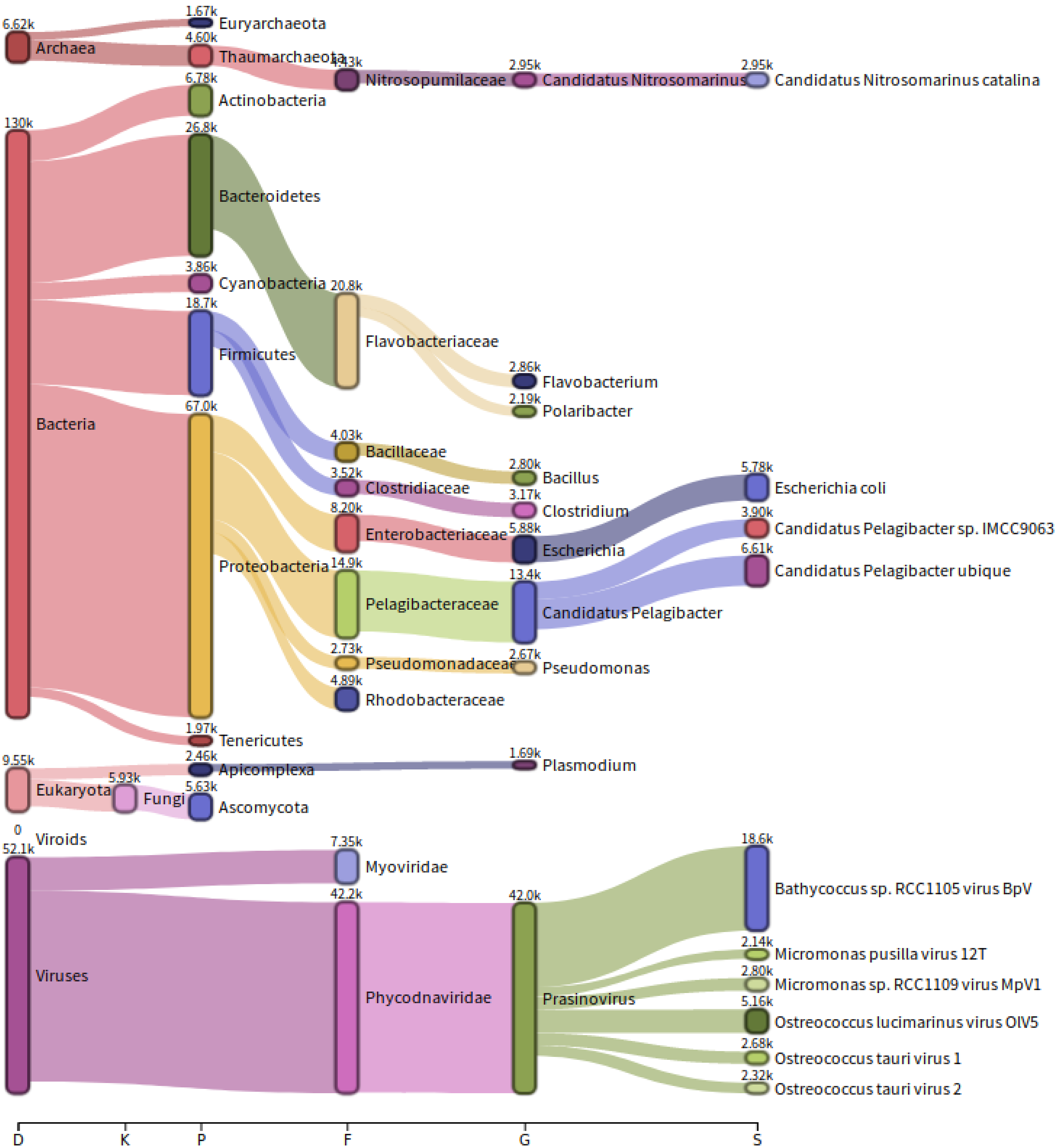

6.62k
Archaea
1.67k
Euryarchaeota
4.60k
Thaumarchaeota
4.43k
Nitrosopumilaceae
2.95k
Candidatus Nitrosomarinus
2.95k
Candidatus Nitrosomarinus catalina
6.78k
Actinobacteria
130k
Bacteria
26.8k
Bacteroidetes
3.86k
Cyanobacteria
20.8k
Flavobacteriaceae
18.7k
Firmicutes
2.86k
Flavobacterium
2.19k
Polaribacter
67.0k
Proteobacteria
4.03k
Bacillaceae
2.80k
Bacillus
3.52k
Clostridiaceae
3.17k
Clostridium
5.78k
Escherichia coli
8.20k
Enterobacteriaceae
5.88k
Escherichia
3.90k
Candidatus Pelagibacter sp. IMCC9063
6.61k
Candidatus Pelagibacter ubique
14.9k
Pelagibacteraceae
13.4k
Candidatus Pelagibacter
2.73k
Pseudomonadaceae
2.67k
Pseudomonas
4.89k
Rhodobacteraceae
1.97k
Tenericutes
9.55k
Eukaryota
2.46k
Apicomplexa
1.69k
Plasmodium
5.93k
Fungi
5.63k
Ascomycota
0
Viroids
52.1k
Viruses
7.35k
Myoviridae
42.2k
Phycodnaviridae
42.0k
Prasinovirus
18.6k
Bathycoccus sp. RCC1105 virus BpV
2.14k
Micromonas pusilla virus 12T
2.80k
Micromonas sp. RCC1109 virus MpV1
5.16k
Ostreococcus lucimarinus virus OlV5
2.68k
Ostreococcus tauri virus 1
2.32k
Ostreococcus tauri virus 2
D
K
P
F
G
S

Vedder Canal

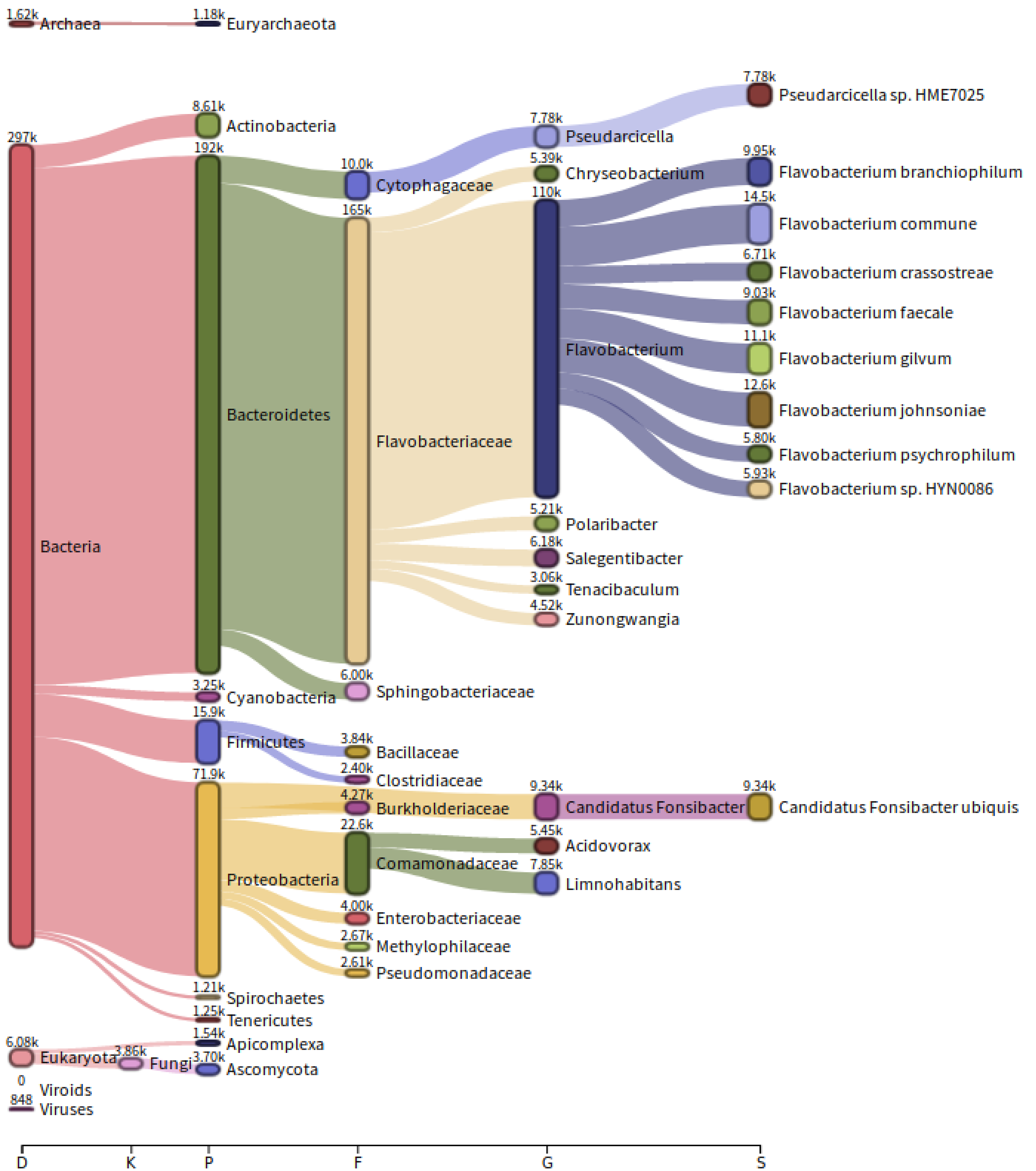

Vedder

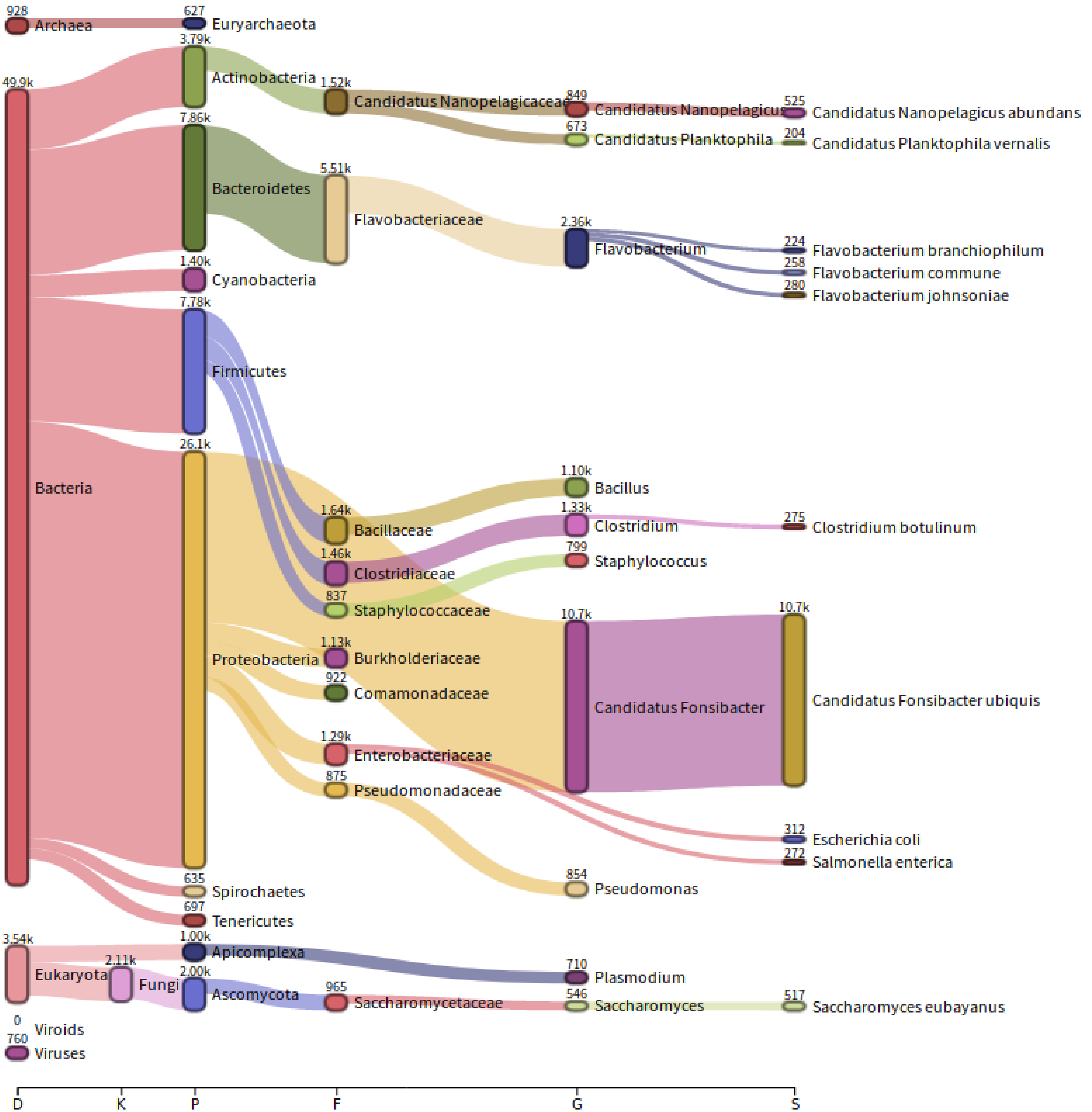

928 Archaea
627 Euryarchaeota
3.79k Actinobacteria
49.9k Bacteria
1.52k Candidatus Nanopelagicaceae
849 Candidatus Nanopelagicus
525 Candidatus Nanopelagicus abundans
673 Candidatus Planktophila
204 Candidatus Planktophila vernalis
7.86k Bacteroidetes
5.51k Flavobacteriaceae
2.36k Flavobacterium
224 Flavobacterium branchiophilum
258 Flavobacterium commune
280 Flavobacterium johnsoniae
1.40k Cyanobacteria
7.78k Firmicutes
26.1k Proteobacteria
1.10k Bacillus
1.64k Bacillaceae
1.33k Clostridium
275 Clostridium botulinum
1.46k Clostridiaceae
799 Staphylococcus
837 Staphylococcaceae
10.7k Candidatus Fonsibacter
10.7k Candidatus Fonsibacter ubiquis
1.13k Burkholderiaceae
922 Comamonadaceae
1.29k Enterobacteriaceae
875 Pseudomonadaceae
312 Escherichia coli
272 Salmonella enterica
854 Pseudomonas
635 Spirochaetes
697 Tenericutes
3.54k Eukaryota
1.00k Apicomplexa
2.11k Fungi
2.00k Ascomycota
710 Plasmodium
965 Saccharomycetaceae
546 Saccharomyces
517 Saccharomyces eubayanus
0 Viroids
760 Viruses
D K P F G S

Yare E

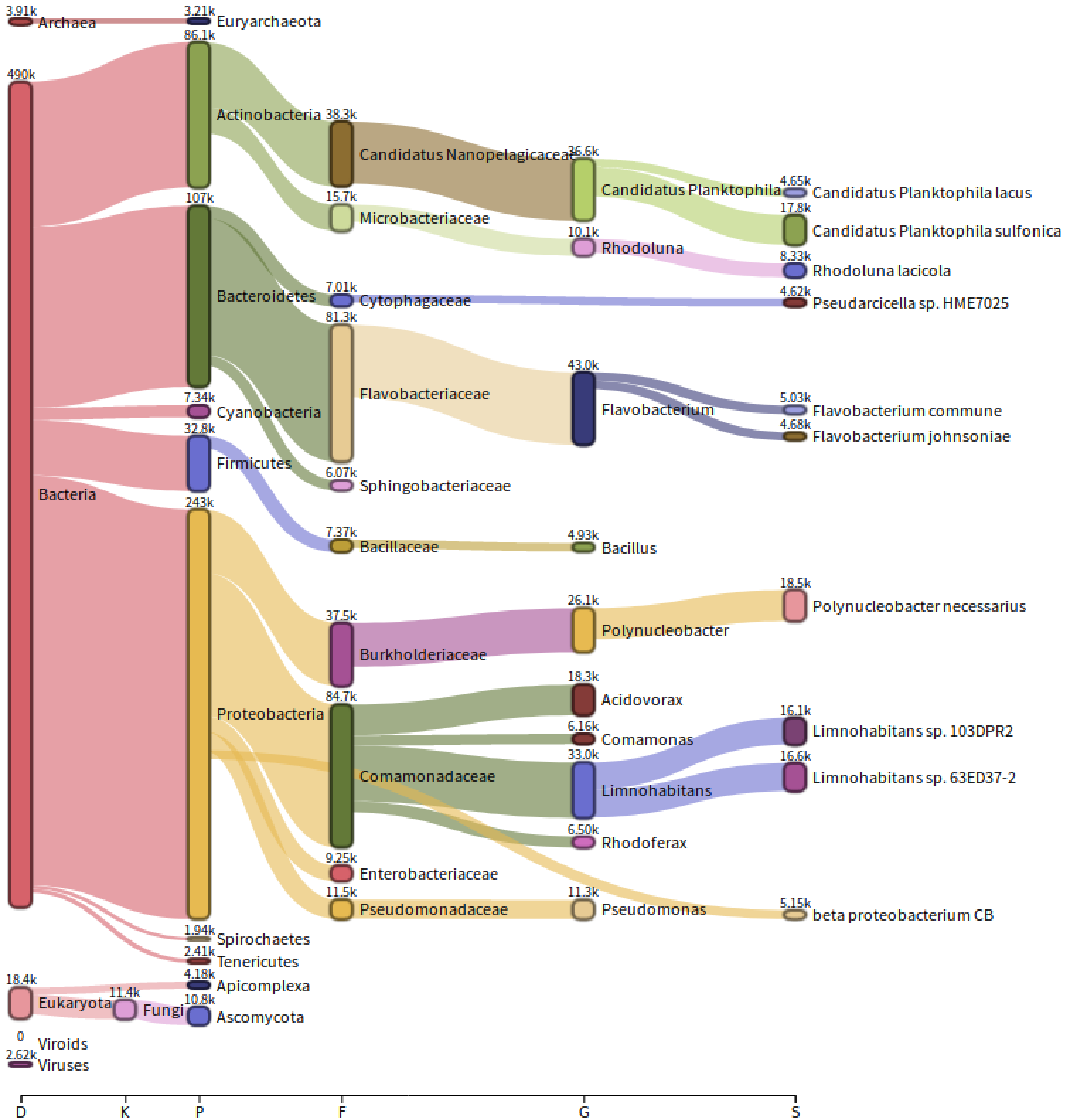
3.91k Archaea
3.21k Euryarchaeota
86.1k Actinobacteria
490k Bacteria
38.3k Candidatus Nanopelagicaceae
36.6k Candidatus Planktophila
4.65k Candidatus Planktophila lacus
17.8k Candidatus Planktophila sulfonica
15.7k Microbacteriaceae
10.1k Rhodoluna
8.33k Rhodoluna lacicola
107k Bacteroidetes
7.01k Cytophagaceae
4.62k Pseudarcicella sp. HME7025
81.3k Flavobacteriaceae
43.0k Flavobacterium
5.03k Flavobacterium commune
4.68k Flavobacterium johnsoniae
7.34k Cyanobacteria
32.8k Firmicutes
6.07k Sphingobacteriaceae
243k Proteobacteria
7.37k Bacillaceae
4.93k Bacillus
18.5k Polynucleobacter necessarius
26.1k Polynucleobacter
37.5k Burkholderiaceae
18.3k Acidovorax
84.7k Comamonadaceae
6.16k Comamonas
16.1k Limnohabitans sp. 103DPR2
33.0k Limnohabitans
16.6k Limnohabitans sp. 63ED37-2
6.50k Rhodoferax
9.25k Enterobacteriaceae
11.5k Pseudomonadaceae
11.3k Pseudomonas
5.15k beta proteobacterium CB
1.94k Spirochaetes
2.41k Tenericutes
18.4k Eukaryota
11.4k Fungi
4.18k Apicomplexa
10.8k Ascomycota
0 Viroids
2.62k Viruses
D
K
P
F
G
S

Yare W

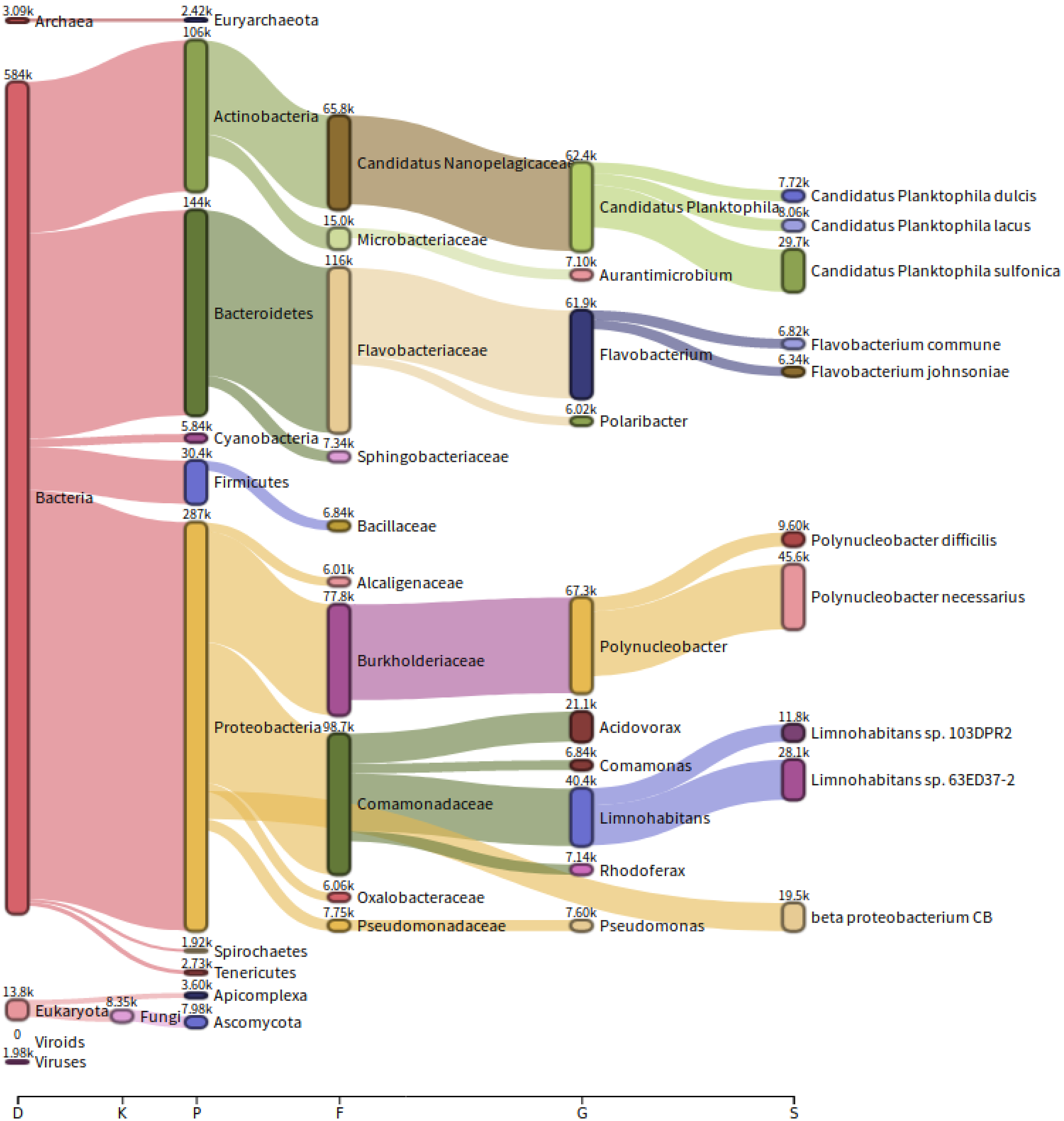
3.09k Archaea
2.42k Euryarchaeota
584k
Bacteria
106k Actinobacteria
144k Bacteroidetes
5.84k Cyanobacteria
30.4k Firmicutes
287k Proteobacteria
1.92k Spirochaetes
2.73k Tenericutes
13.8k Eukaryota
8.35k Fungi
3.60k Apicomplexa
7.98k Ascomycota
0 Viroids
1.98k Viruses
65.8k Candidatus Nanopelagicaceae
15.0k Microbacteriaceae
116k Flavobacteriaceae
7.34k Sphingobacteriaceae
6.84k Bacillaceae
6.01k Alcaligenaceae
77.8k Burkholderiaceae
98.7k Comamonadaceae
6.06k Oxalobacteraceae
7.75k Pseudomonadaceae
62.4k Candidatus Planktophila
7.10k Aurantimicrobium
61.9k Flavobacterium
6.02k Polaribacter
67.3k Polynucleobacter
21.1k Acidovorax
6.84k Comamonas
40.4k Limnohabitans
7.14k Rhodoferax
7.60k Pseudomonas
7.72k Candidatus Planktophila dulcis
8.06k Candidatus Planktophila lacus
29.7k Candidatus Planktophila sulfonica
6.82k Flavobacterium commune
6.34k Flavobacterium johnsoniae
9.60k Polynucleobacter difficilis
45.6k Polynucleobacter necessarius
11.8k Limnohabitans sp. 103DPR2
28.1k Limnohabitans sp. 63ED37-2
19.5k beta proteobacterium CB
D
K
P
F
G
S
