# Supplementary material for: Metagenomic analysis of planktonic riverine microbial consortia using nanopore sequencing reveals insight into river microbe taxonomy and function
Source: Gigascience. 2020 Jun 10;9(6):giaa053. doi: 10.1093/gigascience/giaa053 (PMC7285869; doi:10.1093/gigascience/giaa053)
Supplement: giaa053_Supplemental_Files [file giaa053_supplemental_files.zip › GIGA-S-19-00534-3_Supplementals.docx]

Supplemental Figure 1: Read length distribution plots for sample (A and B) and negative (C and D) sequencing libraries. The cumulative sequenced bases plots (A and C) allow read length percentiles to be identified; read N10, N50, and N90 are indicated on the plot by vertical lines and a pair of circles. The digital electrophoresis plots (B and D) show the distribution of read lengths in the libraries, as might be seen via gel electrophoresis. The sample libraries generally show a very tight read length distribution (except for the low-count samples, Karori and Neckar), whereas the negative samples have platykurtic length distribution curves. Not shown in panel D: James-neg (because only one sequence, *H. sapiens*, ATP synthase, was detected, 1.67 kbp).


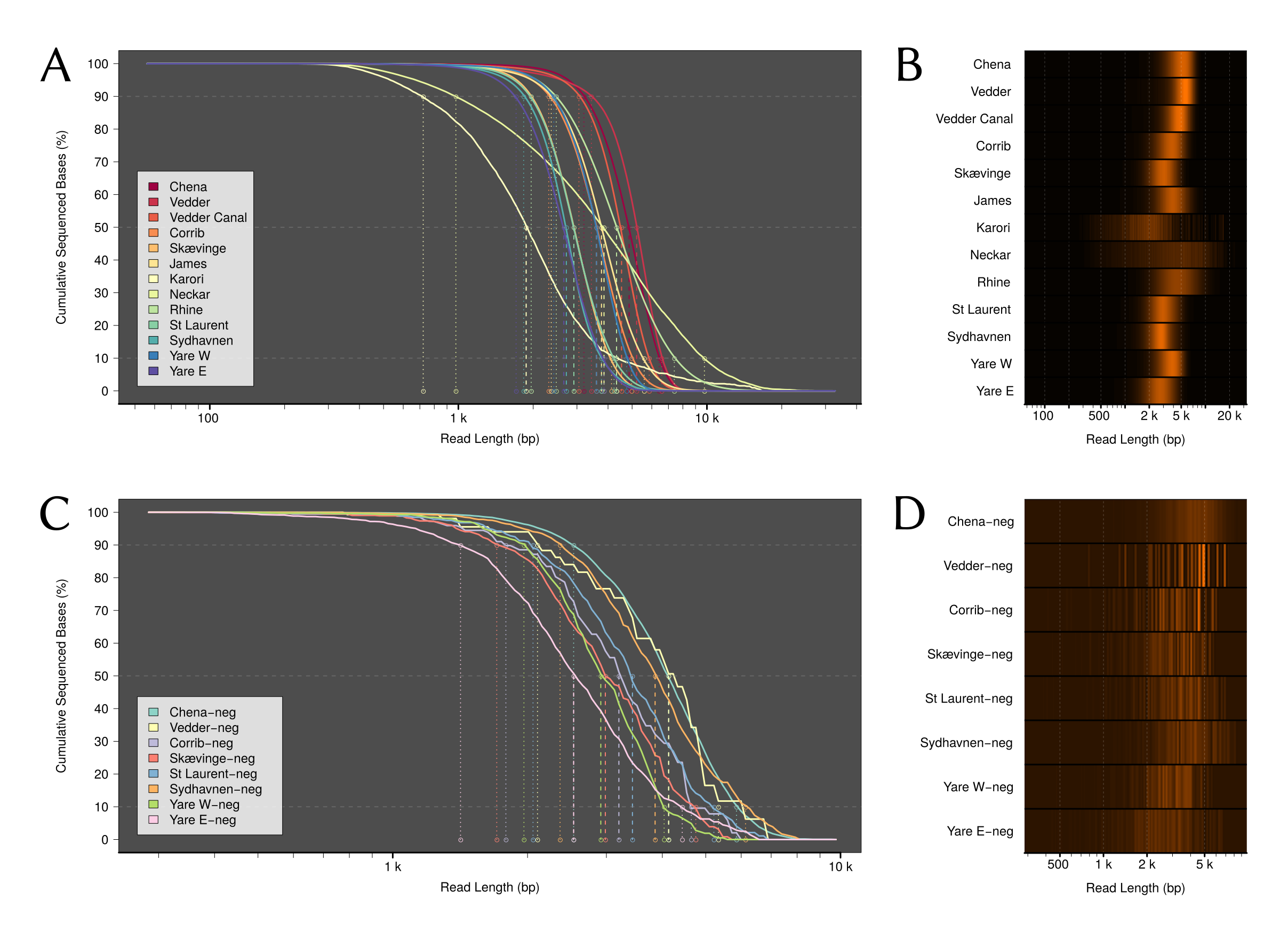


Supplemental Figure 2. Proportion of GC in each of 13 metagenomes from 11 rivers. Color scale same as shown in Supplemental Figure 1.


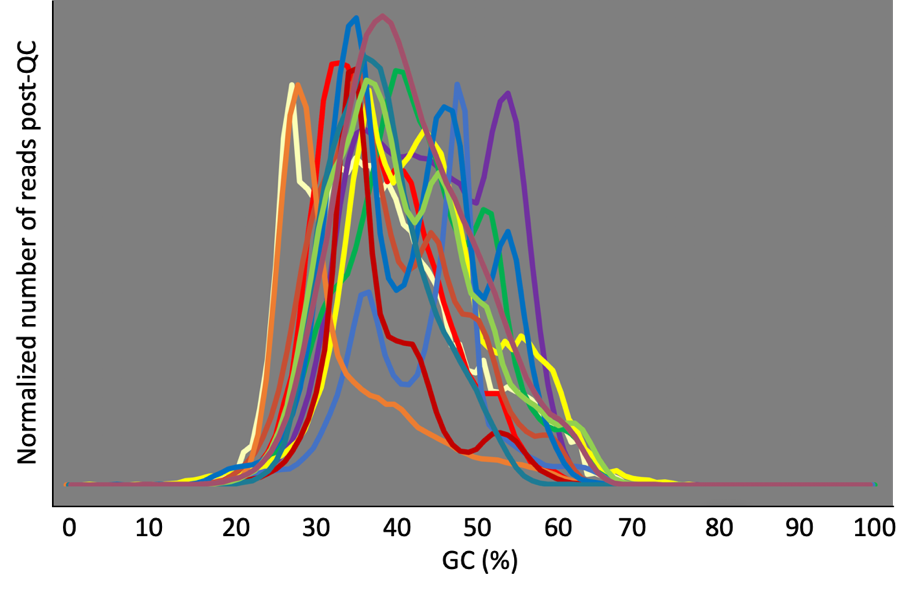


Supplemental Figure 3. Representative taxonomy shown as Pavian plots for each of 13 metagenomes from 11 rivers.

Please see filename “GIGA-S-19-00534-3_pavian.pdf” for all 13 plots.

Supplemental Figure 4. Full-resolution location maps (insets are reduced-resolution Pavian plots) to illustrate the range of sites sampled and the relative diversity of taxa identified in 13 riverine metagenomes.

Supplemental Table 1. Long-read metagenome properties and downstream analysis summary. L50: length of the shortest read in the set of the longest 50% of base-called data, N50: number of reads in the set of the longest 50% of base-called data, rRNA: number of reads that contain ribosomal RNA genes, Features: predicted proteins, CDS: identified proteins, Subsystems: number of reads assigned to all Subsystem level 1 functional categories, Anthro: percentage of reads with predicted protein functions annotated to Virulence, Disease and Defense, Phages, Prophages, Transposable elements, Plasmids, Metabolism of Aromatic Compounds, and Stress Response, α-Diversity: estimated from the distribution of the species-level annotations.

| **River** | **Reads** | **Bases (Gbp)** | **L50 (N50)** | **rRNA** | **Features** | **CDS** | **Subsystem reads** | **% Anthro** | **α-Diversity** |
| --- | --- | --- | --- | --- | --- | --- | --- | --- | --- |
| River Yare W | 1.2⋅10^6^ | 3.8 | 3.6 kb (4.5⋅10^5^) | 4799 | 4.3⋅10^6^ | 7.5x10^5^ | 1.7⋅10^6^ | 6.6 | 296 |
| River Yare E | 1.8⋅10^6^ | 4.4 | 2.6 kb (6.7⋅10^5^) | 648,702 | 5.1⋅10^6^ | 6.5⋅10^5^ | 1.4⋅10^6^ | 7.5 | 443 |
| Rhine River | 3.2⋅10^6^ | 12.6 | 4.3 kb (1.1⋅10^6^) | 13,412 | 13.9⋅10^6^ | 1.8⋅10^6^ | 4.1⋅10^6^ | 6.6 | 492 |
| Neckar River | 4.2⋅10^4^ | 0.1 | 3.8 kb (7.4⋅10^3^) | 203 | 1.1⋅10^5^ | 2.5⋅10^4^ | 5.5⋅10^5^ | 7.1 | 316 |
| River Corrib | 1.5⋅10^6^ | 4.8 | 3.6 kb (5.4⋅10^5^) | 4,467 | 5.6⋅10^6^ | 6.8⋅10^5^ | 1.5⋅10^6^ | 6.4 | 413 |
| Sydhavnen | 7.6⋅10^5^ | 1.9 | 2.7 kb (2.9⋅10^5^) | 1,712 | 2.3⋅10^6^ | 3.0⋅10^5^ | 3.9⋅10^5^ | 9.5 | 156 |
| Skævinge WWTP | 3.9⋅10^5^ | 1.0 | 2.9 kb (1.5⋅10^5^) | 1,436 | 1.4⋅10^6^ | 1.6⋅10^5^ | 2.6⋅10^5^ | 7.3 | 218 |
| James River | 2.2⋅10^5^ | 0.8 | 3.8 kb (8.0⋅10^5^) | 627 | 8.14⋅10^5^ | 1.4⋅10^5^ | 3.4⋅10^5^ | 6.0 | 452 |
| Chena River | 2.6⋅10^6^ | 11.4 | 4.8 kb (9.8⋅10^5^) | 4,242 | 12.4⋅10^6^ | 8.9⋅10^5^ | 1.8⋅10^6^ | 7.1 | 1139 |
| Vedder River | 1.6⋅10^5^ | 0.7 | 5.2 kb (5.9⋅10^4^) | 430 | 7.8⋅10^5^ | 7.3⋅10^5^ | 1.5⋅10^5^ | 18.9 | 486 |
| Vedder Canal | 5.2⋅10^5^ | 2.1 | 4.5 kb (2.0⋅10^5^) | 640,261 | 2.2⋅10^6^ | 6.4⋅10^5^ | 1.3⋅10^6^ | 6.2 | 135 |
| St. Laurent River | 8.8⋅10^5^ | 2.4 | 2.9 kb (3.3⋅10^5^) | 2,561 | 2.8⋅10^6^ | 5.8⋅10^5^ | 1.3⋅10^6^ | 6.1 | 480 |
| Karori Stream | 7.0⋅10^4^ | 0.01 | 1.9 kb (1.7⋅10^3^) | 27 | 1.4⋅10^4^ | 3.2⋅10^2^ | 4.8⋅10^2^ | 7.2 | 178 |

Supplemental Table 2. Site, study, and sample information. The notation “NA” in parentheses is included where negative read set files included too few reads to be analyzed in MG-RAST. Where accessions are given in parentheses, those refer to the barcoded negative control data. All FASTQ files are available from ENA via accessions numbers PRJEB34137 and ERP116996, individual indexing is in progress as of 30 August 2019.

| **River Name** | **Lat Long** | **Date** | **Lab ID** | **URID** | **Region** | **ENA** | **MG-RAST (negative)** |
| --- | --- | --- | --- | --- | --- | --- | --- |
| River Yare W | [52.620751, 1.229554](https://www.openstreetmap.org/?mlat=52.620751&mlon=1.229554#map=15/52.620751/1.229554) | 2017-04-24 | 4 | Yare W | Norwich UK | TBD | [mgl735606](https://www.mg-rast.org/mgmain.html?mgpage=library&library=mgl735606) ([mgl735602](https://www.mg-rast.org/mgmain.html?mgpage=library&library=mgl735602)) |
| River Yare E | [52.6054, 1.3825](https://www.openstreetmap.org/?mlat=52.6054&mlon=1.3825#map=15/52.6054/1.3825) | 2017-10-11 | 9 | Yare E | Norwich UK | TBD | [mgl735614](https://www.mg-rast.org/mgmain.html?mgpage=library&library=mgl735614) ([mgl735603](https://www.mg-rast.org/mgmain.html?mgpage=library&library=mgl735603)) |
| Rhine River | [51.861409, 6.068399](https://www.openstreetmap.org/?mlat=51.861409&mlon=6.068399#map=15/51.861409/6.068399) | 2017-07-05 | 5 | Rhine | Bimmen NL/DE | TBD | [mgl735607](https://www.mg-rast.org/mgmain.html?mgpage=library&library=mgl735607) (NA) |
| Neckar River | [48.516222, 9.04575](https://www.openstreetmap.org/?mlat=48.516222&mlon=9.04575#map=15/48.516222/9.04575) | 2017-06-26 | 12 | Neckar | Tübingen DE | TBD | [mgl735617](https://www.mg-rast.org/mgmain.html?mgpage=library&library=mgl735617) (NA) |
| River Corrib | [53.281242, -9.059482](https://www.openstreetmap.org/?mlat=53.281242&mlon=-9.059482#map=15/53.281242/-9.059482) | 2018-02-05 | 13 | Corrib | Galway IE | TBD | [mgl735618](https://www.mg-rast.org/mgmain.html?mgpage=library&library=mgl735618) (NA) |
| Sydhavnen | [55.662192, 12.564093](https://www.openstreetmap.org/?mlat=55.662192&mlon=12.564093#map=15/55.662192/12.564093) | 2018-03-17 | 14 | Sydhavnen | København DK | TBD | [mgl735619](https://www.mg-rast.org/mgmain.html?mgpage=library&library=mgl735619) ([mgl735604](https://www.mg-rast.org/mgmain.html?mgpage=library&library=mgl735604)) |
| Skævinge WWTP | [55.90583, 12.12806](https://www.openstreetmap.org/?mlat=55.90583&mlon=12.12806#map=15/55.90583/12.12806) | 2017-05-30 | 17 | Skævinge WWTP | Skaevinge DK | TBD | [mgl735623](https://www.mg-rast.org/mgmain.html?mgpage=library&library=mgl735623) (NA) |
| James River | [37.527941, -77.436144](https://www.openstreetmap.org/?mlat=37.527941&mlon=-77.436144#map=15/37.527941/-77.436144) | 2017-06-13 | 15 | James | Richmond USA | TBD | [mgl735622](https://www.mg-rast.org/mgmain.html?mgpage=library&library=mgl735622) (NA) |
| Chena River | [64.79583, -147.19361](https://www.openstreetmap.org/?mlat=64.79583&mlon=-147.19361#map=15/64.79583/-147.19361) | 2018-10-26 | 18 | Chena | Fairbanks USA | TBD | [mgl735626](https://www.mg-rast.org/mgmain.html?mgpage=library&library=mgl735626) ([mgl735605](https://www.mg-rast.org/mgmain.html?mgpage=library&library=mgl735605)) |
| Vedder River | [49.0973, -121.9858](https://www.openstreetmap.org/?mlat=49.0973&mlon=-121.9858#map=15/49.0973/-121.9858) | 2017-08-28 | 6/A | Vedder | Vancouver CA | TBD | [mgl735613](http://www.mg-rast.org/mgmain.html?mgpage=library&library=mgl735613) (NA) |
| Vedder Canal | [49.1052, -122.079](https://www.openstreetmap.org/?mlat=49.1052&mlon=-122.079#map=15/49.1052/-122.079) | 2017-08-28 | 6/B | Vedder Canal | Vancouver CA | TBD | [mgl735610](https://www.mg-rast.org/mgmain.html?mgpage=library&library=mgl735610) (NA) |
| St. Laurent River | [45.50609, -73.53414](https://www.openstreetmap.org/?mlat=45.50609&mlon=-73.53414#map=15/45.50609/-73.53414) | 2017-10-13 | 11 | St Laurent | Montreal CA | TBD | [mgl735616](https://www.mg-rast.org/mgmain.html?mgpage=library&library=mgl735616) (NA) |
| Karori Stream | [-41.29699, 174.72226](https://www.openstreetmap.org/?mlat=-41.29699&mlon=174.72226#map=15/-41.29699/174.72226) | 2017-11-01 | 10 | Karori | Wellington NZ | TBD | [mgl735615](https://www.mg-rast.org/mgmain.html?mgpage=library&library=mgl735615) (NA) |
