# Supplementary material for: Metagenomic analysis of planktonic riverine microbial consortia using nanopore sequencing reveals insight into river microbe taxonomy and function
Source: Gigascience. 2020 Jun 10;9(6):giaa053. doi: 10.1093/gigascience/giaa053 (PMC7285869; doi:10.1093/gigascience/giaa053)
Supplement: giaa053_Response_to_Reviewer_Comments_Original_Submission [file giaa053_response_to_reviewer_comments_original_submission.pdf]

Author's Response To Reviewer Comments

Close

Reviewer #1
Reddington et al have produced a manuscript describing the application of Oxford Nanopore MinION metagenomic sequencing to describe the genetic taxonomic and functional potential of river water collected from 11 rivers sampled worldwide.

1. It is difficult to assess this study, as in many ways it is a methods paper, except that it lacks a critical comparison with the current standard approach that is used for metagenomics, Illumina short read sequencing. As such, the study lacks a rationale, other than to show that environmental metagenomic data can be collected using the minION sequencing platform (which has been done previously, although not admittedly for river samples). In addition, no contextual physicochemical data was collected from each river, so there is limited ecological or biogeographical inferences that can be made. Without high quality short read data as a comparison, it is hard to assess the strengths and weaknesses of this approach.

Response: We have now cited on p.4 the results of comparison of MinION with short-read data. Accuracy has been addressed through several prior studies (including two papers from the MARC consortium published in F1000Research, https://doi.org/10.12688/f1000research.7201.1 and https://doi.org/10.12688/f1000research.11354.1). We did not perform accuracy analysis in our study because such analysis assumes that any given read is aligned to its correct reference. In metagenomic samples, this is a difficult assumption to make. We assessed read quality based on previous studies, since there has been enough nanopore work across a variety of samples (bacteria, plants, animals, human, etc.) to provide a reproducible measure of read quality. We took that a step further to provide shotgun WGS examples, using samples filtered in a way to eliminate most eukaryote and to retain most picoplankton. While not a comparison with Illumina-based approaches, our study demonstrates that environmental sample sequencing can be performed in-field using a simple approach and yields meaningful information. We agree with the reviewer in that follow up studies could further investigate the biogeographical inferences that could be made from such data.

2. Useful questions could address the utility of having longer reads generated by Nanopore sequencing. For example, does this result in a higher proportion of reads being functionally and taxonomically assigned? How does the lower read quality influence assignments? Do the longer reads assist with assembly based approaches? Although short read data may not be available as a comparison, a critical assessment of the strengths and weaknesses of this approach would improve the manuscript.

Response: Our efforts focused first on assignments at the domain level. To properly assign functional information would require a highly curated reference for comparison, along with a carefully controlled mock set for testing. Metagenomic classifications are particularly challenging due to the inherent biological diversity and complexity of the samples. We agree with the reviewer that it would be a great to compare these data with Illumina data, but argue that this would belong to a separate study. To address the issue superficially as this was not the focus of the current study, we have revised the introduction to include references to several prior studies in this area (p.4).

3. The other main issue is the focus on taxonomic assignment using metagenomic reads. Although this approach has been shown to work well in environments that are extremely well represented by reference genomes (for example, the human gut), it invariably does not work well in less well studied (and more diverse) environments (see https://environmentalmicrobiome.biomedcentral.com/articles/10.1186/s40793-019-0347-1 for an example of this with short read data). The taxonomic saturation of the rarefaction curves in figure 2 is likely a reflection of this.

Response: The rarefaction curves didn’t level off, likely because of our WGS approach which yielded < 1× coverage. We now have included attention to this issue on p 5-6 of the ms. Interestingly, the paper recommended above also describes searching for AMR genes specifically in short-read data. We used our

long-read data to look for AMR genes and also found them, even at the much lower sequencing depth. This is now covered in the ms on p. 13.

4. It would be interesting to compare metagenomic taxonomic assignment (via Kraken2) with taxonomy assignment using 16S rRNA genes extracted from the metagenomes.

Response: In response to this suggestion, we performed the recommended investigation. We found that 16S sequences extracted from the metagenomic data are not sufficient in number to represent the breath of the community identified by metagenomics, likely because we would need at least 1× coverage of all taxa, which admittedly we did not. In summary, we performed analysis but as it was inconclusive due to insufficient depth, we have not included it in the revised ms.
5. The manuscript is on the whole well written, and I only have a few small comments below regarding the text. However, I think the plots and data visualisation could with improving:
1. Figure 1 does not need to be in the main manuscript and could be moved to supplementary.
Response: Thanks for the suggestion; we moved Figure 1 into the Supplementary Figures
2. Figure 5 is unreadable and needs a re-think. My preference would be to include site maps in the supplementary figures, and produce a bar chart (or series of bar charts and different taxonomic levels) highlighting the taxonomic differences in the samples.
Response: In response this suggestion, we have created two additional Supplementary Figures to address this: 1) full-resolution pavian plots with river names underneath; 2) full-resolution location maps with insets consisting of reduced-resolution pavian plots
3. Tables 1 and 2, could be moved to supplementary.
Response: Thanks for the suggestion; we have moved Tables 1 and 2 into Supplementary Tables
4. Tables 3 - 5, I recommend re-producing these as heatmaps, which would make them far easier to interpret.
Response: Thanks for the suggestion; these have been amended with color, similar to heatmaps

Minor comments:
L169-170; All these families have representatives commonly found in freshwater.
L227; Picoplankton includes eukaryotes.

Reviewer #2
1. Reddington et al. present their work on using long read-based, whole genome sequencing of river-derived microbial consortia to study river ecology. This represents a timely and important work as it demonstrates the possibility of on-site sequencing for environmental monitoring. While the on-site data processing may (still) be challenging, e.g., due to very large reference databases, there might be ways to overcome this challenge, e.g., tailored databases or simply more powerful compute. Hence, being able to monitor not only the physico-chemical composition but also microbial composition and functional potential allows greater resolution of water quality, e.g., assessing the presence and abundance of relevant microorganisms, such as specific algae or specific bacteria. While Reddington et al. followed a homology-based approach to resolve taxonomy and function, I would have found it nice to see functions linked to taxonomy more strongly. This is something I would expect long read-based sequencing to be of particular use for, especially given that many of the taxa are lowly abundant. Having potentially several genes in synteny on an actual sequencing read avoids the need to perform de novo assembly, which is likely challenging given the dominantly lowly abundant taxa and thus limited sequencing coverage. However, I do not consider this a major limitation.

Response: As we noted in our response to Reviewer # 1 above, functional classification is complicated in metagenomic samples due to biological complexity. A better classification, that links at the functional level, would require a carefully curated and comprehensive reference set for comparison. Such analysis also would require a set of controlled experiments for establishing a baseline for functional classification and taxonomic assignments. Another reason that we chose MG-RAST was because of its linkage to functional prediction. We cite references for these types of consideration on p. 3-4, more detail of the database matches on p. 12, and the methods are clarified in the ms on p.23.

2. In general, I found the manuscript well to read, but encourage the authors to consider rearranging and/or reformulate specific parts to improve the readability further, as described in my Overall comments and Detailed comments. I think my comments should be straight-forward to be addressed by the authors as there are no additional experiments requested or the like, but mostly clarifications on individual aspects.

Overall comments

- The structure of the manuscript could be improved. Please see also my comment on the position of the text on negative controls.

- It is nice to see that the authors extended their analyses beyond taxonomy and also study the functional potential. This is very relevant as similar functions may be encoded by distinct taxa or horizontal gene transfer events may occur or have occured affecting the phenotype. However, given that the vast majority of organisms was found in very low abundance (< 1%), in how much does the proposed approach improve over amplicon-based characterization?

Response: We have revised the ms to include references showing that the long-read nanopore approach is comparable with amplicon-based approaches (p. 4).

3. If only fractions of the majority of genomes are expected to be recovered, why perform whole-genome sequencing in the first place? If conclusions are drawn from the taxonomic assignments of the reads and not by population-resolved inference of the functional potential (e.g., via metagenome-assembled genomes), this can be more cheaply achieved using amplicon-based sequencing.

Response: This reviewer's comment has highlighted an extremely important distinction about metagenomic approaches, namely that an amplicon study could detect rare components but conversely the choice of amplicon (e.g., 16S) could lead to missing other extremely important components, even if they are highly represented (i.e., the viral), or of health consequence (i.e., certain eukaryote components). One option in metagenomics is to employ multi-metabarcoding with multiple universal primer sets (16S, archaea, 28S, COI) but such combined tests aren't yet optimized. We are in hopes that the revisions included in the introduction (op cit) are adequate in this regard.

4. Not that I am advocating for amplicon-based sequencing in general. Quite the opposite, i.e., I would like to encourage the authors to highlight the added value of on-site _whole-genome_ sequencing more strongly, e.g., to resolve gene synteny directly from sequencing reads (rather than contigs) and/or to avoid primer bias or multiple sample processing to account for different "fractions" (16S, 18S, viral, etc.).

Response: This suggestion prompted us to add additional discussion on p. 14.

- While I found the ecological analyses limited, i.e., my main take-away was that anthropogenic factors may have a substantial effect on the community composition and presence of AMR-related genes - but there is likely much more than that in terms of ecology -, I understand that the focus of this study was on demonstrating the feasibility and usefulness of on-site sequencing, specifically using MinION-based sequencing. Hence, I am not sure if putting such a strong focus on the river ecology in the title is a good idea as other readers might have respective expectations. It is not a major comment though, but maybe the authors could think of a respectively adjusted title.

Response: We appreciate and understand this concern and in response revised the title to reduce the expectation that there was a big focus on ecology.

- I would encourage the authors to keep discussion points for the Discussion. This would reduce redundancy during reading.

Detailed comments
L85-86: I think this could be even emphasized "and AMR genes, thus highlighting the importance/need to include microbial community data in broad-scale ecosystem models".
Response: Done, p.3

L89-90: Please include a respective reference or support this by the respective results.
Response: Done, p.3

L91-92: While I agree that field deployable sequencing is important, I am not sure if it is necessary or convenient. I would welcome further arguments to strenghten this.
We accept this challenge and have added some remote system real-time examples on p.3 where just such analysis would be helpful, and added a reference where another group has used the technology for on-site species ID.
L94: How about replacing "rapid" by "on-site"? "Rapid" is a rather relative term after all.
Response: Done, p.4

L98-100: Please clarify what particular advantage long-read data would bring. Importantly, I am not saying that this is not the case, but the authors could probably provide respective references easily to support their argument. Please also see my Overall comments.
Response: Excellent point. We have added several references and examples on p. 4

L103-104: "To evaluate proof-of-concept" there seems to be something missing here. Maybe "a proof-of-concept"? For what? I assume field deployable sequencing?
Response: The term has been eliminated completely in the revised ms in lieu of the examples mentioned above.

L115: I find it unexpected that the Figure references start with "B&D", later followed by "A&C". Moreover, Table 2 comes before Table 1 was referred to. Albeit, Table 1 is referred to in the Methods, so this may depend on the particular specifications of the journal. This is clearly minor, but hopefully also easy to fix for the authors in the proofs, if the manuscript is accepted for publication.
Response: We moved Fig 1 to Supplemental Figures and renumbered Figures and Tables throughout

L116-117: Maybe write "of total/cumulative length" or something along those lines?
Response: Replaced with “data sets averaging 1.1·106 reads (3.8·109 bases) of read length 3.4·103 bp” on p.5

L117: Maybe write "Rarefaction of the metagenomic data" or something along those lines?
Response: Replaced with “Metagenomic results rarefaction” on p. 5

L118: I highly welcome the inclusion of negative controls by the authors. However, I was confused by finding an elaboration on this important aspect only starting at L287. I would thus encourage the authors to move this aspect to near the beginning of the results as this is relevant when reading further. For example, I initially wondered whether the pronounced diversity in the "Chena" and "Corrib" samples might potentially partially be explained by contaminations which might have a disproportionally larger effect in low-biomass samples (e.g., river water).
Response: We appreciate the suggestion and moved this up to the second section under Analyses, now on p. 5.

L124-125: Please support this statement with respective results, e.g., a GC-distribution plot for the readers to better appreciate the extent of multimodality, etc.
Response: We have added a supplemental figure to illustrate the GC multimodality per this suggestion, p. 6.

L128: I found this to be confusing. The authors say that the majority of reads were of bacterial origin, but write a few sentences prior that One Codex was discarded due to a suspected deficiency in eukaryotic genomes in the underlying database? I am not advocating necessarily for the use of One Codex, but rather want to highlight that this formulation is confusing and should be revised.
Response: In response to this suggestion, we reworded and added ranges on the middle of p. 6. To be clear, One Codex leaves “a portion” of reads unclassified (hugely variable depending on the river), whereas due to the larger database and also to a different algorithm, MG-RAST classified all reads for all samples.

L143-144: I would suggest removing "A final group pf prokaryotes".
Response: Done, p. 7.

L147: Why not use "archaea" instead of "archaebacterial". Sticking with the former would be more consistent.
Response: Excellent suggestion because it’s simpler, p. 7.

L151: I am not a native English speaker, so I had to look up "promulgator". It is referred to as "publisher". It is thus not clear to me how this word fits in this context. I would suggest using an alternative term to make it more accessible to non-native English readers.
Response: We like this suggestion, appreciate the different perspective, and have substituted “player in” on p. 7.

L192: While Campylobacter, Clostridium, and Prevotella include species which are human pathogens, referring to these genera as pathogens is problematic in my opinion as it generalizes too much. If

possible, I would encourage the authors to tone this somewhat done, as I do not see in how much this is necessary to be highlighted in their work. The text about "anthropogenically relevant" in the following is fair and informative, so should stay.
Response: This suggestion is excellent. We reworded this part of the report so as not to imply that all of these members are pathogens or problems. See p. 9, last paragraph.

L204: Not sure what the authors mean by "The most common observation for 11 samples (virus accounting for <1% of reads) is typical of other river planktonic metagenomes". If this refers to the fact that virus reads are typically < 1%, how about replacing the parentheses by, "i.e.,"?
Response: This section has been reworded in a similar way to what is suggested, p. 10.

L211-212: How about using "... were virus-derived" or "were virus-borne"?
Response: In the process of rewording this section per the above suggestion, this phrase no longer occurs.

L212: What does "the ther" mean? I suspect it is a typo?
Response: Yes, thanks, it's corrected.

L246: "missMDA (not shown)" Any particular reason why this is not shown?
Response: Actually, both are shown so we removed the parenthetical reference (p. 12).

L250: Maybe I have missed something, but it was not clear to me why there are "highly different anthropogenic effects on the waterways" that affect "the river microbial consortia" between the River Yare samples (east and west) and the Vedder River and Vedder Canal samples. It might be good for the authors to briefly expand on this as not every reader will be familiar with the locality of these water bodies and their surrounding anthropogenic factors.
Response: We rearranged sentences and reworded this paragraph to address this issue, top of p. 12.

L258-260: I can not (yet) share the authors' conclusion "indicating that approximately 2.5x10^5 long reads appear necessary to adequately diagnose river function potential". Specifically, this would require a reply to my question about Figure4 (see below). It is not clear if the authors' performed rarefaction by considering the individual samples as random subsamples or if rarefaction was performed per sample. Given that there is a single line and model fit in Figure4, the former seems the case. However, the authors' statement is not sufficiently supported in my opinion then.
Response: The original figure for this point had 19 data points because it was made only for KO functions identified in all of the replicate runs for all of the rivers (all used as separate data sets). Given this Reviewer's comment, we have created a new chart showing all of the pooled samples as separate entities for KO, COG, and Subsystems. Thus, there now are 13 data points for each type of function. In each case, the inflection remains at 2.5x10^5 long reads or fewer. This is now Figure 3.

L268: Not sure why "all" is underlined and emphasized.
Response: Underline removed, thanks.

L306: Consider adding "and functions" to "epidemiologically significant organisms". This goes in line with the authors' studies of functional potential, especially AMR-related functions.
Response: Added at the top of p. 13.

L315: Rather keep details in the Results section instead. I do not think this detail is needed here to make the authors' point.
Response: We moved the alpha-diversity sentence to Results and slightly reworded this paragraph, p. 14.

L327-329: While anthropogenic effects linked to waste water discharge are intensively studied, I can not share the broad conclusion drawn by the authors here based on the presented results. Are there arguments that the authors could provide to strengthen their point? In particular, from what I could extract from the results, the "response" is only indirectly demonstrated, e.g., two sampling sites of the same river that exhibit different taxonomic composition and functional potential. However, which specific influences might lead to these differences are not presented. I see where the authors are coming from and I generally agree with their point-of-view, but I find this formulation too strong.
Response: We are receptive to this concern and in response we removed the broad conclusion, toned down this section, and focused instead on the utility of the approach, p. 14.

L356: Consider replacing "phenomenally" by a synonym, e.g., "substantially" or "pronouncedly".
Response: Done, p. 16.

L386: I am not sure, but is "Zealand, DE" correct? Or should it be "DK" instead?
Response: Thanks for pointing this out. The Skævinge wastewater treatment plant is indeed in Denmark (DK), as shown in Table 1. This has been corrected in the manuscript p. 17.

L459: Unfortunately, I was unable to retrieve the respective protocol from this DOI. Could the authors please check?
Response: Thanks for pointing this out. We did not include a full web address, as we believed that just the DOI was sufficient. We have updated these DOIs to a full link, i.e. ‘https://dx.doi.org/10.17504/protocols.io.qtgdwjw’ p. 20.

L507: Unfortunately, I was unable to retrieve the respective document from this DOI. Could the authors please check?
Response: Thanks for pointing this out. There was a transcription error in transferring the DOI from another document. The correct full link is ‘https://dx.doi.org/10.24433/CO.6736538.v1’ and has been corrected on p. 24.

Figure4: Could the authors please explain why there are more than 13 points shown even though 13 metagenomes were included in their study?
Response: This former Figure 4 contained results of replicate runs from several of the rivers. That figure is no longer included and the replacement only includes the runs shown in the text of the revised ms.

Figure5: Please consider putting the Pavian plots (Sankey plots) separately. They were impossible to read.
Response: Thanks for the suggestion; we have created two additional Supplementary Figures: 1) full-resolution pavian plots with river names underneath; 2) full-resolution location maps with inset reduced-resolution pavian plots.

Close
