# Supplementary material for: Metagenomic analysis of planktonic riverine microbial consortia using nanopore sequencing reveals insight into river microbe taxonomy and function
Source: Gigascience. 2020 Jun 10;9(6):giaa053. doi: 10.1093/gigascience/giaa053 (PMC7285869; doi:10.1093/gigascience/giaa053)
Supplement: giaa053_Reviewer_1_Report_Original_Submission — Daniel Read -- 11/28/2019 Reviewed [file giaa053_reviewer_1_report_original_submission.pdf]

Reviewer Report

Title: Metagenomic analysis of planktonic riverine microbial consortia using nanopore sequencing reveals insight into river microbe taxonomy and function

Version: Original Submission **Date:** 11/28/2019

Reviewer name: Daniel Read

Reviewer Comments to Author:

Reddington et al have produced a manuscript describing the application of Oxford Nanopore MinION metagenomic sequencing to describe the genetic taxonomic and functional potential of river water collected from 11 rivers sampled worldwide.

It is difficult to assess this study, as in many ways it is a methods paper, except that it lacks a critical comparison with the current standard approach that is used for metagenomics, Illumina short read sequencing. As such, the study lacks a rationale, other than to show that environmental metagenomic data can be collected using the minION sequencing platform (which has been done previously, although not admittedly for river samples). In addition, no contextual physicochemical data was collected from each river, so there is limited ecological or biogeographical inferences that can be made. Without high quality short read data as a comparison, it is hard to assess the strengths and weaknesses of this approach. Useful questions could address the utility of having longer reads generated by Nanopore sequencing. For example, does this result in a higher proportion of reads being functionally and taxonomically assigned? How does the lower read quality influence assignments? Do the longer reads assist with assembly based approaches? Although short read data may not be available as a comparison, a critical assessment of the strengths and weaknesses of this approach would improve the manuscript.

The other main issue is the focus on taxonomic assignment using metagenomic reads. Although this approach has been shown to work well in environments that are extremely well represented by reference genomes (for example, the human gut), it invariably does not work well in less well studied (and more diverse) environments (see https://environmentalmicrobiome.biomedcentral.com/articles/10.1186/s40793-019-0347-1 for an example of this with short read data). The taxonomic saturation of the rarefaction curves in figure 2 is likely a reflection of this. It would be interesting to compare metagenomic taxonomic assignment (via Kraken2) with taxonomy assignment using 16S rRNA genes extracted from the metagenomes.

The manuscript is on the whole well written, and I only have a few small comments below regarding the text. However, I think the plots and data visualisation could with improving:

1. Figure 1 does not need to be in the main manuscript and could be moved to supplementary.
2. Figure 5 is unreadable and needs a re-think. My preference would be to include site maps in the supplementary figures, and produce a bar chart (or series of bar charts and different taxonomic levels) highlighting the taxonomic differences in the samples.
3. Tables 1 and 2, could be moved to supplementary.
4. Tables 3 - 5, I recommend re-producing these as heatmaps, which would make them far easier to interpret.

Minor comments:
L169-170; All these families have representatives commonly found in freshwater.
L227; Picoplankton includes eukaryotes.

Methods

Are the methods appropriate to the aims of the study, are they well described, and are necessary controls included? Choose an item.

Conclusions

Are the conclusions adequately supported by the data shown? Choose an item.

Reporting Standards

Does the manuscript adhere to the journal's guidelines on minimum standards of reporting? Choose an item.

Choose an item.

Statistics

Are you able to assess all statistics in the manuscript, including the appropriateness of statistical tests used? Choose an item.

Quality of Written English

Please indicate the quality of language in the manuscript: Choose an item.

Declaration of Competing Interests

Please complete a declaration of competing interests, considering the following questions:

- Have you in the past five years received reimbursements, fees, funding, or salary from an organisation that may in any way gain or lose financially from the publication of this manuscript, either now or in the future?
- Do you hold any stocks or shares in an organisation that may in any way gain or lose financially from the publication of this manuscript, either now or in the future?
- Do you hold or are you currently applying for any patents relating to the content of the manuscript?
- Have you received reimbursements, fees, funding, or salary from an organization that holds or has applied for patents relating to the content of the manuscript?
- Do you have any other financial competing interests?
- Do you have any non-financial competing interests in relation to this paper?

If you can answer no to all of the above, write 'I declare that I have no competing interests' below. If your reply is yes to any, please give details below.

I declare that I have no competing interests

I agree to the open peer review policy of the journal. I understand that my name will be included on my report to the authors and, if the manuscript is accepted for publication, my named report including any attachments I upload will be posted on the website along with the authors' responses. I agree for my report to be made available under an Open Access Creative Commons CC-BY license (http://creativecommons.org/licenses/by/4.0/). I understand that any comments which I do not wish to be included in my named report can be included as confidential comments to the editors, which will not be published.

Choose an item.

To further support our reviewers, we have joined with Publons, where you can gain additional credit to further highlight your hard work (see: https://publons.com/journal/530/gigascience). On publication of this paper, your review will be automatically added to Publons, you can then choose whether or not to claim your Publons credit. I understand this statement.

Yes Choose an item.
