# Supplementary material for: Metagenomic analysis of planktonic riverine microbial consortia using nanopore sequencing reveals insight into river microbe taxonomy and function
Source: Gigascience. 2020 Jun 10;9(6):giaa053. doi: 10.1093/gigascience/giaa053 (PMC7285869; doi:10.1093/gigascience/giaa053)
Supplement: giaa053_Reviewer_2_Report_Original_Submission — CÃ©dric Laczny -- 12/9/2019 Reviewed [file giaa053_reviewer_2_report_original_submission.pdf]

**Reviewer Report**

**Title: Metagenomic analysis of planktonic riverine microbial consortia using nanopore sequencing reveals insight into river microbe taxonomy and function**

**Version: Original Submission** **Date:** 12/9/2019

**Reviewer name: CÃ©dric Laczny**

**Reviewer Comments to Author:**

# Summary
Reddington et al. present their work on using long read-based, whole genome sequencing of river-derived microbial consortia to study river ecology. This represents a timely and important work as it demonstrates the possibility of on-site sequencing for environmental monitoring. While the on-site data processing may (still) be challenging, e.g., due to very large reference databases, there might be ways to overcome this challenge, e.g., tailored databases or simply more powerful compute. Hence, being able to monitor not only the physico-chemical composition but also microbial composition and functional potential allows greater resolution of water quality, e.g., assessing the presence and abundance of relevant microorganisms, such as specific algae or specific bacteria. While Reddington et al. followed a homology-based approach to resolve taxonomy and function, I would have found it nice to see functions linked to taxonomy more strongly. This is something I would expect long read-based sequencing to be of particular use for, especially given that many of the taxa are lowly abundant. Having potentially several genes in synteny on an actual sequencing read avoids the need to perform de novo assembly, which is likely challenging given the dominantly lowly abundant taxa and thus limited sequencing coverage. However, I do not consider this a major limitation.
In general, I found the manuscript well to read, but encourage the authors to consider rearranging and/or reformulate specific parts to improve the readability further, as described in my Overall comments and Detailed comments. I think my comments should be straight-forward to be addressed by the authors as there are no additional experiments requested or the like, but mostly clarifications on individual aspects.
# Overall comments
- The structure of the manuscript could be improved. Please see also my comment on the position of the text on negative controls.
- It is nice to see that the authors extended their analyses beyond taxonomy and also study the functional potential. This is very relevant as similar functions may be encoded by distinct taxa or horizontal gene transfer events may occur or have occured affecting the phenotype. However, given that the vast majority of organisms was found in very low abundance (< 1%), in how much does the proposed approach improve over amplicon-based characterization? If only fractions of the majority of genomes are expected to be recovered, why perform whole-genome sequencing in the first place? If conclusions are drawn from the taxonomic assignments of the reads and not by population-resolved inference of the functional potential (e.g., via metagenome-assembled genomes), this can be more cheaply achieved using amplicon-based sequencing. Not that I am advocating for amplicon-based

sequencing in general. Quite the opposite, i.e., I would like to encourage the authors to highlight the added value of on-site _whole-genome_ sequencing more strongly, e.g., to resolve gene synteny directly from sequencing reads (rather than contigs) and/or to avoid primer bias or multiple sample processing to account for different "fractions" (16S, 18S, viral, etc.).
- While I found the ecological analyses limited, i.e., my main take-away was that anthropogenic factors may have a substantial effect on the community composition and presence of AMR-related genes - but there is likely much more than that in terms of ecology -, I understand that the focus of this study was on demonstrating the feasibility and usefulness of on-site sequencing, specifically using MinION-based sequencing. Hence, I am not sure if putting such a strong focus on the river ecology in the title is a good idea as other readers might have respective expectations. It is not a major comment though, but maybe the authors could think of a respectively adjusted title.
- I would encourage the authors to keep discussion points for the Discussion. This would reduce redundancy during reading.
# Detailed comments
L85-86: I think this could be even emphasized "and AMR genes, thus highlighting the importance/need to include microbial community data in broad-scale ecosystem models".
L89-90: Please include a respective reference or support this by the respective results.
L91-92: While I agree that field deployable sequencing is important, I am not sure if it is necessary or convenient. I would welcome further arguments to strenghten this.
L94: How about replacing "rapid" by "on-site"? "Rapid" is a rather relative term after all.
L98-100: Please clarify what particular advantage long-read data would bring. Importantly, I am not saying that this is not the case, but the authors could probably provide respective references easily to support their argument. Please also see my Overall comments.
L103-104: "To evaluate proof-of-concept" there seems to be something missing here. Maybe "a proof-of-concept"? For what? I assume field deployable sequencing?
L115: I find it unexpected that the Figure references start with "B&D", later followed by "A&C". Moreover, Table 2 comes before Table 1 was referred to. Albeit, Table 1 is referred to in the Methods, so this may depend on the particular specifications of the journal. This is clearly minor, but hopefully also easy to fix for the authors in the proofs, if the manuscript is accepted for publication.
L116-117: Maybe write "of total/cumulative length" or something along those lines?
L117: Maybe write "Rarefaction of the metagenomic data" or something along those lines?
L118: I highly welcome the inclusion of negative controls by the authors. However, I was confused by finding an elaboration on this important aspect only starting at L287. I would thus encourage the authors to move this aspect to near the beginning of the results as this is relevant when reading further. For example, I initially wondered whether the pronounced diversity in the "Chena" and "Corrib" samples might potentially partially be explained by contaminations which might have a disproportionally larger effect in low-biomass samples (e.g., river water).
L124-125: Please support this statement with respective results, e.g., a GC-distribution plot for the readers to better appreciate the extent of multimodality, etc.
L128: I found this to be confusing. The authors say that the majority of reads were of bacterial origin, but write a few sentences prior that One Codex was discarded due to a suspected deficiency in eukaryotic genomes in the underlying database? I am not advocating necessarily for the use of One

Codex, but rather want to highlight that this formulation is confusing and should be revised.
L143-144: I would suggest removing "A final group pf prokaryotes".
L147: Why not use "archaea" instead of "archaebacterial". Sticking with the former would be more consistent.
L151: I am not a native English speaker, so I had to look up "promulgator". It is referred to as "publisher". It is thus not clear to me how this word fits in this context. I would suggest using an alternative term to make it more accessible to non-native English readers.
L192: While Campylobacter, Clostridium, and Prevotella include species which are human pathogens, referring to these genera as pathogens is problematic in my opinion as it generalizes too much. If possible, I would encourage the authors to tone this somewhat done, as I do not see in how much this is necessary to be highlighted in their work. The text about "anthropogenically relevant" in the following is fair and informative, so should stay.
L204: Not sure what the authors mean by "The most common observation for 11 samples (virus accounting for <1% of reads) is typical of other river planktonic metagenomes". If this refers to the fact that virus reads are typically < 1%, how about replacing the parentheses by, "i.e.,"?
L211-212: How about using "... were virus-derived" or "were virus-borne"?
L212: What does "the ther" mean? I suspect it is a typo?
L246: "missMDA (not shown)" Any particular reason why this is not shown?
L250: Maybe I have missed something, but it was not clear to me why there are "highly different anthropogenic effects on the waterways" that affect "the river microbial consortia" between the River Yare samples (east and west) and the Vedder River and Vedder Canal samples. It might be good for the authors to briefly expand on this as not every reader will be familiar with the locality of these water bodies and their surrounding anthropogenic factors.
L258-260: I can not (yet) share the authors' conclusion "indicating that approximately 2.5x10^5 long reads appear necessary to adequately diagnose river function potential". Specifically, this would require a reply to my question about Figure4 (see below). It is not clear if the authors' performed rarefaction by considering the individual samples as random subsamples or if rarefaction was performed per sample. Given that there is a single line and model fit in Figure4, the former seems the case. However, the authors' statement is not sufficiently supported in my opinion then.
L268: Not sure why "all" is underlined and emphasized.
L306: Consider adding "and functions" to "epidemiologically significant organisms". This goes in line with the authors' studies of functional potential, especially AMR-related functions.
L315: Rather keep details in the Results section instead. I do not think this detail is needed here to make the authors' point.
L327-329: While anthropogenic effects linked to waste water discharge are intensively studied, I can not share the broad conclusion drawn by the authors here based on the presented results. Are there arguments that the authors could provide to strengthen their point? In particular, from what I could extract from the results, the "response" is only indirectly demonstrated, e.g., two sampling sites of the same river that exhibit different taxonomic composition and functional potential. However, which specific influences might lead to these differences are not presented. I see where the authors are coming from and I generally agree with their point-of-view, but I find this formulation too strong.
L356: Consider replacing "phenomenally" by a synonym, e.g., "substantially" or "pronouncedly".

L386: I am not sure, but is "Zealand, DE" correct? Or should it be "DK" instead?
L459: Unfortunately, I was unable to retrieve the respective protocol from this DOI. Could the authors please check?
L507: Unfortunately, I was unable to retrieve the respective document from this DOI. Could the authors please check?
Figure4: Could the authors please explain why there are more than 13 points shown even though 13 metagenomes were included in their study?
Figure5: Please consider putting the Pavian plots (Sankey plots) separately. They were impossible to read.

**Methods**

Are the methods appropriate to the aims of the study, are they well described, and are necessary controls included? Choose an item.

**Conclusions**

Are the conclusions adequately supported by the data shown? Choose an item.

**Reporting Standards**

Does the manuscript adhere to the journal's guidelines on minimum standards of reporting? Choose an item.

Choose an item.

**Statistics**

Are you able to assess all statistics in the manuscript, including the appropriateness of statistical tests used? Choose an item.

**Quality of Written English**

Please indicate the quality of language in the manuscript: Choose an item.

**Declaration of Competing Interests**

Please complete a declaration of competing interests, considering the following questions:

- Have you in the past five years received reimbursements, fees, funding, or salary from an organisation that may in any way gain or lose financially from the publication of this manuscript, either now or in the future?
- Do you hold any stocks or shares in an organisation that may in any way gain or lose financially from the publication of this manuscript, either now or in the future?

- Do you hold or are you currently applying for any patents relating to the content of the manuscript?
- Have you received reimbursements, fees, funding, or salary from an organization that holds or has applied for patents relating to the content of the manuscript?
- Do you have any other financial competing interests?
- Do you have any non-financial competing interests in relation to this paper?

If you can answer no to all of the above, write 'I declare that I have no competing interests' below. If your reply is yes to any, please give details below.

I declare that I have no competing interests

I agree to the open peer review policy of the journal. I understand that my name will be included on my report to the authors and, if the manuscript is accepted for publication, my named report including any attachments I upload will be posted on the website along with the authors' responses. I agree for my report to be made available under an Open Access Creative Commons CC-BY license (http://creativecommons.org/licenses/by/4.0/). I understand that any comments which I do not wish to be included in my named report can be included as confidential comments to the editors, which will not be published.

Choose an item.

To further support our reviewers, we have joined with Publons, where you can gain additional credit to further highlight your hard work (see: https://publons.com/journal/530/gigascience). On publication of this paper, your review will be automatically added to Publons, you can then choose whether or not to claim your Publons credit. I understand this statement.

Yes Choose an item.
